# High Grade of Amplification of Six Regions on Chromosome 2p in a Neuroblastoma Patient with Very Poor Outcome: The Putative New Oncogene *TSSC1*

**DOI:** 10.3390/cancers13225792

**Published:** 2021-11-18

**Authors:** Marzia Ognibene, Loredana Amoroso, Fraia Melchionda, Davide Cangelosi, Federico Zara, Stefano Parodi, Annalisa Pezzolo

**Affiliations:** 1 U.O.C. Genetica Medica, IRCCS Istituto Giannina Gaslini, 16147 Genova, Italy; federicozara@gaslini.org; 2 U.O.C. Oncologia Pediatrica, IRCCS Istituto Giannina Gaslini, 16147 Genova, Italy; loredanaamoroso@gaslini.org; 3 U.O. Pediatria, Oncologia e Ematologia Pediatrica, IRCCS Azienda Ospedaliero-Universitaria S. Orsola, 40138 Bologna, Italy; fraia.melchionda@aosp.bo.it; 4 Unità di Bioinformatica Clinica, IRCCS Istituto Giannina Gaslini, 16147 Genova, Italy; davidecangelosi@gaslini.org; 5 UOSID Epidemiologia e Biostatistica, IRCCS Istituto Giannina Gaslini, 16147 Genova, Italy; stefanoparodi@gaslini.org; 6 IRCCS Istituto Giannina Gaslini, 16147 Genova, Italy; annalisapezzolo56@gmail.com

**Keywords:** neuroblastoma, genomic amplification, *TSSC1*, gene expression, survival

## Abstract

**Simple Summary:**

Here, a case of neuroblastoma (NB) carrying a high-grade amplification of six loci besides *MYCN* is described. Since the patient had a very poor outcome, we postulated that these DNA co-amplifications might have a synergistic effect in increasing NB cell proliferation. In order to verify this hypothesis, we analyzed in silico the impact of high expression of the genes located within the amplifications on the NB patients’ outcome using a large dataset integrating three different platforms. These analyses disclosed that high expression of the *TSSC1* gene was the most significantly associated with reduced overall survival of NB patients, suggesting that it may have a potential prognostic role in NB in both *MYCN* amplified and *MYCN* not amplified tumors. Further studies on *TSSC1* interactions and functioning could lead to possible focused therapies for high-risk NB patients.

**Abstract:**

We observed a case of high-risk neuroblastoma (NB) carried by a 28-month-old girl, displaying metastatic disease and a rapid decline of clinical conditions. By array-CGH analysis of the tumor tissue and of the metastatic bone marrow aspirate cells, we found a high-grade amplification of six regions besides *MYCN* on bands 2p25.3–p24.3. The genes involved in these amplifications were *MYT1L*, *TSSC1*, *CMPK2, RSAD2*, *RNF144A, GREB1*, *NTSR2*, *LPIN1*, *NBAS,* and the two intergenic non-protein coding RNAs *LOC730811* and *LOC339788*. We investigated if these DNA co-amplifications may have an effect on enhancing tumor aggressiveness. We evaluated the association between the high expression of the amplified genes and NB patient’s outcome using the integration of gene expression data of 786 NB samples profiled with different public platforms from patients with at least five-year follow-up. NB patients with high expression of the *TSSC1* gene were associated with a reduced survival rate. Immunofluorescence staining on primary tumor tissues confirmed that the TSSC1 protein expression was high in the relapsed or dead stage 4 cases, but it was generally low in NB patients in complete remission. *TSSC1* appears as a putative new oncogene in NB.

## 1. Introduction

Neuroblastoma (NB) is an embryonal tumor arising from the neuronal crest cells of the sympathetic nervous system [1,2]. The overall genomic profile is a salient prognostic marker, which is used for treatment stratification of NB patients [2,3,4,5,6,7]. *MYCN* amplification is the most important genomic marker in terms of prognosis and impact on treatment decisions [1,2]. High *MYCN* expression deregulates the pathways of telomere lengthening, causing mitochondrial dysfunctions and replicative stress [8]. Amplification of *MYCN* occurs in 50% of high-risk NB cases, while high-risk *MYCN* single copy NB often express c-*MYC* as the oncogenic driver [9]. There is an incompatibility between *MYCN* amplification and other oncogenic events such as genomic rearrangements involving the *TERT* locus or mutations of the *ATRX* gene [10]. Other genomic amplifications targeting various loci, different from *MYCN*, were described [11,12,13,14,15,16,17,18]. The amplified loci, other than *MYCN*, observed in NB were: *ODC1* (2p25.1), *ALK* (2p23.2), *GREB1* (2p25.1), *NTSR2* (2p25.1), *TERT* (5p15.33), *LIN28B* (6q16.3), *MYC* (8q24.21), *MYEOV* (11q13.2), *CCND1* (11q13.3), *MDM2* (12q13.3), *CDK4* (12q14), and *FRS2* (12q15) [11,12,13,14,15,16,17,18]. Of note, NB patients with an amplicon not encompassing the *MYCN* locus have a very low survival probability [17], and these high-risk NB patients often display chemotherapy-resistant tumors. For the amplicons involving the genes *ODC1*, *ALK*, *CDK4*, *MDM2*, *MYC*, *TERT*, *FRS2,* and *CCND1,* inhibitors are currently being tested in clinical trials [19,20,21,22]. Little is known about the clinical characteristics and the survival of NB patients harboring genomic amplifications syntenic with *MYCN* [23,24]. *DDX1*, *NAG*, *NBAS*, and *GREB1* genes are frequently co-amplified with *MYCN* in NB primary tumors. However, the relationship between the high expression of these genes and NB patients’ prognosis is not shown to be significant [25]. *MYCN* amplification accompanied by multiple amplified loci within 2p, encompassing the genes *MYT1L*, *TSSC1*, *CMPK2*, *RSAD2*, *GREB1*, *NTSR2*, *LPIN1*, *LOC339788*, *LOC730811*, and *NBAS*, was reported in some individual cases of unfavorable NB [26]. Among these genes, *MYT1L* is of particular oncogenic interest in NB since it encodes for a zinc finger protein expressed in developing neurons at an early stage of differentiation [27], but it is not associated with unfavorable NB prognosis. Recently, in a study on the correlation between mitochondrial DNA haplogroups and susceptibility to NB, the expression of *CMPK2*, a nuclear gene participating in the mitochondrial pathway, was analyzed by RNA sequencing in 85 primary NB tumors, and the results showed over-expression in high-risk NB tumors [28].

Here we reported on a patient with high-risk stage 4 NB and adverse clinical behavior presenting a tumor carrying high-grade amplification of six loci besides *MYCN*, including the genes *MYT1L*, *TSSC1*, *CMPK2*, *RSAD2*, *RNF144A*, *GREB1*, *NTSR2*, *LPIN1*, *NBAS,* and the two long intergenic non-protein coding RNAs *LOC730811* and *LOC339788*. These findings open the way for the identification of putative new NB oncogene(s) in 2p25.3–p24.3 as possible targets for future therapies.

## 2. Materials and Methods

### 2.1. Case Description

A 28-month-old girl was referred to our examination for fever and lack of appetite associated with fatigue and asthenia. Physical examination findings included skin pallor, petechiae in the upper and lower limbs, tender and distended abdomen with hepatomegaly, and a palpable mass in the left iliac fossa. Ultrasonography showed a solid abdominal mass confirmed by a CT scan that showed a median retroperitoneal solid mass (11 × 10 × 13 cm) with the presence of a necrotic-colliquative component and fine calcifications, hepatomegaly with edema of the portal spaces, and the presence of at least three-millimeter focal lesions of likely secondary significance, respectively, to the IVs (7 mm), IIIs (3 mm), and VIs (4 mm). The blood test results were: hemoglobin 7.9 g/dL, leucocytes 28.50 × 10^3^/µL, piastrinopenia with a platelet count of 34 × 10^3^/µL, lactate dehydrogenase 6043 U/L, and activated protein C 17.32 mg/dL. The liver function tests revealed direct and total bilirubin levels of 1 and 1.65 mg/dL, respectively, elevated aspartate aminotransferase (AST) 449 U/L and a normal level of alkaline aminotransferase (ALT, 22 U/L). Urinary catecholamines were pathologic (homovanillic acid and vanillylmandelic acid 90.6 and 352.2 µmol/mmol, respectively). The patient presented a rapid decline in clinical conditions associated with severe hypoglycemia (20 mg/dL) and hyponatremia (123 mg/dL). At diagnosis, the patient had evidence of metastatic disease with massive infiltrating neoplastic cells in the bone marrow and was therefore considered as high-risk NB [29] and treated according to European HR-NBL1/SIOPEN protocol [30]. She received the first course of chemotherapy, but liver failure, impaired coagulation, oliguria that was unresponsive to diuretics, multi-organ failure, and progressive disease caused her death only 4 days after the diagnosis.

The pathological diagnosis was stroma poor, poorly differentiated, stage 4 NB. Primary NB tissue and metastatic bone marrow aspirate cells were obtained before treatment at the time of diagnosis. Tumor samples were stored in the BIT-NB (Biobank Integrated Tumor—Neuroblastoma) Tissue Section of IRCCS G. Gaslini, Genova, Italy. DNAs were extracted using QIAamp DNA Extraction Kit (Qiagen, Hilden, Germany), according to the manufacturer’s instructions.

### 2.2. Genomic Profile Analysis

DNAs from primary NB and metastatic bone marrow aspirate cells were tested by high-resolution oligonucleotide a-CGH using the 4 × 180 K Kit (Agilent Technologies, Santa Clara, CA, USA) with a mean resolution of approximately 25 kb [31]. Each hybridization produced a pair of 16-bit images, which were processed using the Agilent Feature Extraction 10.5 Software. The data were analyzed using the Genomic Workbench 7.0.40 software (Agilent Technologies, Santa Clara, CA, USA), the altered chromosomal regions and breakpoints events were detected using ADM-1 (threshold 10) with 0.5 Mb window size to reduce false positives [32]. Amplifications were defined at loci with log2 ratio ≥ 2, and loci with log2 ratio ≥3.5 were considered as high-level amplifications. Chromosome positions were determined using GRCh38/hg19 (UCSC Genome Browser, http://genome.ucsc.edu, accessed on 10 June 2021. The chromosomal copy number variations present in the Database of Genomic Variants (DGV: http://projects.tcag.ca/variation/, accessed on 28 July 2021) were taken into consideration only with a frequency <5%. The raw data were stored in the BIT-NB (Biobank Integrated Tumor—Neuroblastoma) Genomic Section of IRCCS G. Gaslini.

Written informed consent was obtained from the parents in accordance with the Declaration of Helsinki to report the case of their child. This study was approved by the Italian Institutional Ethics Committee (Measure n° 270/17 related to the clinical study protocol IGG-NCA-AP-2016).

### 2.3. Data Source

We inquired about any association between the high expression of the genes located within 2p amplifications and the NB patient’s survival using public gene expression data. A dataset of gene expression profiles from 786 NB primary tumors with associated patient clinical and molecular information was recently summarized by Cangelosi et al. [33]. This dataset was obtained integrating gene expression data from three different platforms, thereby representing the largest dataset of matched gene expression profiles and clinical data about NB patients available in the literature to date [33]. The dataset summarized by Cangelosi et al. was used to carry out gene expression and survival analyses of all genes except *NTSR2* and the two long non-coding mRNA (*LOC339788* and *LOC730811*), whose expression was not available in the dataset.

Analysis involving *NTSR2* gene and *LOC339788* and *LOC730811* loci were performed using the gene expression profile of 709 and 498 NB tumor samples generated by Agilent customized 4 × 44 K oligonucleotide microarray [34] and Illumina HiSeq 2000 platform [35], respectively. Gene expression and clinical data included in these datasets were publicly accessible through the ArrayExpress (http://www.ebi.ac.uk/arrayexpress; accession: E-MTAB-1781, accessed on 20 June 2021) and the Gene Expression Omnibus (http://www.ncbi.nlm.nih.gov/geo; accession: gse62564, accessed on 20 June 2021) databases.

The main characteristics of the selected datasets are resumed in Table 1. More information about the datasets structure and the original gene expression experiments are available in the related publications [33,34,35]. The level of expression of the target genes in the normal adrenal gland tissue, obtained from the Genotype-Tissue Expression (GTEx) database [36], is shown in Table S1.

### 2.4. Immunofluorescence Detection

Immunofluorescence analysis was performed on formalin-fixed, paraffin-embedded NB specimens (4 µm thick), as previously described [37]. TSSC1 protein expression and localization were revealed by incubation with the mouse monoclonal antibody TSSC1 (C7) (sc-376124 by Santa Cruz Biotechnology, Dallas, TX, USA) followed by a specific secondary antibody conjugated with Alexa 488 (green). We used isotype-matched non-binding mAbs in all antibody staining experiments to avoid nonspecific reactivity. Slides were counterstained with 4′,6′-diamidino-2-phenylindole (DAPI) (Vector Laboratories, Peterborough, UK) to visualize nuclei. The results were photographically documented using fluorescence microscope Axio Imager M2 equipped with ApoTome System (Carl Zeiss, Oberkochen, Germany). Each tested tumor area contained malignant NB cells, as assessed by histologic examination. The quantification of immunofluorescence positive tumor cells was performed on serial tissue sections, thus allowing quantification in tumor areas selected by the pathologist. TSSC1 protein is a component of the endosomal retrieval machinery, so green particles in the cytoplasm were considered to be positively stained cells following immunofluorescence detection. The results were analyzed according to the cell staining intensity and the percentage of TSSC1-positive cells. In total, 5 high-power fields were randomly selected for every section, and target cells were counted. The average percentage of 5 high-power fields was used for result determination.

### 2.5. Statistical Analysis

The expression levels of *LOC339788* non-coding mRNA were categorized into two categories because most expression values (*n* = 370, 74.3%) were tied to a single value equal to 0.0143553 arbitrary units, which allegedly corresponded to very low gene expression. Therefore, all analyses were performed by splitting the data on the basis of such cut-off. Expression levels of all the other genes were considered as continuous variables.

Differences between the expression of each considered gene, except *LOC339788*, by the patient prognostic factors at diagnosis (age, *MYCN* status, and stage) was evaluated by boxplot graphs and tested by the Mann–Whitney U test for pairwise comparisons and the Kruskal–Wallis test in the presence of more than two groups. With regard to *LOC339788* expression levels, differences by patient prognostic factors were assessed by the chi-squared test or the Fisher exact test when appropriated.

Association between gene expression and patient survival was evaluated by the Kaplan–Meier method. Values of each gene, except *LOC339788,* were split both into two and into three groups of equal sample size on the basis of the median and tertiles values, respectively, and the related 95% confidence intervals (95% CI) of survival estimates were obtained by the Kalbfleisch and Prentice method [38].

Both overall (OS) and Event Free survival (EFS) were estimated. Overall Survival time was calculated from the date of diagnosis to the date of death or to the last contact for censored data. Event Free survival was calculated from the date of diagnosis to the first occurrence between death and disease recurrence. No information was available about the occurrence of secondary tumors.

The statistical comparison between Kaplan–Meier curves was performed by using the log-rank test and by the log-rank test for trends when appropriated. The multivariable Cox regression model was also applied to adjust for the confounding effect of the available prognostic factors at diagnosis (*MYCN* status, age, and stage) [38]. The original non-categorized expression values were also analyzed by the Cox model to evaluate the presence of a linear trend between the gene expression and the patient survival.

The sub-group analyses included stratification by *MYCN* status and stage at diagnosis (localized, including stages 1, 2, and 3, stage 4 and stage 4S). Analyses restricted to the subgroup of patients with characteristics similar to those of the proband (i.e., age ≥ 18 months at diagnosis, Stage 4 and amplified *MYCN* status) were also performed.

All tests were two-tailed, and a *p*-value < 0.05 was considered statistically significant. All analyses were performed by STATA statistical software (release 13.1, Stata Corporation, College Station, TX, USA).

## 3. Results

### 3.1. Genomic Profile Analysis

**Figure 1:**
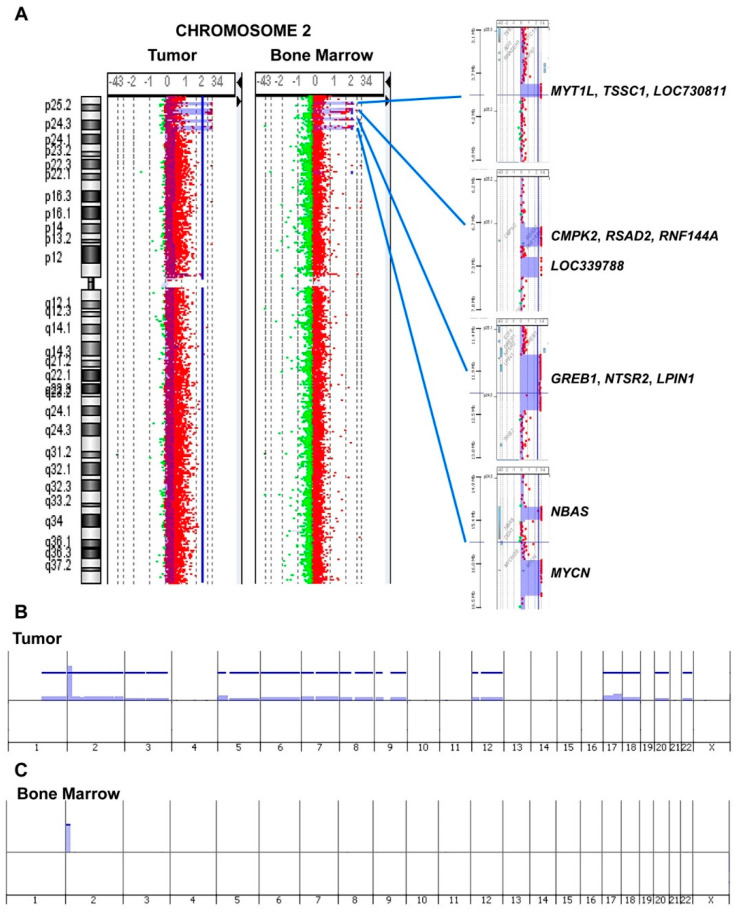
Genomic findings. (**A**) The six co-amplified regions on bands 2p25.3–p24.3 detected in NB primary tumor and in metastatic bone marrow aspirate cells from the patient. Genomic profiles obtained by array-CGH highlighted high level amplifications of six loci: 2p25.3, containing *MYT1L*, *TSSC1* and *LOC730811*; 2p25.2, containing *CMPK2*, *RSAD2*, and *RNF144A*; 2p25.1, containing *LOC339788*; 2p25.1–p24.3, containing *GREB1*, *NTSR2*, and *LPIN1*; 2p24.3, containing *NBAS*; 2p24.3, containing *MYCN*. (**B**) Primary tumor showed a genomic profile with recurrent segmental chromosome alterations (SCA profile) typical of NB such as 1q gain and 17q gain, the atypical segmental alteration 5p gain containing *TERT*, six concomitant regional co-amplifications on bands 2p25.3–p24.3, and 12 numerical chromosome alterations. (**C**) Metastatic bone marrow aspirate cells from the patient harbored only the same six co-amplified regions on bands 2p25.3–p24.3.

Molecular genomic profiling of tumor tissue led us to describe the concomitant high grade amplification of six loci located at: 2p25.3 (log2 ratio = 4.47) containing *MYT1L* and *TSSC1* genes and the long intergenic non-protein coding RNA *LOC730811*; 2p25.2 (log2 ratio = 4.38) containing *CMPK2*, *RSAD2*, and *RNF144A* genes; 2p25.1 (log2 ratio = 3.758) containing the long intergenic non-protein coding RNA *LOC339788*; 2p25.1–p24.3 (log2 ratio = 4.05) containing *GREB1*, *NTSR2*, and *LPIN1* genes; 2p24.3 (log2 ratio = 4.36) containing *NBAS* gene; 2p24.3 (log2 ratio = 4.29) containing *MYCN* gene (Figure 1A). Primary tumor harbored the segmental chromosome alterations typical of NB, 1q gain, and 17q gain, and the atypical 5p gain, containing the *TERT* gene, and several numerical alterations (Figure 1B). It is impossible to know if the tumor even showed a *TERT* rearrangement since only a-CGH data were available; however, these rearrangements are almost mutually exclusive with *MYCN* amplification. The six co-amplified regions on bands 2p25.3–p24.3 were detected, identical to the primary tumor, also in the metastatic bone marrow aspirate cells from the patient. The other cytogenetic abnormalities present in the primary tumor were not identified in the metastatic bone marrow cells (Figure 1A,C).

### 3.2. Association between Patient Survival and Expression of RNF144A

**Figure 2:**
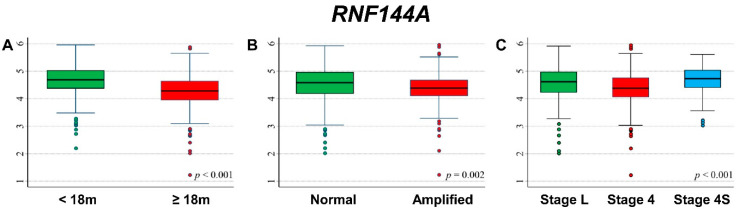
Association between *RNF144A* gene expression and the main patient characteristics at diagnosis. (**A**) Age (m = months); (**B**) *MYCN* status; (**C**) Stage (L = localized).

Association between patient characteristics at diagnosis and *RNF144A* expression levels are shown in Figure 2. Statistically significant higher values were observed for patients diagnosed before 18 months of age (Figure 2A), with normal *MYCN* status (Figure 2B), and with localized stages or stage 4S (Figure 2C).

The occurrence of both events and deaths were associated with lower expression values (Figure S1A,B, respectively). When stratifying by age at diagnosis, the difference was no longer significant (Figure S1C,D) but remained evident among patients with normal *MYCN* status (Figure S1E,F) and with stage 4 disease (Figure S1G,H) or localized stage (death occurrence only, Figure S1H).

**Figure 3:**
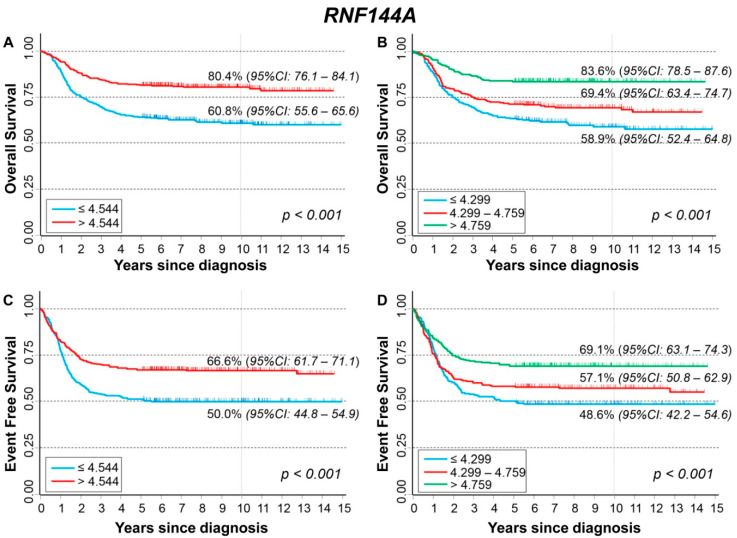
Patient Survival in relation to *RNF144A* expression level. Overall Survival of 786 NB patients with (**A**) cut-off based on the median expression value or (**B**) on tertile expression values. Event Free Survival of 769 NB patients with (**C**) cut-off based on the median expression value or (**D**) on tertile expression values. Ten-year survival estimates are displayed.

Figure 3A,B show the Overall Survival (OS) curves of the entire cohort of NB patients (*n* = 786) in relation to *RNF144A* expression levels. Higher values were associated with better survival (Figure 3A) with a statistically significant trend (Figure 3B). Multivariable analysis confirmed this finding (Table 2). Stratification by *MYCN* status (Table S2) indicates that the potentially protective effect of the high-level gene expression was limited to patients without *MYCN* amplification. The analysis by stage at diagnosis (Table S3) confirmed the better survival of patients with higher gene expression levels both in localized stages and in metastatic ones, except stage 4S, but for the latter, evidence was based on a very small number of deaths. Figure 3C,D show the Event Free Survival (EFS) of the cohort of 769 NB patients. Survival was better for higher values of *RNF144A* expression (Figure 3C) with evidence of a trend (Figure 3D). In multivariable analysis, this association was less evident and remains statistically significant only when the non-categorized original variable was considered (continuous variable) (Table 3). Table S4 shows the EFS stratified by *MYCN* status. The association between high gene expression levels and survival was observed only in patients with normal *MYCN* status. Analysis by stage at diagnosis (Table S5) confirmed the observed association among Stage 4 patients only.

The results of the analysis restricted to patients with stage 4 disease showed that amplified MYCN status and age ≥ 18 months (Figure S2) showed a better OS for patients with expression values above the median, but statistical significance was borderline (*p* = 0.054). No difference appears for the EFS or when data were stratified by tertile values (Figure S2B–D).

### 3.3. Association between Patient Survival and Expression of MYT1L

**Figure 4:**
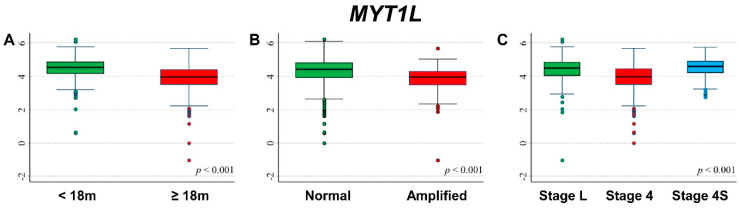
Association between *MYT1L* gene expression and the main patient characteristics at diagnosis. (**A**) Age; (**B**) *MYCN* status; (**C**) Stage.

The comparisons between *MYT1L* expression levels by patient characteristics at diagnosis are displayed in Figure 4. Lower values were associated with older age (Figure 4A), amplified *MYCN* status (Figure 4B), and stage 4 disease (Figure 4C).

Lower expression values were observed among patients with events (Figure S3A) and deceased (Figure S3B). This association was also observed after stratifying by age at diagnosis (Figure S3C,D), *MYCN* status (Figure S3E,F), and stage, with the only exception of Stage 4S patients (Figure S3G,H).

**Figure 5:**
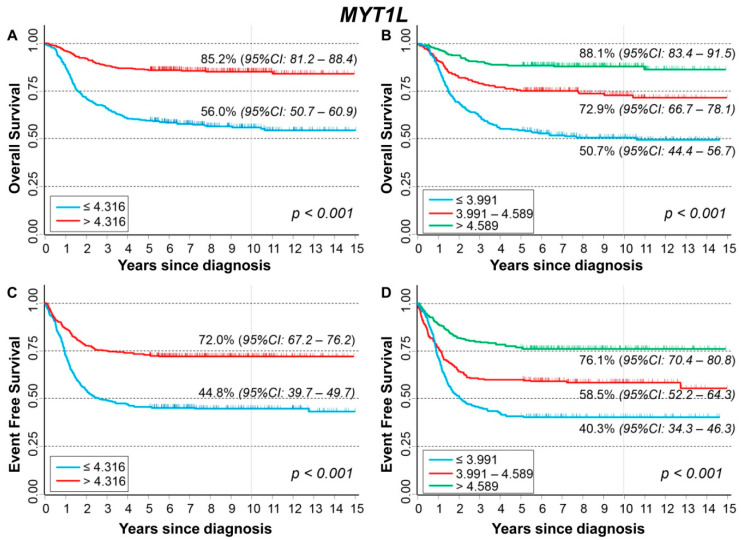
Patient Survival in relation to *MYT1L* expression level. Overall Survival of 786 NB patients with (**A**) cut-off based on the median expression value or (**B**) on tertile expression values. Event Free Survival of 769 NB patients with (**C**) cut-off based on the median expression value or (**D**) on tertile expression values. Ten-year survival estimates are displayed.

Figure 5 shows the OS curves (A–B) of the whole cohort of NB patients (n = 786) by *MYT1L* expression levels. A favorable outcome was associated with higher values of gene expression (Figure 5A) with clear evidence of trend (Figure 5B). This association was confirmed in multivariable Cox regression analysis (Table 4). Higher values of gene expression were found to be significantly associated with a better OS both in *MYCN* normal and in *MYCN* amplified NB (Table S6), and in both localized stages and Stage 4 patients, but not in Stage 4S (Table S7). EFS curves of the cohort of 769 NB patients indicated that survival was higher for higher values of *MYT1L* expression (Figure 5C) with clear evidence of trend (Figure 5D). Multivariable analysis confirmed this finding (Table 5). Higher gene expression values were associated with higher EFS both after stratifying by *MYCN* status (Table S8), and by stage at diagnosis, with the exception of Stage 4S patients (Table S9).

Analysis restricted to patients with stage 4 disease, amplified *MYCN* status, and age ≥ 18 months (Figure S4A–D) did not find any statistically significant association between patient survival and gene expression levels.

### 3.4. Association between Patient Survival and Expression of TSSC1

**Figure 6:**
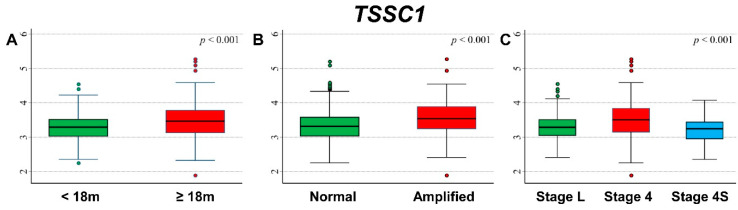
Association between *TSSC1* gene expression and the main patient characteristics at diagnosis. (**A**) Age; (**B**) *MYCN* status; (**C**) Stage.

Figure 6 shows the comparison between *TSSC1* expression levels by patient’s characteristics at diagnosis. Higher expression values were associated with older age (Figure 6A), amplified *MYCN* status (Figure 6B), and stage 4 disease (Figure 6C).

Higher values were associated with event occurrence (Figure S5A) and deceased patients (Figure S5B). Higher values were also associated with events in older patients (Figure S5C) and with death in both age groups (Figure S5D), with events in patients with *MYCN* amplification (Figure S5E), and with death in those with both amplified and not amplified *MYCN* status (Figure S5F). After stratification by stage at diagnosis, higher levels were observed for deceased patients in localized stages and in stage 4 (Figure S5H), whereas the association with event occurrence was no longer evident (Figure S5G).

**Figure 7:**
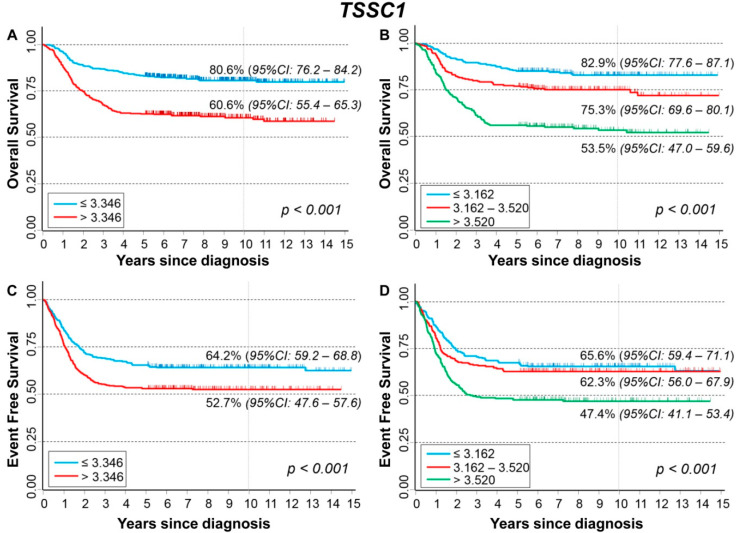
Patient Survival in relation to *TSSC1* expression level. Overall Survival of 786 NB patients with (**A**) cut-off based on the median expression value or (**B**) on tertile expression values. Event Free Survival of 769 NB patients with (**C**) cut-off based on the median expression value or (**D**) on tertile expression values. Ten-year survival estimates are displayed.

OS curves related to *TSSC1* expression levels reported in Figure 7 showed that a poorer outcome was associated with higher values of gene expression (Figure 7A), with evidence of trend (Figure 7B). The association was still observed and remained statistically significant after adjusting for the available confounding factors in multivariable Cox regression analysis (Table 6). The stratified analysis confirmed the observed association in both non-amplified and amplified *MYCN* patients (Table S10) and in both localized stages and stage 4 patients (Table S11). With EFS curves, we saw that survival was poorer for higher values of *TSSC1* expression (Figure 7C), with evidence of trend (Figure 7D). In multivariable analysis, this association was less noticeable and no longer statistically significant (Table 7). In patients with normal *MYCN* status, no statistically significant association was observed, whereas, in *MYCN* amplified NB, higher values of *TSSC1* expression were slightly associated with poorer survival (Table S12). Stratification by stage at diagnosis indicated that the association between the gene expression and patient survival was limited to Stage 4 patients (Table S13).

A poorer survival was observed in association with higher *TSSC1* expression values among patients with stage 4 disease, amplified *MYCN* status and age ≥ 18 months (Figure S6A–D), even if statistical significance was achieved for OS only when data were split on the median expression value (Figure S6A).

### 3.5. Association between Patient Survival and Expression of LOC730811

**Figure 8:**
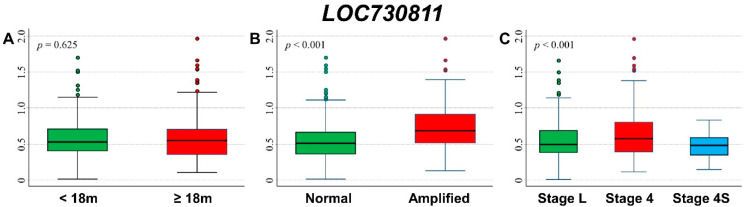
Association between *LOC730811* expression and the main patient characteristics at diagnosis. (**A**) Age; (**B**) *MYCN* status; (**C**) Stage.

Figure 8 shows the association between *LOC730811* expression and patient characteristics at diagnosis. Higher levels were observed among patients with amplified *MYCN* status (Figure 8B) and with stage 4 (Figure 8C).

Association between gene expression levels and the outcome (event or death) is shown in Figure S7A–H). No association was found with the occurrence of disease relapse in the whole cohort (Figure S7A) and after stratification by age (Figure S7C), *MYCN* status (Figure S7E), and stage (Figure S7G). Slightly higher expression values were observed among deceased patients (Figure S7B), which remains statistically significant among younger patients only (Figure S7D).

**Figure 9:**
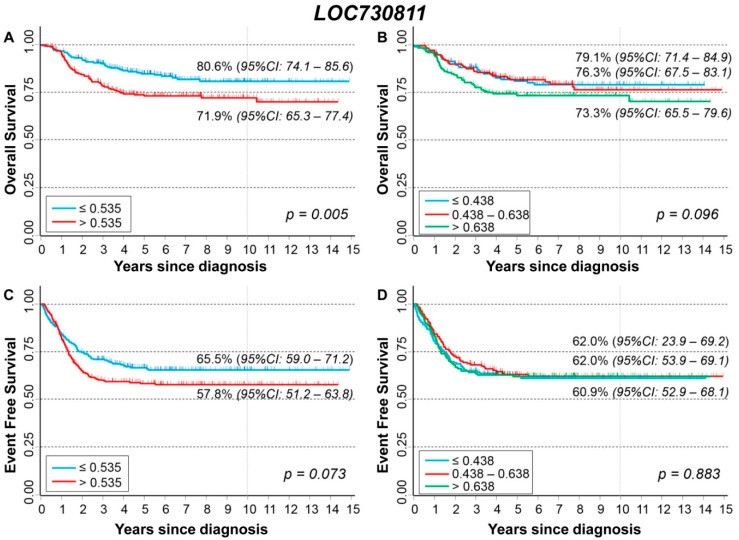
Patient Survival in relation to *LOC730811* expression level. Overall Survival of 498 NB patients with (**A**) cut-off based on the median expression value or (**B**) on tertile expression values. Event Free Survival of 498 NB patients with (**C**) cut-off based on the median expression value or (**D**) on tertile expression values. Ten-year survival estimates are displayed.

Figure 9 shows the OS of the whole cohort (n = 498) in relation to *LOC730811* long non-coding mRNA expression. A slightly poorer survival was associated with higher values of expression (Figure 9A) but without any evidence of trend (Figure 9B). After adjusting for the available confounding factors in multivariable Cox regression analysis, no association was observed (Table 8). After stratification by *MYCN* status (Table S14) and by stage at diagnosis (Table S15), no association between expression level and OS emerged. About EFS, no statistically significant association was found splitting the cohort on the basis of either the median (Figure 9C) or the tertile expression values (Figure 9D). The multivariable Cox regression analysis confirmed the lack of association between expression levels and EFS (Table 9). The results from the stratified analysis by *MYCN* status (Table S16) and by stage at diagnosis (Table S17) did not find any evidence of a potential impact of *LOC730811* on EFS.

The analysis restricted to patients with stage 4 disease, amplified *MYCN* status, and age ≥ 18 months (Figure S8A–D) shows a poorer EFS in relation to higher expression levels (Figure S8B), but without evidence of any trend (Figure S8D).

### 3.6. Association between Patient Survival and Expression of CMPK2

**Figure 10:**
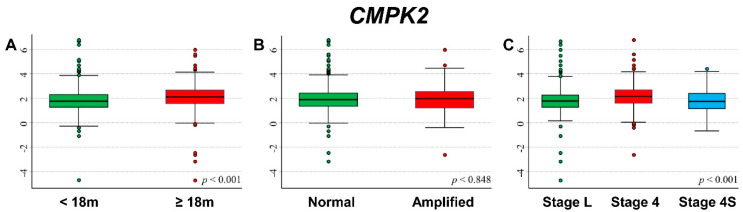
Association between *CMPK2* gene expression and the main patient characteristics at diagnosis. (**A**) Age; (**B**) *MYCN* status; (**C**) Stage.

Figure 10 shows the association between *CMPK2* gene expression and the main patient characteristics at diagnosis. Higher expression values were observed among older patients (Figure 10A) and in those with stage 4 at diagnosis (Figure 10C).

Figure S9 shows the association between *CMPK2* expression levels and patient outcome. The occurrence of both event (Figure S9A) and dead (Figure S9B) were related to high expression values. This association was no more observed after stratification by age (Figure S9C,D) and stage (Figure S9G,H) but remains evident and statistically significant in patients with normal *MYCN* status (Figure S9E,F).

**Figure 11:**
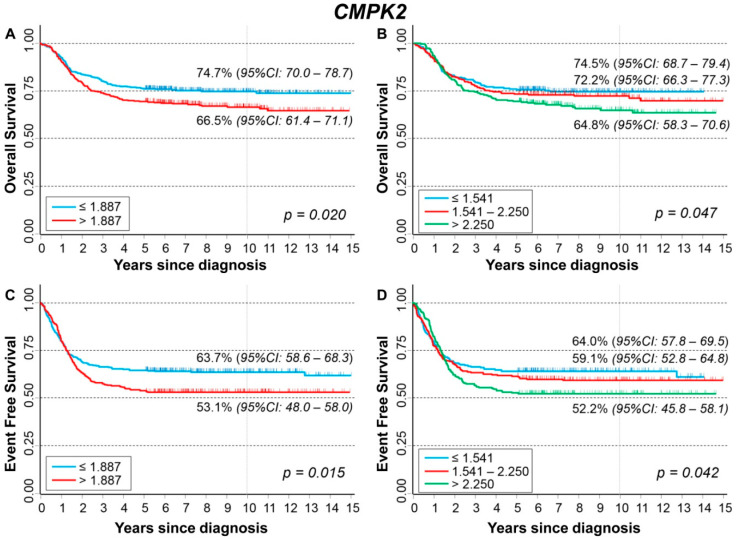
Patient Survival in relation to *CMPK2* expression level. Overall Survival of 786 NB patients with (**A**) cut-off based on the median expression value or (**B**) on tertile expression values. Event Free Survival of 769 NB patients with (**C**) cut-off based on the median expression value or (**D**) on tertile expression values. Ten-year survival estimates are displayed.

In Figure 11, we reported the survival curves of the entire cohort of NB patients in relation to *CMPK2* expression levels. OS was slightly poorer in association with a high level of gene expression (Figure 11A) with some evidence of trend (Figure 11B). This association was no longer evident after adjusting for confounding in multivariable Cox regression analysis (Table 10). The stratified analysis highlighted a lower survival in relation to higher values of gene expression among patients with normal *MYCN* status (Table S18) and in those diagnosed in stage 4, even if in the latter no clear evidence of trend emerged (Table S19). EFS of the cohort of 769 NB patients in relation to *CMPK2* expression levels displayed a survival slightly poorer in patients with higher values of gene expression (Figure 11C) with some evidence of trend (Figure 11D). In multivariable analysis, this association was no more evident (Table 11). Higher levels of gene expression were associated with a poorer prognosis among non-amplified *MYCN* patients, but not for the ones with *MYCN* amplification (Table S20). Stratification by stage at diagnosis did not find any statistically significant correlation (Table S21).

Analysis of the group of patients with amplified *MYCN* status, stage 4 disease, and age ≥ 18 months at diagnosis did not find any association between patient survival and *CMPK2* expression values (Figure S10).

### 3.7. Association between Patient Survival and Expression of RSAD2

**Figure 12:**
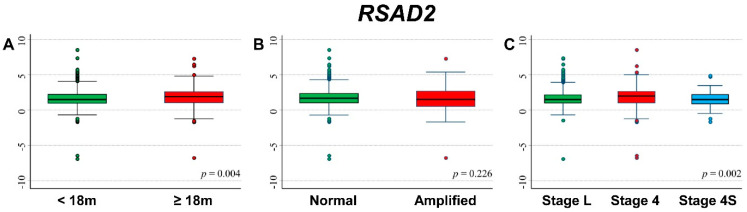
Association between *RSAD2* gene expression and the main patient characteristics at diagnosis. (**A**) Age; (**B**) *MYCN* status; (**C**) Stage.

Figure 12 shows the association between *RSAD2* gene expression and the main patient characteristics at diagnosis. Significantly higher values were observed among older patients (Figure 12A) and stage 4 patients (Figure 12C).

Gene expression values were not associated with patient outcome (Figure S11A–H) except for slightly lower values in localized stages among deceased patients both for event (Figure S11G) and for dead occurrence (Figure S11H).

**Figure 13:**
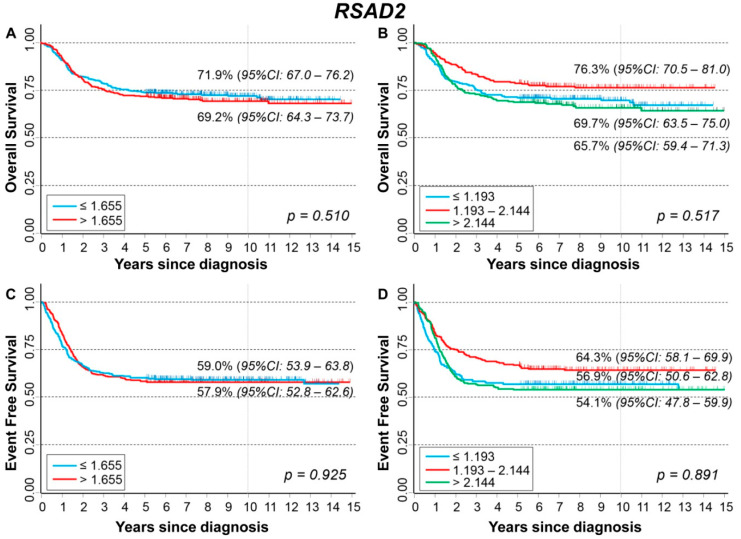
Patient Survival in relation to *RSAD2* expression level. Overall Survival of 786 NB patients with (**A**) cut-off based on the median expression value or (**B**) on tertile expression values. Event Free Survival of 769 NB patients with (**C**) cut-off based on the median expression value or (**D**) on tertile expression values. Ten-year survival estimates are displayed.

Figure 13 shows the survival curves of the entire cohort of NB patients in relation to *RSAD2* expression levels. No association was found after splitting the cohort either on the basis of the median (Figure 13A) or the tertiles (Figure 13B) of the gene expression distribution. Multivariable analysis confirmed the lack of any association (Table 12). Neither stratification by *MYCN* status (Table S22) nor by stage at diagnosis (Table S23) found any significant link between OS and *RSAD2* expression. About EFS, no association emerged splitting the data by either median (Figure 13C) or tertile gene expression values (Figure 13D). In multivariable analysis, no clear link between *RSAD2* and EFS was observed (Table 13). *RSAD2* expression was not found associated with EFS either in normal or in amplified *MYCN* patients (Table S24). Among patients with localized disease, a poorer survival was observed for those with high values of gene expression (Table S25).

Restriction to patients with age ≥ 18 months at diagnosis, stage 4, and amplified *MYCN* status did not find any association between *RSAD2* expression levels and patient survival (Figure S12).

### 3.8. Association between Patient Survival and Expression of LOC339788

*LOC339788* expression levels were analyzed after collapsing all values into two categories using as a cut-off the baseline value. Values above the cut-off were more frequently in *MYCN* amplified patients (35% vs. 23%, *p* = 0.024), while no difference was observed by age and stage at diagnosis (Table 14).

No association was observed between *LOC339788* expression values and patient outcome except for a slightly higher proportion of observed events in patients with higher expression values compared to those with a lower expression both in the subgroup of aged < 18 months (30% vs. 20%, *p* = 0.045) and in those diagnosed with a localized stage (30% vs. 18%, Table S26).

**Figure 14:**
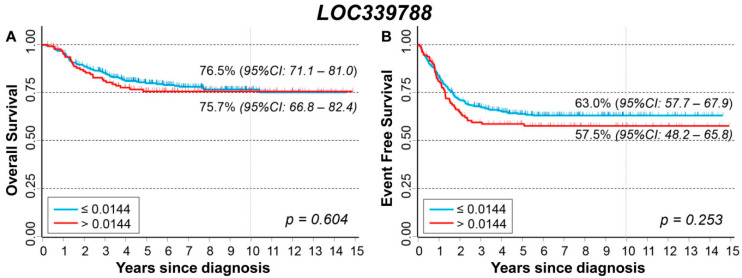
(**A**) Overall Survival of 498 NB patients in relation to *LOC339788* expression levels. The selected cut-off is based on the baseline expression value. (**B**) Event Free Survival of 498 NB patients in relation to *LOC339788* expression levels. The selected cut-off is based on the baseline expression value.

Figure 14A shows the OS curves of the entire cohort of NB patients (n = 498) in relation to the expression of the *LOC339788* long non-coding mRNA. No association was found. Multivariable analysis confirmed this observation (Table 15). Stratified analysis either by *MYCN* status (Table S27) or by stage at diagnosis (Table S28) did not find any link between *LOC339788* expression and patient survival. Figure 14B, reporting EFS curves, shows that no association emerged. Multivariable analysis confirmed the lack of any association (Table 16). Stratified analysis by *MYCN* status (Table S29) and stage at diagnosis (Table S30) did not find any link between *LOC339788* expression levels and EFS except a slightly poorer outcome among localized stages patients with higher values.

### 3.9. Association between Patient Survival and Expression of GREB1

**Figure 15:**
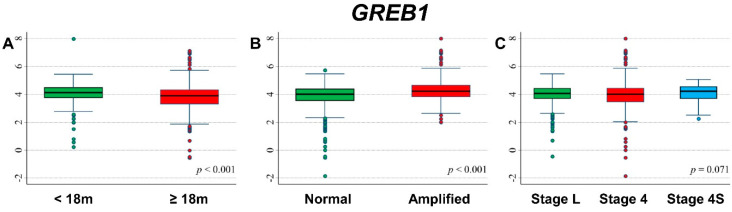
Association between *GREB1* gene expression and the main patient characteristics at diagnosis. (**A**) Age; (**B**) *MYCN* status; (**C**) Stage.

Figure 15 shows the association between *GREB1* gene expression and the main patient characteristics at diagnosis. Slightly higher values were observed among younger patients (Figure 15A) and in *MYCN* amplified (Figure 15C).

Association between gene expression and patient outcome is shown in Figure S13. Slightly higher values were observed among patients without events (Figure S13A). In stratified analysis, among patients with normal *MYCN* status, higher values were observed for patients who did not experience events (Figure S13E) and for those alive (Figure S13F). A similar association was found among patients with a localized stage at diagnosis (Figure S13G,H).

**Figure 16:**
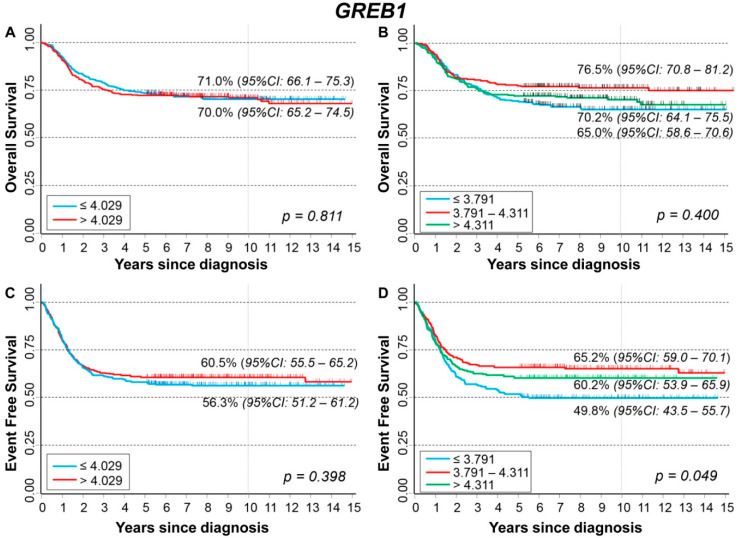
Patient Survival in relation to *GREB1* expression level. Overall Survival of 786 NB patients with (**A**) cut-off based on the median expression value or (**B**) on tertile expression values. Event Free Survival of 769 NB patients with (**C**) cut-off based on the median expression value or (**D**) on tertile expression values. Ten-year survival estimates are displayed.

Figure 16 shows the OS curves of the whole cohort (n = 786), referred to as *GREB1* expression levels. No association was observed either splitting data on median expression values (Figure 16A) or on the basis of tertile values (Figure 16B). Multivariable analysis confirmed the lack of any association (Table 17). Higher values were associated with a better OS in non-amplified *MYCN* patients, with evidence of a trend, but not in the ones carrying *MYCN* amplification (Table S31). Analysis by stage at diagnosis found a positive association between *GREB1* expression levels and OS in localized stages (Table S32). About EFS curves related to *GREB1* expression, no association was found when data were split on the basis of the median value of the expression (Figure 16C), while a better survival was observed for higher values when data were split on the tertiles of the distribution, but statistically, the significance was borderline (Figure 16D). In multivariable Cox regression analysis, a slightly statistically significant linear trend was also observed (Table 18). High levels of *GREB1* expression were linked to better survival in patients with normal *MYCN* status (Table S33) and in those diagnosed with localized stages, the latter with a borderline statistical significance (Table S34).

Analysis for the group of patients diagnosed at age ≥ 18 months, with amplified *MYCN* status and stage 4 disease, did not find any association between *GREB1* expression levels and patient survival (Figure S14A–D).

### 3.10. Association between Patient Survival and Expression of NTSR2

**Figure 17:**
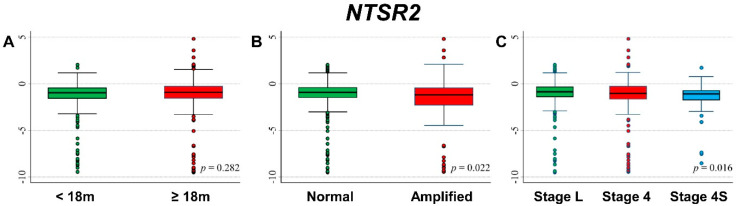
Association between *NTSR2* gene expression and the main patient characteristics at diagnosis. (**A**) Age; (**B**) *MYCN* status; (**C**) Stage.

Figure 17 shows the association between *NTSR2* gene expression and the main patient characteristics at diagnosis. Slightly lower values were observed for patients with amplified *MYCN* status (Figure 17B) and stage 4S disease (Figure 17C).

No statistically significant association was found between gene expression values and the occurrence of event or death in the whole cohort (Figure S15A,B, respectively) and after stratification by age at diagnosis (Figure S15C,D), *MYCN* status (Figure S15E,F), and stage (Figure S15G,H).

**Figure 18:**
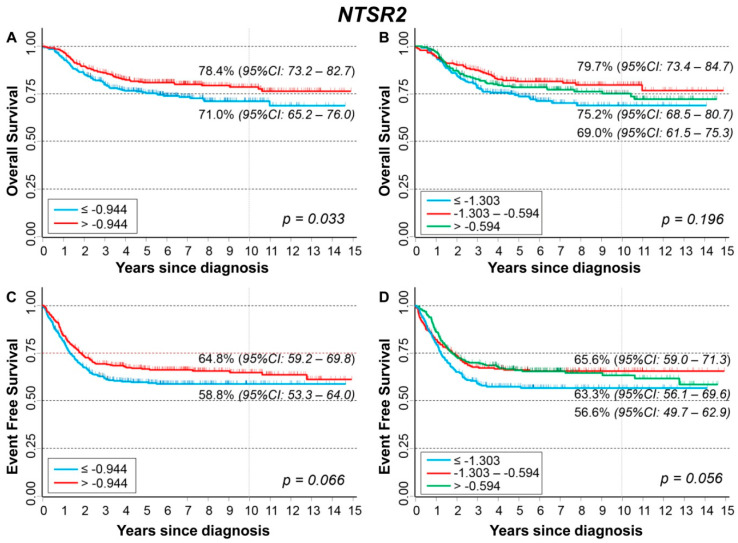
Patient Survival in relation to *NTSR2* expression level. Overall Survival of 709 NB patients with (**A**) cut-off based on the median expression value or (**B**) on tertile expression values. Event Free Survival of 695 NB patients with (**C**) cut-off based on the median expression value or (**D**) on tertile expression values. Ten-year survival estimates are displayed.

In Figure 18, we reported the OS curves of the entire cohort of NB patients (n = 709) in relation to *NTSR2* expression levels. Better survival was slightly associated with higher values of gene expression (Figure 18A) but without any evidence of trend (Figure 18B). No statistically significant association was found in multivariable Cox regression analysis (Table 19). The stratified analysis did not reveal any effect either in *MYCN* normal or in *MYCN* amplified patients (Table S35), while high expression levels were found associated with better survival in the group of localized stages (Table S36). Regarding EFS curves, survival was slightly better for higher values of *NTSR2* expression (Figure 18C,D), but statistical significance was not reached. The multivariable analysis did not find any link between the gene expression and EFS (Table 20). Stratified analysis by *MYCN* status did not find any association (Table S37). Among patients with a localized stage at diagnosing, a better survival in relation to higher levels of gene expression was observed (Table S38).

Analysis restricted to patients with age ≥ 18 months at diagnosis, stage 4 disease, and *MYCN* amplification did not find any association between patient survival and *NTSR2* expression levels (Figure S16).

### 3.11. Association between Patient Survival and Expression of LPIN1

**Figure 19:**
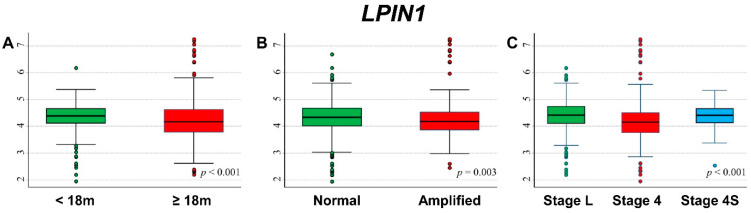
Association between *LPIN1* gene expression and the main patient characteristics at diagnosis. (**A**) Age; (**B**) *MYCN* status; (**C**) Stage.

Figure 19 shows the association between *LPIN1* gene expression and the main patient characteristics at diagnosis. Lower values were observed for patients aged 18 months or more at diagnosis (Figure 19A), with amplified *MYCN* status (Figure 19B), and with stage 4 disease (Figure 19C).

Lower expression values were observed for patients with events (Figure S17A) and for deceased ones (Figure S17B). In stratified analysis, such associations were observed among patients ≥ 18 months at diagnosis (Figure S17C,D), with normal *MYCN* status (Figure S17E,F), and in those with a localized stage (Figure S17G,H).

**Figure 20:**
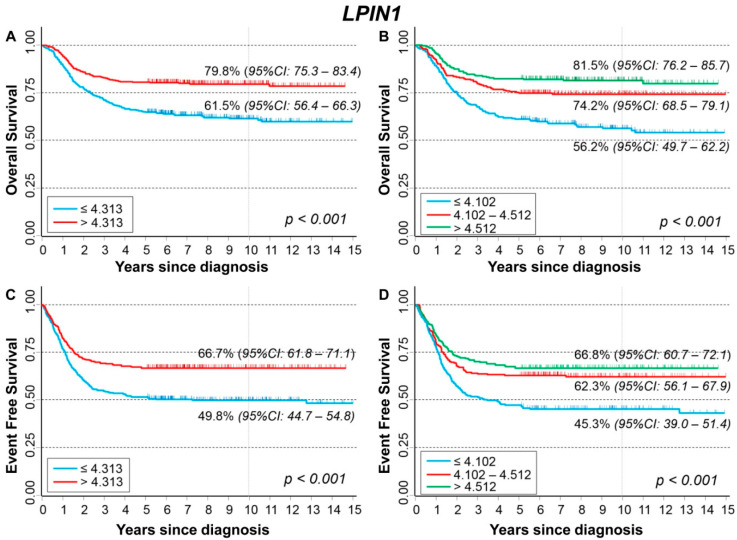
Patient Survival in relation to *LPIN1* expression level. Overall Survival of 786 NB patients with (**A**) cut-off based on the median expression value or (**B**) on tertile expression values. Event Free Survival of 769 NB patients with (**C**) cut-off based on the median expression value or (**D**) on tertile expression values. Ten-year survival estimates are displayed.

OS curves of the entire cohort of NB patients in relation to *LPIN1* expression levels (Figure 20) showed a positive association between patient survival and gene expression levels, with higher values of *LPIN1* expression corresponding to better OS (Figure 20A) and evidence of trend (Figure 20B). In multivariable analysis, the observed association was slightly attenuated, but the trend remains statistically significant (Table 21). The link between higher OS and *LPIN1* levels seemed to be limited to patients with normal *MYCN* status (Table S39) and diagnosed in localized stages (Table S40). EFS curves of the cohort of 769 NB patients related to *LPIN1* expression showed that survival was better for higher values of gene expression (Figure 20C) with evidence of trend (Figure 20D). In multivariable analysis, this association was reduced, and statistical significance was limited to the coefficient of the continuous variable (Table 22). Stratified analysis showed a link between high values of *LPIN1* expression and patient survival in the group with normal *MYCN* status (Table S41) and for patients with an NB diagnosis in localized stages (Table S42).

Among patients ≥ 18 months at diagnosis, with *MYCN* amplified tumor and Stage 4 disease (Figure S18A–D), no association was found between *LPIN1* expression levels and patient survival.

### 3.12. Association between Patient Survival and Expression of NBAS

**Figure 21:**
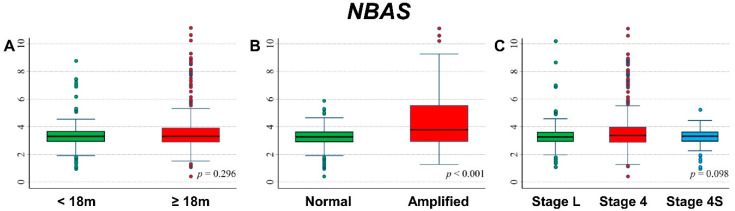
Association between *NBAS* gene expression and the main patient characteristics at diagnosis. (**A**) Age; (**B**) *MYCN* status; (**C**) Stage.

Figure 21 shows the association between *NBAS* gene expression and the main patient characteristics at diagnosis. Statistically significant higher values were observed for patients with *MYCN* amplified tumors (Figure 21B).

Slightly higher expression values were associated with a poorer outcome (event, Figure S19A; death, Figure S19B). However, this association was no longer evident after stratification by age at diagnosis (Figure S19C,D), *MYCN* status (Figure S19E,F), and stage (Figure S19G,H), except for death occurrence among older patients (Figure S19D), and stage 4 patients (Figure S19H).

**Figure 22:**
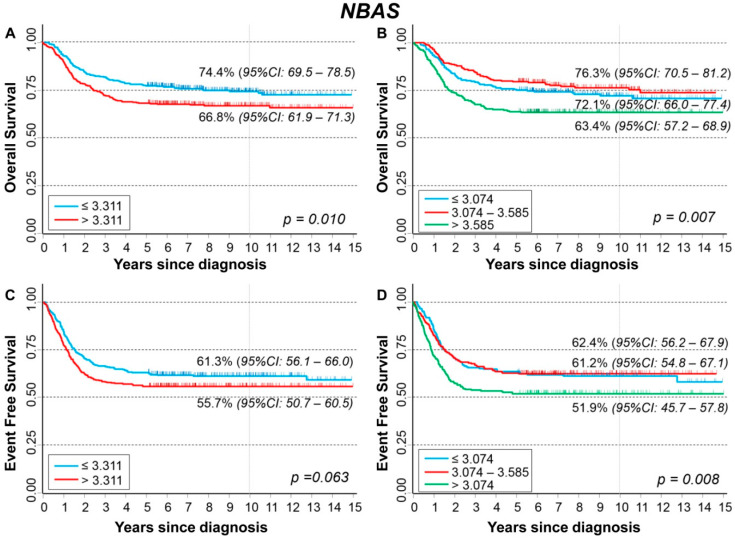
Patient Survival in relation to *NBAS* expression level. Overall Survival of 786 NB patients with (**A**) cut-off based on the median expression value or (**B**) on tertile expression values. Event Free Survival of 769 NB patients with (**C**) cut-off based on the median expression value or (**D**) on tertile expression values. Ten-year survival estimates are displayed.

Figure 22 shows the OS curves of the whole cohort of NB patients (n = 786) related to *NBAS* expression levels. Survival was inversely associated with gene expression because higher values corresponded to a poorer OS (Figure 22A,B). In multivariable analysis via Cox regression model, the observed association was reduced, and the statistical significance was completely lost (Table 23). No association emerged stratifying by *MYCN* status (Table S43), while a slight inverse significant association was observed in Stage 4 patients (Table S44). EFS curves showed a poorer survival associated with higher values of *NBAS* expression (Figure 22C), with some evidence of trend (Figure 22D). The association was no longer observed in multivariable analysis (Table 24). The stratified analysis did not find any association splitting the data either by *MYCN* status (Table S45) or by stage at diagnosis (Table S46).

Analysis restricted to patients with age ≥ 18 months at diagnosis, stage 4 disease, and *MYCN* amplified tumor did not find any association between patient survival and *NBAS* expression levels (Figure S20A–D).

### 3.13. TSSC1 Protein Expression in Stage 4 NB

**Figure 23:**
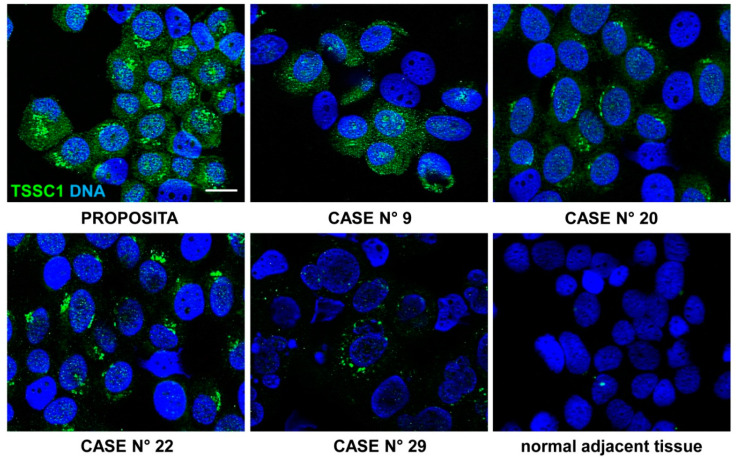
Expression of TSSC1 protein in stage 4 primary NB tissues. Immunofluorescence assay was performed using the anti-TSSC1 antibody (green). Images are representative of TSSC1 expression on the proposita tumor tissue (highly positive) and on other four stage 4 NB tissues among the 30 examined. Case number 29 showed a lower amount of TSSC1 positive cells. Normal adjacent tissue displayed very low expression of TSSC1. Cells were counterstained with DAPI to visualize nuclei (blue). (Scale bar: 10 μm).

In order to evaluate TSSC1 protein expression, we performed immunofluorescence analysis of the proposita tumor tissue and of other 30 stage 4 NB biopsy tissue samples at the onset of disease. The results of TSSC1 protein expression analysis in the observed specimens revealed that TSSC1 was mainly expressed in the cytoplasm and showed low expression in adjacent normal tissue. TSSC1 expression was estimated by evaluating the proportion of positively stained NB cells. TSSC1 was found expressed with a high percentage of positive cells (>85%) in the proposita NB tissue. Similarly, all relapsed or dead stage 4 NB showed many TSSC1 positive cells in their tumor tissues, while NB in complete remission expressed a lower amount of TSSC1 positive cells (Figure 23 and Table 25). In more detail, the median value was 12.5 among the not relapsed patients and 67.5 among the relapsed ones (*p* < 0.001, Mann–Whitney U test); 11.5 among the 10 alive patients and 67.58 among the 20 deceased ones (*p* < 0.001) (Figure S21).

## 4. Discussion

We observed a case of NB showing high-grade amplification of six loci besides *MYCN* in a 28-month-old girl with high-risk metastatic disease. The large regional amplifications on bands 2p25.3–p24.3 involved the genes *MYT1L*, *TSSC1*, *CMPK2*, *RSAD2*, *RNF144A*, *GREB1*, *NTSR2*, *LPIN1*, *NBAS,* and the two long intergenic non-protein coding RNAs *LOC730811* and *LOC339788*. The six co-amplified regions on bands 2p25.3–p24.3, identical to the primary tumor, were also detected in metastatic bone marrow aspirate cells from the patient. This observation suggests that the multiple co-amplifications located on 2p occurred in the first steps of tumor development, where the immature cells that are the source of NB are still present in the sympathetic nervous system. High-level amplifications may arise by different mechanisms such as chromothripsis or breakage-fusion bridges, leading to proto-oncogenes amplified in homogeneously staining regions or in extrachromosomal circular DNA (double minute chromosome) [39]. The increased expression of one or more proto-oncogenes contained in highly amplified regions contributes to tumor formation, maintenance, progression, and resistance to therapies. NB is one of the first cancers in which amplification of the *MYCN* proto-oncogene in the form of extrachromosomal circularization has been described [40]. The extrachromosomal circular DNA could constitute the driving mutagenic process in NB with functional consequences beyond *MYCN* amplification. Recently, a large complexity of extrachromosomal circular DNA, with hot spots on chromosome 2p, was described in NB using whole-genome sequencing (WGS) data [41]. The map of extrachromosomal circular DNA in NB describes how the DNA circularization affects not only proto-oncogenes but also various coding and non-coding regions with yet unknown functional effects [41]. The patient presented a rapid decline of clinical conditions that led her to death after only four days since diagnosis. Primary NB tumor harbored, in addition to high-grade amplification of six loci besides *MYCN*, the atypical 5p15 gain containing *TERT* gene. It is known that in non-*MYCN*-amplified NB displaying 5p15 gains, *TERT* expression increases, functioning in lieu of *MYCN* amplification to promote tumor progression [42]. Although nothing is known about the influence of 5p15 gains in *MYCN*-amplified NB, it is possible that the amplified expression of *TERT* also contributed to the very poor prognosis of the patient. We postulated that the co-amplifications of genes located on chromosome 2p could functionally cooperate with *MYCN* amplification increasing NB cell proliferation and tumor aggressiveness. In order to understand which gene(s) could be interacting with *MYCN* that caused such adverse effects on the proposita, we analyzed in silico the impact of high expression of the genes located within the amplifications on the NB patients’ outcome using the information contained in the dataset summarized by Cangelosi et al. [33]. These analyses disclosed that high expression of the *TSSC1* gene resulted most significantly related to a reduced survival rate of NB patients, suggesting that it may have a strong oncogenic role in NB. The association remains highly statistically significant after adjusting for age, stage, and *MYCN* status. Interestingly, the stratified analysis found a very poor survival in association with high expression values of *TSSC1* gene among patients with clinical characteristics similar to those of our patient, i.e., metastatic disease, age > 18 months, and *MYCN* amplified. While the expression of *RNF144A*, *LPIN1,* and *MYT1L* genes was inversely associated with both OS and EFS of NB patients, the stratified analysis indicated a potentially protective effect of *LPIN1* and *MYT1L* gene expression in localized tumors, especially in patients with normal *MYCN* status. *RNF144A*, *LPIN1,* and *MYT1L* genes, therefore, behave as tumor suppressor genes; in particular, the pan neuron-specific transcription factor *MYT1L* exerts an important function in neuronal differentiation [20,43], and its overexpression could lead to a differentiation of NB cells with a beneficial effect for patients’ outcome.

To confirm the negative effect of the *TSSC1* gene on the survival of the NB patients, we showed by immunofluorescence analysis that *TSSC1* protein expression was high in the tumor tissues of relapsed or dead stage 4 patients, but it was generally low in NB cases in complete remission. This data induced us to hypothesize that *TSSC1* could be a new putative oncogene whose high expression strongly enhances the inauspicious action of *MYCN*. Interestingly, in some tumors with non-amplified *MYCN* (breast cancer, Wilms tumor), *TSSC1* behaves as a tumor suppressor gene instead of an oncogene [44].

*TSSC1* gene plays a critical role in the endosomal retrieval pathway, and it is identified as a component of the molecular machinery that catalyzes cargo recycling to the plasma membrane [45]. TSSC1 participates in a network of interactions with SNARE (Soluble N-ethylmaleimide Attachment REceptors) proteins, consistent with the role of these proteins in membrane fusion of endosome-derived carriers to the corresponding compartments that is a critical role for cell viability and neurite outgrowth [45]. Furthermore, SNARE proteins seem to be important for *KRAS* regulation [46], where *KRAS* is a driver of many human malignancies, including NB [47]. Overexpression of *TSSC1* could lead to altered regulation of SNARE-mediated vesicle fusion, which could be associated with tumor progression. Since the transcription factor *MYCN* is known to regulate the NB microenvironment via secretion of exosomes or microvesicles, which increase cell proliferation [48], a functional link between *MYCN* and *TSSC1* genes is likely to exist. Our findings identified the *TSSC1* gene as an interesting candidate to be a putative new oncogene in high-risk NB and a possible objective for future targeted therapies. Other experimental data will be needed to confirm the activity of *TSSC1* as an oncogenic molecule in NB.

## 5. Conclusions

Among the protein-coding genes involved in the six co-amplifications on chromosome 2p found on an NB case with very poor outcome, the one most significantly associated with the worse survival of NB patients is *TSSC1* that, even in synergy with *MYCN*, could have a potential role in NB oncogenesis and cell cycle regulation. Extensive clinical and functional investigations are required to confirm the role of the *TSSC1* gene in NB.

## Figures and Tables

**Table 1:** Main characteristics of the datasets used to perform the analyses.

	Cangelosi et al. [33]		E-MTAB-1781		SEQC-498	
Patient Characteristics	N	%	N	%	N	%
Age at diagnosis						
<18 months	449	57.1	431	60.8	305	61.2
≥18 months	337	42.9	278	39.2	198	38.8
*MYCN* status						
Not amplified	629	80.0	581	82.0	401	80.5
Amplified	153	19.5	122	17.2	92	18.5
Missing	4	0.5	6	0.8	5	1.0
INSS Stage						
1	143	18.2	159	22.4	121	24.3
2	125	15.9	118	16.6	78	15.7
3	105	13.4	93	13.1	63	12.7
4	320	40.7	259	36.5	183	36.8
4s	92	11.7	80	11.3	53	10.6
Missing	1	0.1	0	0.0	0	0.0
Events	320	40.7 ^1^	256 ^2^	36.1	183	36.9
Deaths	229	29.1	161	22.7	105	21.1

^1^ Seventeen missing for the Event Free Survival. ^2^ Fourteen missing for the Event Free Survival.

**Table 2:** Overall Survival of 786 NB patients in relation to *RNF144A* expression levels evaluated by the Cox model.

		Univariable Analysis			Multivariable Analysis		
Gene Expression	N/D	HR	95% CI	*p*	HR	95% CI	*p*
Median				<0.001			0.022
≤4.544	393/151	1	ref		1	Ref	
>4.544	393/78	0.46	0.35–0.60		0.72	0.54–0.96	
Tertiles				<0.001			0.010
≤4.299	262/105	1	ref		1	ref	
4.299–4.759	262/81	0.73	0.55–0.98		1.0	0.75–1.4	
>4.759	262/43	0.36	0.25–0.51		0.61	0.42–0.88	
Continuous variable	786/229	0.55	0.45–0.66	<0.001	0.73	0.59–0.90	0.004

N/D = Number of patients/Deaths HR: Hazard Ratio. Multivariable analysis: HRs are adjusted by *MYCN* status, age, and stage at diagnosis.

**Table 3:** Event Free Survival of 769 NB patients in relation to *RNF144A* expression levels evaluated by the Cox model.

		Univariable Analysis			Multivariable Analysis		
Gene Expression	N/E	HR	95% CI	*p*	HR	95% CI	*p*
Median				<0.001			0.129
≤4.544	378/189	1	ref		1	ref	
>4.544	391/131	0.61	0.49–0.76		0.83	0.66–1.1	
Tertiles				<0.001			0.115
≤4.299	247/127	1	ref		1	ref	
4.299–4.759	260/112	0.83	0.64–1.1		1.1	0.82–1.4	
>4.759	262/81	0.54	0.41–0.71		0.79	0.59–1.1	
Continuous variable	769/320	0.62	0.52–0.75	<0.001	0.81	0.67–0.98	0.029

N/E = Number of patients/Events. Hazard Ratio. Multivariable analysis: HRs are adjusted by *MYCN* status, age, and stage at diagnosis.

**Table 4:** Overall Survival of 786 NB patients in relation to *MYT1L* expression levels evaluated by the Cox model.

		Univariable Analysis			Multivariable Analysis		
Gene Expression	N/D	HR	95% CI	*p*	HR	95% CI	*p*
Median				<0.001			0.004
≤4.316	393/171	1	ref		1	ref	
>4.316	393/58	0.28	0.21–0.38		0.64	0.46–0.87	
Tertiles				<0.001			0.006
≤3.991	262/128	1	ref		1	ref	
3.991–4.589	262/69	0.47	0.35–0.63		0.80	0.59–1.1	
>4.589	262/32	0.20	0.13–0.29		0.53	0.35–0.80	
Continuous variable	786/229	0.53	0.47–0.60	<0.001	0.73	0.62–0.86	<0.001

N/D = Number of patients/Deaths HR: Hazard Ratio. Multivariable analysis: HRs are adjusted by *MYCN* status, age, and stage at diagnosis.

**Table 5:** Event Free Survival of 769 NB patients in relation to *MYT1L* expression levels evaluated by the Cox model.

		Univariable Analysis			Multivariable Analysis		
Gene Expression	N/E	HR	95% CI	*p*	HR	95% CI	*p*
Median				<0.001			0.003
≤4.316	383/212	1	ref		1	ref	
>4.316	386/108	0.41	0.33–0.52		0.68	0.53–0.88	
Tertiles				<0.001			0.002
≤3.991	253/151	1	ref		1	ref	
3.991–4.589	257/107	0.64	0.50–0.82		0.90	0.70–1.2	
>4.589	259/62	0.31	0.23–0.42		0.57	0.41–0.79	
Continuous variable	769/320	0.59	0.52–0.66	<0.001	0.75	0.65–0.86	<0.001

N/E = Number of patients/Events. Hazard Ratio. Multivariable analysis: HRs are adjusted by *MYCN* status, age, and stage at diagnosis.

**Table 6:** Overall Survival of 786 NB patients in relation to *TSSC1* expression levels evaluated by the Cox model.

		Univariable Analysis			Multivariable Analysis		
Gene Expression	N/D	HR	95% CI	*p*	HR	95% CI	*p*
Median				<0.001			<0.001
≤3.346	393/75	1	ref		1	ref	
>3.346	393/154	2.4	1.8–3.1		1.7	1.3–2.2	
Tertiles				<0.001			0.002
≤3.162	262/43	1	ref		1	ref	
3.162–3.520	262/66	1.7	1.1–2.5		1.5	1.0–2.2	
>3.520	262/120	3.5	2.5–5.0		1.9	1.3–2.7	
Continuous variable	786/229	2.93	2.27–3.79	<0.001	1.48	1.16–1.90	0.002

N/D = Number of patients/Deaths HR: Hazard Ratio. Multivariable analysis: HRs are adjusted by *MYCN* status, age, and stage at diagnosis.

**Table 7:** Event Free Survival of 769 NB patients in relation to *TSSC1* expression levels evaluated by the Cox model.

		Univariable Analysis			Multivariable Analysis		
Gene Expression	N/E	HR	95% CI	*p*	HR	95% CI	*p*
Median				<0.001			0.178
≤1.877	383/138	1	ref		1	ref	
>1.877	386/182	1.5	1.2–1.8		1.2	0.93–1.5	
Tertiles				<0.001			0.376
≤3.162	256/89	1	ref		1	ref	
3.162–3.520	257/97	1.2	0.87–1.5		1.1	0.82–1.5	
>3.520	256/134	1.8	1.4–2.3		1.2	0.92–1.6	
Continuous variable	769/320	1.83	1.45–2.33	<0.001	1.2	0.96–1.5	0.103

N/E = Number of patients/Events. Hazard Ratio. Multivariable analysis: HRs are adjusted by *MYCN* status, age, and stage at diagnosis.

**Table 8:** Overall Survival of 498 NB patients in relation to *LOC730811* expression levels evaluated by the Cox model.

		Univariable Analysis			Multivariable Analysis		
Gene Expression	N/D	HR	95% CI	*p*	HR	95% CI	*p*
Median				0.005			0.820
≤0.535	248/40	1	ref		1	ref	
>0.535	250/65	1.7	1.2–2.6		1.0	0.69–1.6	
Tertiles				0.191			0.451
≤0.438	166/31	1	ref		1	ref	
0.438–0.638	166/32	1.0	0.63–1.7		0.74	0.44–1.2	
>0.638	166/42	1.5	0.92–2.3		0.93	0.57–1.5	
Continuous variable	498/105	3.07	1.67–5.7	<0.001	1.26	0.68–2.34	0.460

N/D = Number of patients/Deaths HR: Hazard Ratio. Multivariable analysis: HRs are adjusted by *MYCN* status, age, and stage at diagnosis.

**Table 9:** Event Free Survival of 498 NB patients in relation to *LOC730811* expression levels evaluated by the model.

		Univariable Analysis			Multivariable Analysis		
Gene Expression	N/E	HR	95% CI	*p*	HR	95% CI	*p*
Median				0.073			0.977
≤0.535	248/82	1	ref		1	ref	
>0.535	250/101	1.3	0.97–1.7		1.0	0.73–1.4	
Tertiles				0.883			0.358
≤0.438	166/63	1	ref		1	ref	
0.438–0.638	166/60	0.92	0.64–1.3		0.79	0.55–1.1	
>0.638	166/60	0.97	0.68–1.4		0.79	0.54–1.1	
Continuous variable	498/183	1.44	0.86–2.40	0.173	0.91	0.54–1.52	0.723

N/E = Number of patients/Events. HR: Hazard Ratio. Multivariable analysis: HRs are adjusted by *MYCN* status, age, and stage at diagnosis.

**Table 10:** Overall Survival of 786 NB patients in relation to *CMPK2* expression levels evaluated by the Cox model.

		Univariable Analysis			Multivariable Analysis		
Gene Expression	N/D	HR	95% CI	*p*	HR	95% CI	*p*
Median				0.020			0.498
≤1.877	393/99	1	ref		1	ref	
>1.877	393/130	1.4	1.0–1.8		0.91	0.70–1.2	
Tertiles				0.132			0.612
≤1.541	262/66	1	ref		1	ref	
1.541–2.250	262/74	1.1	0.81–1.56		1.0	0.73–1.4	
>2.250	262/89	1.4	1.0–1.9		0.88	0.64–1.2	
Continuous variable	786/229	1.08	0.96–1.23	0.194	0.99	0.86–1.12	0.827

N/D = Number of patients/Deaths HR: Hazard Ratio. Multivariable analysis: HRs are adjusted by *MYCN* status, age, and stage at diagnosis.

**Table 11:** Event Free Survival of 769 NB patients in relation to *CMPK2* expression levels evaluated by the Cox model.

		Univariable Analysis			Multivariable Analysis		
Gene Expression	N/E	HR	95% CI	*p*	HR	95% CI	*p*
Median				0.015			0.910
≤1.877	387/141	1	ref		1	Ref	
>1.877	382/179	1.3	1.1–1.6		0.99	0.79–1.2	
Tertiles				0.125			0.759
≤1.541	258/94	1	ref		1	ref	
1.541–2.250	258/105	1.1	0.85–1.5		1.1	0.80–1.4	
>2.250	253/121	1.3	1.0–1.7		0.96	0.73–1.3	
Continuous variable	769/320	1.09	0.98–1.21	0.120	1.01	0.91–1.12	0.840

N/E = Number of patients/Events HR. Hazard Ratio. Multivariable analysis: HRs are adjusted by *MYCN* status, age, and stage at diagnosis.

**Table 12:** Overall Survival of 786 NB patients in relation to *RSAD2* expression levels evaluated by the Cox model.

		Univariable Analysis			Multivariable Analysis		
Gene Expression	N/D	HR	95% CI	*p*	HR	95% CI	*p*
Median				0.510			0.517
≤1.655	393/110	1	ref		1	ref	
>1.655	393/119	1.1	0.84–1.4		0.92	0.71–1.2	
Tertiles				0.021			0.529
≤1.193	262/80	1	ref		1	ref	
1.193–2.144	262/61	0.71	0.51–0.99		0.82	0.59–1.2	
>2.144	262/88	1.1	0.81–1.5		0.90	0.66–1.2	
Continuous variable	786/229	0.95	0.86–1.0	0.266	0.94	0.85–1.03	0.176

N/D = Number of patients/Deaths HR: Hazard Ratio. Multivariable analysis: HRs are adjusted by MYCN status, age, and stage at diagnosis.

**Table 13:** Event Free Survival of 769 NB patients in relation to RSAD2 expression levels evaluated by the Cox model.

		Univariable Analysis			Multivariable Analysis		
Gene Expression	N/E	HR	95% CI	*p*	HR	95% CI	*p*
Median				0.925			0.194
≤1.655	382/157	1	ref		1	ref	
>1.655	387/163	0.99	0.79–1.2		0.86	0.69–1.1	
Tertiles				0.024			0.188
≤1.193	253/110	1	ref		1	ref	
1.193–2.144	257/91	0.72	0.55–0.95		0.77	0.58–1.0	
>2.144	259/119	1.0	0.78–1.3		0.84	0.65–1.1	
Continuous variable	769/320	0.94	0.86–1.03	0.223	0.93	0.85–1.02	0.123

N/E = Number of patients/Events HR. Hazard Ratio. Multivariable analysis: HRs are adjusted by MYCN status, age, and stage at diagnosis.

**Table 14:** Association between *LOC339788* expression levels and the main patient characteristics at diagnosis.

	*LOC339788* Expression Levels				
	≤0.01436		>0.01436		
Patient Characteristics	N	%	N	%	*p*
Age					0.898
<18 months	226	61.1	79	61.7	
≥18 months	144	38.9	49	38.3	
MYCN status					0.024
Not amplified	307	83.7	94	74.6	
Amplified	60	16.4	32	25.4	
Stage					0.498
Localized	195	52.7	67	52.3	
Stage 4	139	37.6	44	34.4	
Stage 4S	36	9.7	17	13.3	

**Table 15:** Overall Survival of 498 NB patients in relation to *LOC339788* expression levels evaluated by the Cox model.

		Univariable Analysis			Multivariable Analysis		
Gene Expression	N/D	HR	95% CI	*p*	HR	95% CI	*p*
Cut-off				0.607			0.921
≤0.01436	370/76	1	ref		1	ref	
>0.01436	128/29	1.1	0.73–1.7		0.98	0.63–1.5	

N/D = Number of patients/Deaths. HR: Hazard Ratio. Multivariable analysis: HRs are adjusted by MYCN status, age, and stage at diagnosis. Cut-off: cut-off corresponding to the baseline expression value.

**Table 16:** Event Free Survival of 498 NB patients in relation to *LOC339788* expression levels evaluated by the Cox model.

		Univariable Analysis			Multivariable Analysis		
Gene Expression	N/E	HR	95% CI	*p*	HR	95% CI	*p*
Cut-off				0.261			0.471
≤0.01436	370/131	1	ref		1	ref	
>0.01436	128/52	1.2	0.87–1.7		1.1	0.81–1.6	

N/E = Number of patients/Events HR. HR: Hazard Ratio. Multivariable analysis: HRs are adjusted by *MYCN* status, age, and stage at diagnosis. Cut-off = cut-off corresponding to the baseline expression value.

**Table 17:** Overall Survival of 786 NB patients in relation to *GREB1* expression levels evaluated by the Cox model.

		Univariable Analysis			Multivariable Analysis		
Gene Expression	N/D	HR	95% CI	*p*	HR	95% CI	*p*
Median				0.811			0.738
≤4.029	393/114	1	ref		1	ref	
>4.029	393/115	1.0	0.80–1.3		0.95	0.73–1.3	
Tertiles				0.058			0.367
≤3.791	262/89	1	ref		1	ref	
3.791–4.311	262/62	0.68	0.49–0.94		0.80	0.58–1.1	
>4.311	262/78	0.88	0.65–1.2		0.84	0.61–1.2	
Continuous variable	786/229	0.97	0.83–1.1	0.672	0.89	0.78–1.0	0.118

N/D = Number of patients/Deaths HR: Hazard Ratio. Multivariable analysis: HRs are adjusted by *MYCN* status, age, and stage at diagnosis.

**Table 18:** Event Free Survival of 769 NB patients in relation to *GREB1* expression levels evaluated by the Cox regression model.

		Univariable Analysis			Multivariable Analysis		
Gene Expression	N/E	HR	95% CI	*p*	HR	95% CI	*p*
Median				0.398			0.265
≤4.029	384/167	1	ref		1	ref	
>4.029	385/153	0.91	0.73–1.1		0.88	0.70–1.1	
Tertiles				0.007			0.113
≤3.791	253/127	1	ref		1	ref	
3.791–4.311	260/91	0.65	0.50–0.86		0.77	0.58–1.0	
>4.311	256/102	0.78	0.60–1.0		0.79	0.60–1.0	
Continuous variable	769/320	0.87	0.77–0.99	0.039	0.86	0.76–0.97	0.014

N/E = Number of patients/Events HR: Hazard Ratio. Multivariable analysis: HRs are adjusted by *MYCN* status, age, and stage at diagnosis.

**Table 19:** Overall Survival of 709 NB patients in relation to *NTSR2* expression levels evaluated by the Cox regression model.

		Univariable Analysis			Multivariable Analysis		
Gene Expression	N/D	HR	95% CI	*p*	HR	95% CI	*p*
Median				0.033			0.192
≤−0.944	354/90	1	ref		1	ref	
>−0.944	355/71	0.71	0.52–0.97		0.81	0.59–1.1	
Tertiles				0.066			0.578
≤−1.303	236/62	1	ref		1	ref	
−1.303–−0.594	236/44	0.63	0.43–0.93		0.86	0.58–1.3	
>−0.594	237/55	0.79	0.55–1.1		0.83	0.57–1.2	
Continuous variable	709/161	0.92	0.85–0.99	0.036	0.96	0.90–1.0	0.199

N/D = Number of patients/Deaths HR: Hazard Ratio. Multivariable analysis: HRs are adjusted by *MYCN* status, age, and stage at diagnosis.

**Table 20:** Event Free Survival of 695 NB patients in relation to *NTSR2* expression levels evaluated by the Cox regression model.

		Univariable Analysis			Multivariable Analysis		
Gene Expression	N/E	HR	95% CI	*p*	HR	95% CI	*p*
Median				0.066			0.131
≤−0.944	349/138	1	ref		1	ref	
>−0.944	346/118	0.79	0.62–1.0		0.82	0.64–1.1	
Tertiles				0.088			0.166
≤−1.303	231/96	1	ref		1	ref	
−1.303–−0.594	235/79	0.75	0.55–1.0		0.90	0.66–1.2	
>−0.594	229/81	0.75	0.56–1.0		0.75	0.55–1.0	
Continuous variable	695/256	0.95	0.89–1.0	0.105	0.97	0.91–1.0	0.244

N/E = Number of patients/Events HR: Hazard Ratio. Multivariable analysis: HRs are adjusted by *MYCN* status, age, and stage at diagnosis.

**Table 21:** Overall Survival of 786 NB patients in relation to *LPIN1* expression levels evaluated by the Cox regression model.

		Univariable Analysis			Multivariable Analysis		
Gene Expression	N/D	HR	95% CI	*p*	HR	95% CI	*p*
Median				<0.001			0.075
≤4.313	393/149	1	ref		1	ref	
>4.313	393/80	0.49	0.37–0.64		0.78	0.59–1.0	
Tertiles				<0.001			0.004
≤4.102	262/113	1	ref		1	ref	
4.102–4.512	262/67	0.55	0.40–0.74		1.0	0.75–1.4	
>4.512	262/49	0.38	0.27–0.53		0.60	0.42–0.84	
Continuous variable	786/229	0.59	0.48–0.73	<0.001	0.75	0.61–0.91	0.003

N/D = Number of patients/Deaths HR: Hazard Ratio. Multivariable analysis: HRs are adjusted by *MYCN* status, age, and stage at diagnosis.

**Table 22:** Event Free Survival of 769 NB patients in relation to *LPIN1* expression levels evaluated by the Cox regression model.

		Univariable Analysis			Multivariable Analysis		
Gene Expression	N/E	HR	95% CI	*p*	HR	95% CI	*p*
Median				<0.001			0.064
≤4.313	376/189	1	ref		1	ref	
>4.313	393/131	0.60	0.48–0.75		0.81	0.64–1.0	
Tertiles				<0.001			0.060
≤4.102	245/135	1	ref		1	ref	
4.102–4.512	262/98	0.62	0.48–0.81		0.94	0.72–1.2	
>4.512	262/87	0.53	0.40–0.69		0.73	0.55–0.96	
Continuous variable	769/320	0.62	0.50–0.76	<0.001	0.77	0.64–0.92	0.004

N/E = Number of patients/Events HR: Hazard Ratio. Multivariable analysis: HRs are adjusted by *MYCN* status, age, and stage at diagnosis.

**Table 23:** Overall Survival of 786 NB patients in relation to *NBAS* expression levels evaluated by the Cox regression model.

		Univariable Analysis			Multivariable Analysis		
Gene Expression	N/D	HR	95% CI	*p*	HR	95% CI	*p*
Median				0.010			0.153
≤3.311	393/99	1	ref		1	ref	
>3.311	393/130	1.4	1.1–1.8		1.2	0.93–1.6	
Tertiles				<0.001			0.301
≤3.074	261/71	1	ref		1	ref	
3.074–3.585	263/62	0.83	0.59–1.2		1.3	0.90–1.8	
>3.585	262/96	1.5	1.1–2.0		1.2	0.90–1.7	
Continuous variable	786/229	1.26	1.16–1.37	<0.001	0.96	0.89–1.03	0.231

N/D = Number of patients/Deaths HR: Hazard Ratio. Multivariable analysis: HRs are adjusted by *MYCN* status, age, and stage at diagnosis.

**Table 24:** Event Free Survival of 769 NB patients in relation to *NBAS* expression levels evaluated by the Cox regression model.

		Univariable Analysis			Multivariable Analysis		
Gene Expression	N/E	HR	95% CI	*p*	HR	95% CI	*p*
Median				0.063			0.340
≤3.311	376/146	1	ref		1	ref	
>3.311	393/174	1.2	0.99–1.5		1.1	0.89–1.4	
Tertiles				0.008			0.183
≤3.074	244/95	1	ref		1	ref	
3.074–3.585	263/99	0.99	0.74–1.3		1.3	0.95–1.7	
>3.585	262/126	1.4	1.1–1.9		1.2	0.95–1.6	
Continuous variable	769/320	1.17	1.09–1.26	<0.001	0.98	0.91–1.05	0.574

N/E = Number of patients/Events HR: Hazard Ratio. Multivariable analysis: HRs are adjusted by *MYCN* status, age, and stage at diagnosis.

**Table 25:** Diagnostic characteristics of stage 4 NB patients whose tumors were analyzed by immunofluorescence for TSSC1.

Case N°	Age at Onset (Months)	INSS Stage	INRG Stage	Subtype	*MYCN* Status	Percentage of TSSC1 Positive Cells	Relapse	Follow-Up	Disease State
1	35	4	M	NB/GNBL	gain	85	yes	dead	
2	15	4	M	NB/GNBL	amp	70	yes	dead	
3	49	4	M	NB/GNBL	amp	73	yes	dead	
4	36	4	M	NB/GNBL	sc	20	no	alive	CR
5	12	4	M	NB/GNBL	sc	15	no	alive	CR
6	11	4	M	NB/GNBL	amp	67	yes	dead	
7	18	4	M	NB/GNBL	amp	75	yes	dead	
8	22	4	M	NB/GNBL	amp	68	yes	dead	
9	7	4	M	NB/GNBL	amp	78	no	dead	
10	3	4	M	NB/GNBL	sc	12	no	alive	CR
11	47	4	M	NB/GNBL	gain	54	yes	dead	
12	9	4	M	NB/GNBL	amp	9	no	alive	CR
13	26	4	M	NB/GNBL	amp	62	yes	dead	
14	63	4	M	NB NAS	amp	78	yes	dead	
15	46	4	M	NB/GNBL	amp	64	yes	dead	
16	165	4	M	NB/GNBL	sc	11	no	alive	CR
17	114	4	M	NB/GNBL	amp	72	yes	dead	
18	23	4	M	NB/GNBL	sc	7	no	alive	CR
19	33	4	M	NB/GNBL	sc	13	no	alive	CR
20	32	4	M	NB/GNBL	gain	45	yes	dead	
21	45	4	M	NB/GNBL	gain	59	yes	dead	
22	28	4	M	NB/GNBL	gain	63	yes	dead	
23	57	4	M	NB/GNBL	amp	54	no	alive	AD
24	7	4	M	NB/GNBL	sc	6	no	alive	CR
25	28	4	M	NB/GNBL	gain	77	yes	dead	
26	54	4	M	NB/GNBL	amp	55	yes	dead	
27	55	4	M	NB/GNBL	amp	59	no	dead	
28	52	4	M	NB/GNBL	gain	62	yes	dead	
29	5	4	M	NB/GNBL	amp	4	no	alive	CR
30	15	4	M	NB/GNBL	amp	81	yes	dead	

Abbreviations: AD, active disease; amp, amplified; CR, complete remission; GNBL, ganglioneuroblastoma; INSS, International Neuroblastoma Staging System; IRNG, International Neuroblastoma Risk Group; NB, neuroblastoma; NAS, neuroblastoma abdominal mass; sc, single copy.

## Data Availability

Data are contained within the article or Supplementary Material.

## Supplementary Materials

The following are available online at https://www.mdpi.com/article/10.3390/cancers13225792/s1, Figure S1: Association between *RNF144A* gene expression and the main patient characteristics at diagnosis and outcome evaluated on the entire cohort. Figure S2: Survival of patients with age ≥ 18 months, stage 4 disease and *MYCN* amplification at diagnosis in relation to *RNF144A* expression level. Figure S3: Association between *MYT1L* gene expression and the main patient characteristics at diagnosis and outcome evaluated on the entire cohort. Figure S4: Survival of patients with age ≥ 18 months, stage 4 disease and *MYCN* amplification at diagnosis in relation to *MYT1L* expression level. Figure S5: Association between *TSSC1* gene expression and the main patient characteristics at diagnosis and outcome evaluated on the entire cohort. Figure S6: Survival of patients with age ≥ 18 months, stage 4 disease and *MYCN* amplification at diagnosis in relation to *TSSC1* expression level. Figure S7: Association between *LOC730811* expression and the main patient characteristics at diagnosis and outcome evaluated on the entire cohort. Figure S8: Survival of patients with age ≥ 18 months, stage 4 disease and *MYCN* amplification at diagnosis in relation to *LOC730811* expression level. Figure S9: Association between *CMPK2* gene expression and the main patient characteristics at diagnosis and outcome evaluated on the entire cohort. Figure S10: Survival of patients with age ≥ 18 months, stage 4 disease and *MYCN* amplification at diagnosis in relation to *CMPK2* expression level. Figure S11: Association between *RSAD2* gene expression and the main patient characteristics at diagnosis and outcome evaluated on the entire cohort. Figure S12: Survival of patients with age ≥ 18 months, stage 4 disease and *MYCN* amplification at diagnosis in relation to *RSAD2* expression level. Figure S13: Association between *GREB1* gene expression and the main patient characteristics at diagnosis and outcome evaluated on the entire cohort. Figure S14: Survival of patients with age ≥ 18 months, stage 4 disease and *MYCN* amplification at diagnosis in relation to *GREB1* expression level. Figure S15: Association between *NTSR2* gene expression and the main patient characteristics at diagnosis and outcome evaluated on the entire cohort. Figure S16: Survival of patients with age ≥ 18 months, stage 4 disease and *MYCN* amplification at diagnosis in relation to *NTSR2* expression level. Figure S17: Association between *LPIN1* gene expression and the main patient characteristics at diagnosis and outcome evaluated on the entire cohort. Figure S18: Survival of patients with age ≥ 18 months, stage 4 disease and *MYCN* amplification at diagnosis in relation to *LPIN1* expression level. Figure S19: Association between *NBAS* gene expression and the main patient characteristics at diagnosis and outcome evaluated on the entire cohort. Figure S20: Survival of patients with age ≥ 18 months, stage 4 disease and *MYCN* amplification at diagnosis in relation to *NBAS* expression level. Figure S21: Relative *TSSC1* expression levels were plotted in not relapsed or relapsed patients and in alive or dead patients, Table S1: Expression level of the target genes in the normal adrenal gland tissue, Table S2: Overall Survival of 786 NB patients in relation to *RNF144A* expression levels by *MYCN* status, Table S3: Overall Survival of 786 NB patients in relation to *RNF144A* expression levels by stage at diagnosis, evaluated by the Kaplan–Meier method, Table S4: Event Free Survival of 769 NB patients in relation to *RNF144A* expression levels by *MYCN* status, Table S5: Event Free Survival of 769 NB patients in relation to *RNF144A* expression levels by stage at diagnosis, evaluated by the Kaplan–Meier method, Table S6: Overall Survival of 786 NB patients in relation to *MYT1L* expression levels by *MYCN* status, Table S7: Overall Survival of 786 NB patients in relation to *MYT1L* expression levels by stage at diagnosis, evaluated by the Kaplan–Meier method, Table S8: Event Free Survival of 769 NB patients in relation to *MYT1L* expression levels by *MYCN* status, Table S9: Event Free Survival of 769 NB patients in relation to *MYT1L* expression levels by stage at diagnosis, evaluated by the Kaplan–Meier method, Table S10: Overall Survival of 786 NB patients in relation to *TSSC1* expression levels by *MYCN* status, Table S11: Overall Survival of 786 NB patients in relation to *TSSC1* expression levels by stage at diagnosis, evaluated by the Kaplan–Meier method, Table S12: Event Free Survival of 786 NB patients in relation to *TSSC1* expression levels by *MYCN* status, Table S13: Event Free Survival of 769 NB patients in relation to *TSSC1* expression levels by stage at diagnosis, evaluated by the Kaplan–Meier method, Table S14: Overall Survival of 498 NB patients in relation to *LOC730811* expression levels by *MYCN* status, Table S15: Overall Survival of 498 NB patients in relation to *LOC730811* expression levels by stage at diagnosis, evaluated by the Kaplan–Meier method, Table S16: Event Free Survival of 498 NB patients in relation to *LOC730811* expression levels by *MYCN* status, Table S17: Event Free Survival of 498 NB patients in relation to *LOC730811* expression levels by stage at diagnosis, evaluated by the Kaplan–Meier method, Table S18: Overall Survival of 786 NB patients in relation to *CMPK2* expression levels by *MYCN* status, Table S19: Overall Survival of 786 NB patients in relation to *CMPK2* expression levels by stage at diagnosis, evaluated by the Kaplan–Meier method, Table S20: Event Free Survival of 769 NB patients in relation to *CMPK2* expression levels by *MYCN* status, Table S21: Event Free Survival of 769 NB patients in relation to *CMPK2* expression levels by stage at diagnosis, evaluated by the Kaplan–Meier method, Table S22: Overall Survival of 786 NB patients in relation to *RSAD2* expression levels by *MYCN* status, Table S23: Overall Survival of 786 NB patients in relation to *RSAD2* expression levels by stage at diagnosis, evaluated by the Kaplan–Meier method, Table S24: Event Free Survival of 769 NB patients in relation to *RSAD2* expression levels by *MYCN* status, Table S25: Event Free Survival of 769 NB patients in relation to *RSAD2* expression levels by stage at diagnosis, evaluated by the Kaplan–Meier method, Table S26: Association between *LOC339788* expression levels by outcome and main patient characteristics at diagnosis. Table S27: Overall Survival of 498 NB patients in relation to *LOC339788* expression levels by *MYCN* status, Table S28: Overall Survival of 498 NB patients in relation to *LOC339788* expression levels by stage at diagnosis, evaluated by the Kaplan–Meier method, Table S29: Event Free Survival of 498 NB patients in relation to *LOC339788* expression levels by *MYCN* status, Table S30: Event Free Survival of 498 NB patients in relation to *LOC339788* expression levels by stage at diagnosis, evaluated by the Kaplan–Meier method, Table S31: Overall Survival of 786 NB patients in relation to *GREB1* expression levels by *MYCN* status, Table S32: Overall Survival of 786 NB patients in relation to *GREB1* expression levels by stage at diagnosis, evaluated by the Kaplan–Meier method, Table S33: Event Free Survival of 769 NB patients in relation to *GREB1* expression levels by *MYCN* status, Table S34: Event Free Survival of 769 NB patients in relation to *GREB1* expression levels by stage at diagnosis, evaluated by the Kaplan–Meier method, Table S35: Overall Survival of 709 NB patients in relation to *NTSR2* expression levels by *MYCN* status, Table S36: Overall Survival of 709 NB patients in relation to *NTSR2* expression levels by stage at diagnosis, evaluated by the Kaplan–Meier method, Table S37: Event Free Survival of 695 NB patients in relation to *NTSR2* expression levels by *MYCN* status, Table S38: Event Free Survival of 695 NB patients in relation to *NTSR2* expression levels by stage at diagnosis, evaluated by the Kaplan–Meier method, Table S39: Overall Survival of 786 NB patients in relation to *LPIN1* expression levels by *MYCN* status, Table S40: Overall Survival of 786 NB patients in relation to *LPIN1* expression levels by stage at diagnosis, evaluated by the Kaplan–Meier method, Table S41: Event Free Survival of 769 NB patients in relation to *LPIN1* expression levels by *MYCN* status, Table S42: Event Free Survival of 769 NB patients in relation to *LPIN1* expression levels by stage at diagnosis, evaluated by the Kaplan–Meier method, Table S43: Overall Survival of 786 NB patients in relation to *NBAS* expression levels by *MYCN* status, Table S44: Overall Survival of 786 NB patients in relation to *NBAS* expression levels by stage at diagnosis, evaluated by the Kaplan–Meier method, Table S45: Event Free Survival of 769 NB patients in relation to *NBAS* expression levels by *MYCN* status, Table S46: Event Free Survival of 769 NB patients in relation to *NBAS* expression levels by stage at diagnosis, evaluated by the Kaplan–Meier method.
