# Supplementary material for: High Grade of Amplification of Six Regions on Chromosome 2p in a Neuroblastoma Patient with Very Poor Outcome: The Putative New Oncogene TSSC1
Source: Cancers (Basel). 2021 Nov 18;13(22):5792. doi: 10.3390/cancers13225792 (PMC8616235; doi:10.3390/cancers13225792)
Supplement: Supplementary file 1 [file cancers-13-05792-s001.zip › cancers-1414318-supplementary.pdf]

Supplementary Figures

Supplementary Figure S1

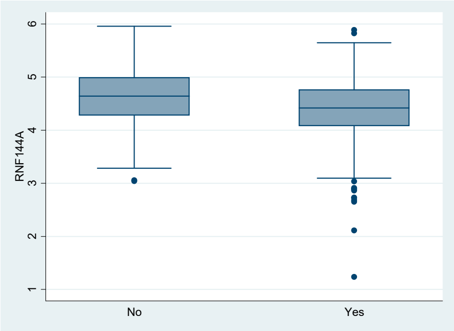

(a) p < 0.001

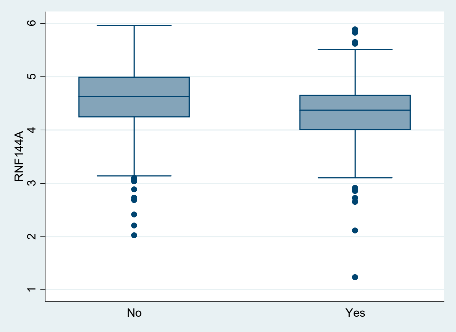

(b) p < 0.001

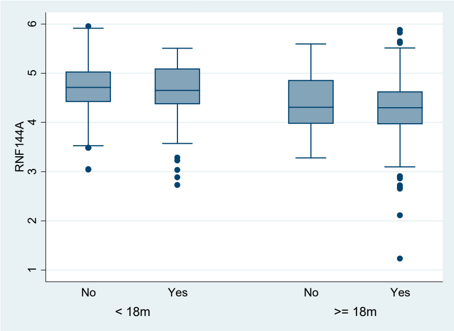

(c) p = 0.348; p = 0.471

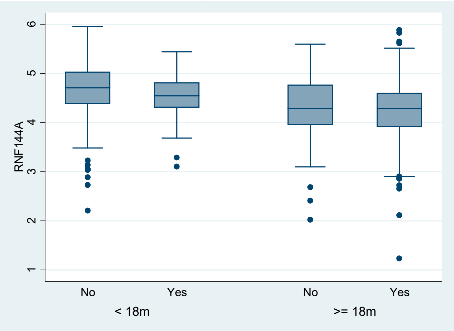

(d) p = 0.083; p = 0.296

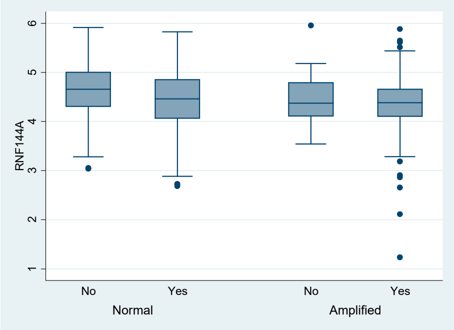

(e) p < 0.001; p = 0.804

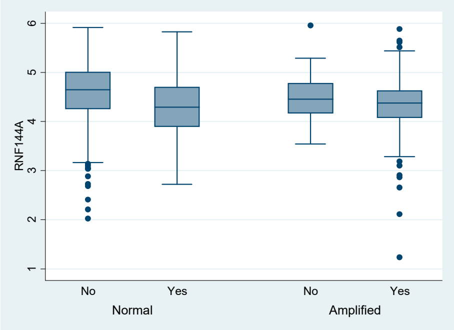

(f) p < 0.001; p = 0.188

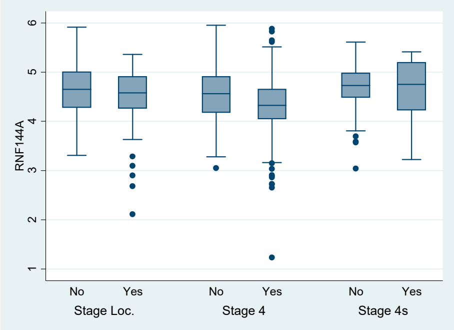

(g) p = 0.282; p < 0.001; p = 0.864

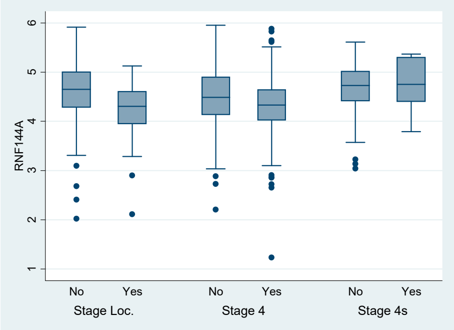

(h) p < 0.001; p = 0.005; p = 0.862

Association between *RNF144A* gene expression and the main patient characteristics at diagnosis and outcome evaluated on the entire cohort. **(a)** Number of events. **(b)** Number of deaths. **(c)** Number of events by age at diagnosis. **(d)** Number of deaths by age at diagnosis. **(e)** Number of events by *MYCN* status. **(f)** Number of deaths by *MYCN* status. **g)** Number of events by stage at diagnosis. **(f)** Number of deaths by stage at diagnosis.

Supplementary Figure S2

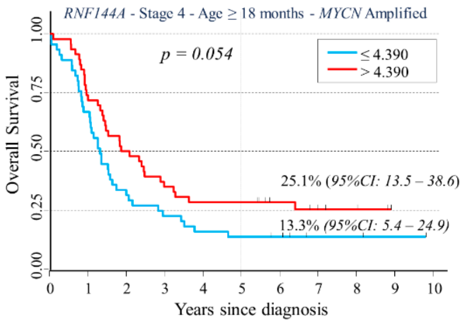

(**A**)

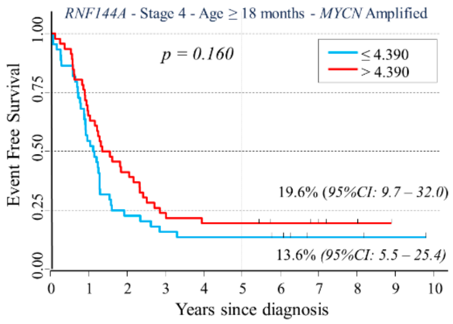

(**B**)

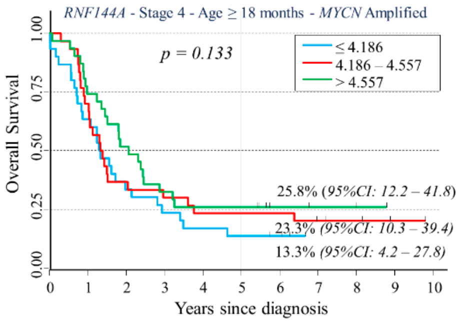

(**C**)

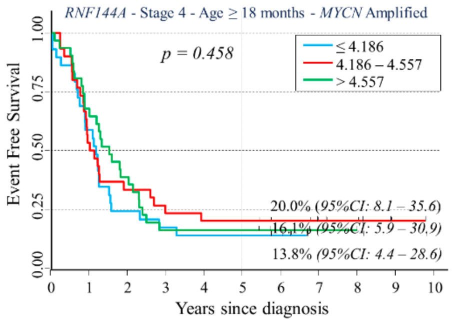

(**D**)

Survival of patients with age ≥ 18 months, stage 4 disease and *MYCN* amplification at diagnosis in relation to *RNF144A* expression level. (**A**) Overall Survival, cut-off based on the median expression value. (**B**) Event Free Survival, cut-off based on the median expression value. (**C**) Overall Survival, cut-offs based on tertile expression values. (**D**) Event Free Survival, cut-offs based on tertile expression values. Five-year survival estimates are displayed.

Supplementary Figure S3

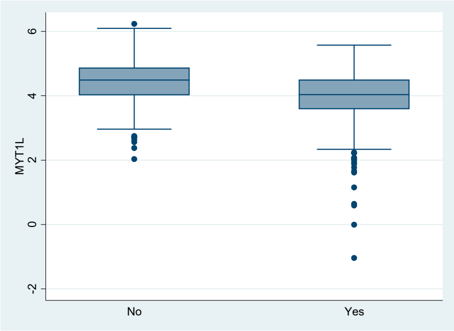

(**a**) p < 0.001

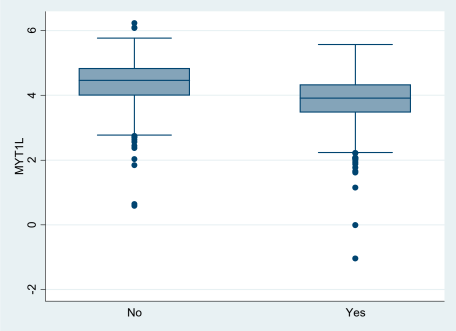

(**b**) p < 0.001

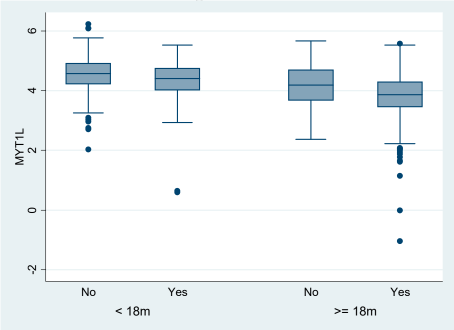

(c) p = 0.002; p < 0.001

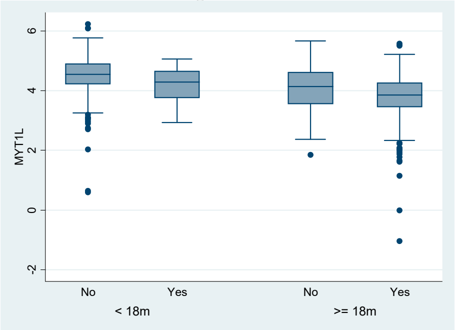

(d) p = 0.001; p < 0.001

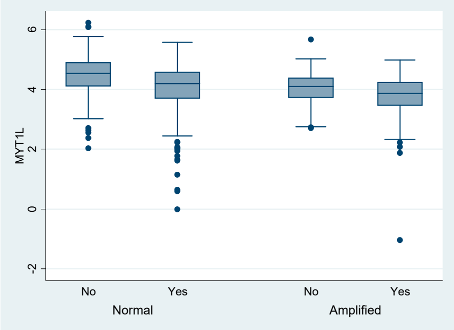

(e) p < 0.001; p = 0.004

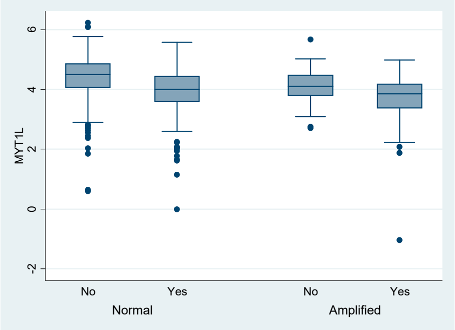

(f) p < 0.001; p = 0.007

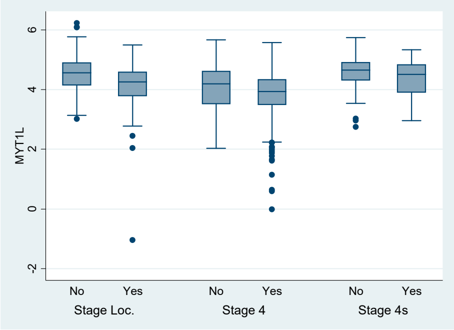

(g) p < 0.001; p = 0.007; p = 0.251

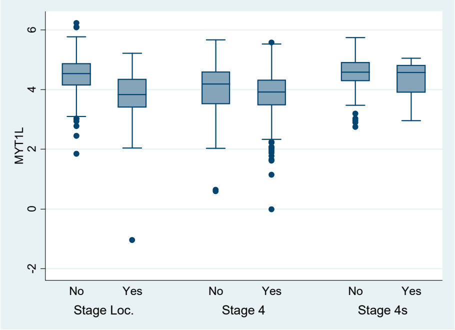

(h) p < 0.001; p = 0.003; p = 0.431

Association between *MYT1L* gene expression and the main patient characteristics at diagnosis and outcome evaluated on the entire cohort. **(a)** Number of events. **(b)** Number of deaths. **(c)** Number of events by age at diagnosis. **(d)** Number of deaths by age at diagnosis. **(e)** Number of events by *MYCN* status. **(f)** Number of deaths by *MYCN* status. **g)** Number of events by stage at diagnosis. **(f)** Number of deaths by stage at diagnosis.

Supplementary Figure S4

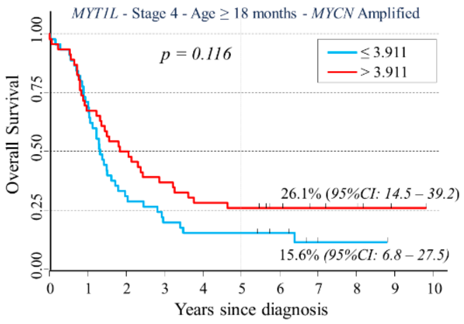

(**A**)

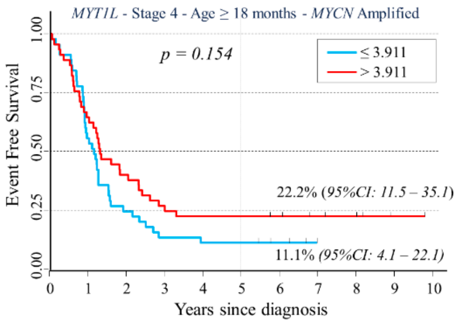

(**B**)

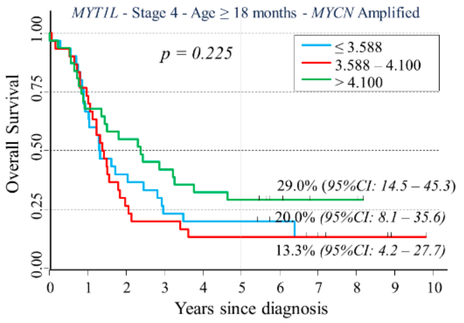

(**C**)

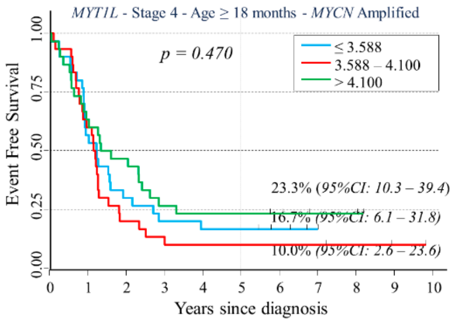

(**D**) p = 0.470

Survival of patients with age ≥ 18 months, stage 4 disease and *MYCN* amplification at diagnosis in relation to *MYT1L* expression level. (**A**) Overall Survival, cut-off based on the median expression value. (**B**) Event Free Survival, cut-off based on the median expression value. (**C**) Overall Survival, cut-offs based on tertile expression values. (**D**) Event Free Survival, cut-offs based on tertile expression values. Five-year survival estimates are displayed.

Supplementary Figure S5

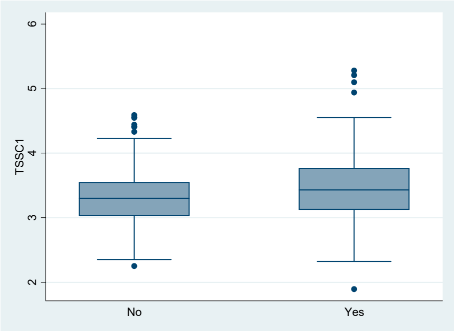

(**a**) p < 0.001

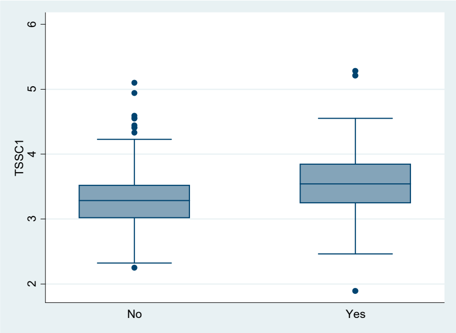

(**b**) p < 0.001

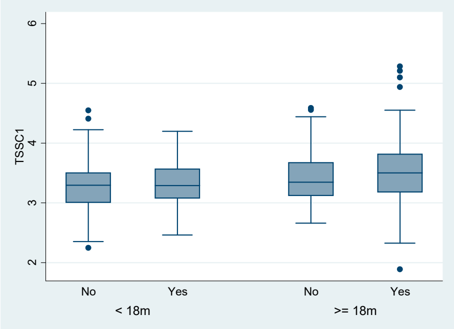

(**c**) p = 0.206; p = 0.035

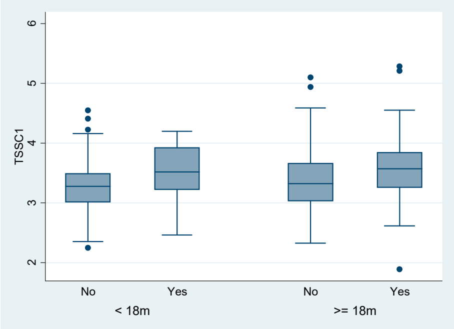

(**d**) p < 0.001; p < 0.001

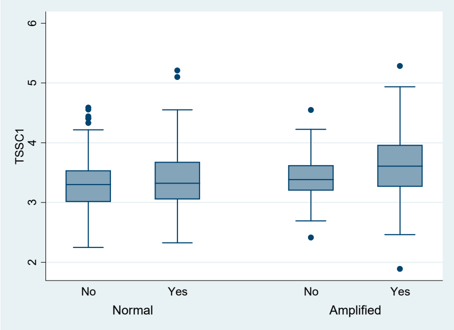

(**e**) p = 0.089; p = 0.018

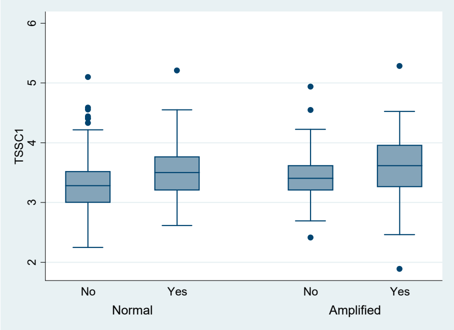

(**f**) p < 0.001; p = 0.028

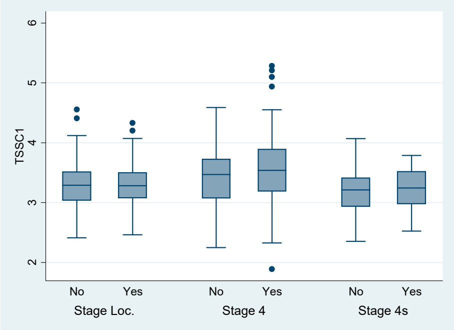

(**g**) p = 0.652; p = 0.070; p = 0.495

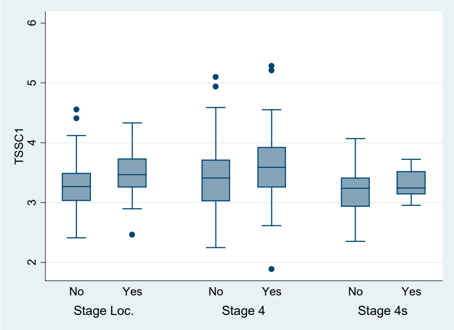

(**h**) p < 0.001; p = 0.001; p = 0.315

Association between *TSSC1* gene expression and the main patient characteristics at diagnosis and outcome evaluated on the entire cohort. **(a)** Number of events. **(b)** Number of deaths. **(c)** Number of events by age at diagnosis. **(d)** Number of deaths by age at diagnosis. **(e)** Number of events by *MYCN* status. **(f)** Number of deaths by *MYCN* status. **g)** Number of events by stage at diagnosis. **(f)** Number of deaths by stage at diagnosis.

Supplementary Figure S6

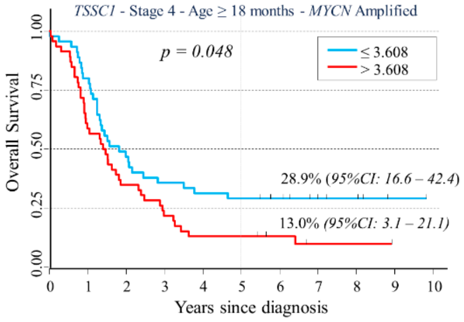

(**A**)

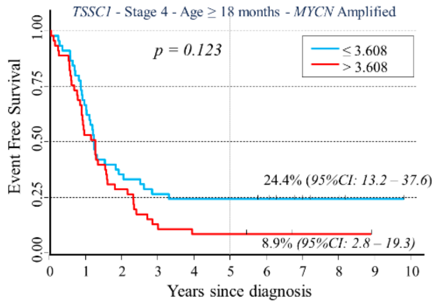

(**B**)

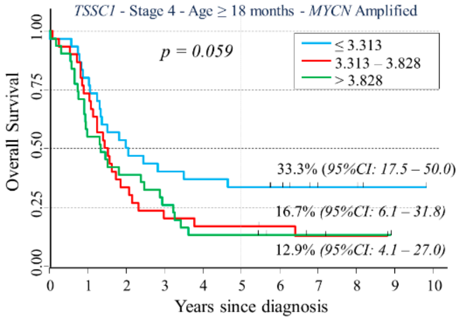

(**C**)

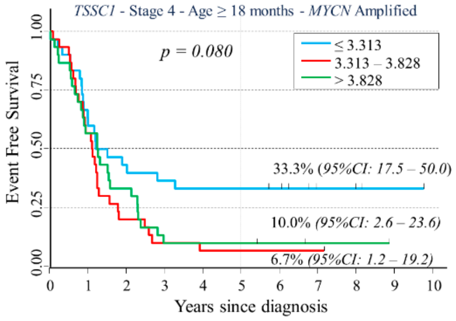

(**D**)

Survival of patients with age ≥ 18 months, stage 4 disease and *MYCN* amplification at diagnosis in relation to *TSSC1* expression level. (**A**) Overall Survival, cut-off based on the median expression value. (**B**) Event Free Survival, cut-off based on the median expression value. (**C**) Overall Survival, cut-offs based on tertile expression values. (**D**) Event Free Survival, cut-offs based on tertile expression values. Five-year survival estimates are displayed.

Supplementary Figure S7

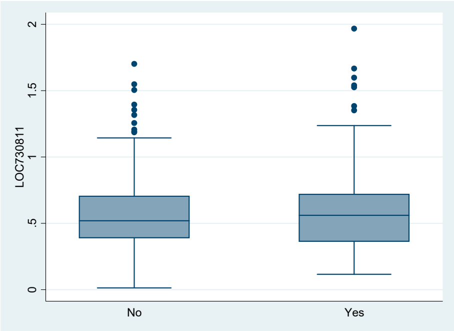

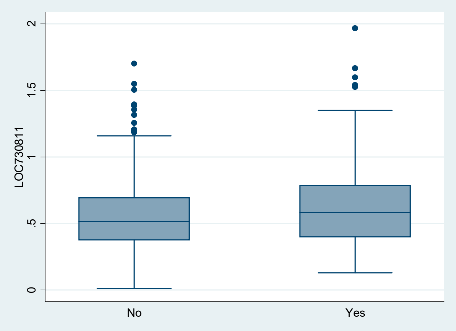

(**a**) p = 0.715 (**b**) p = 0.020

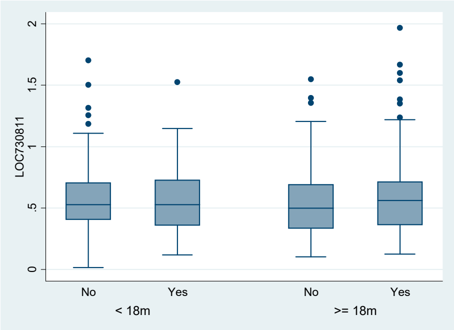

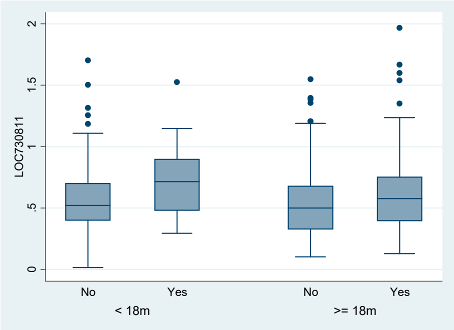

(**c**) p = 0.969; p = 0.446 (**d**) p = 0.017; p = 0.063

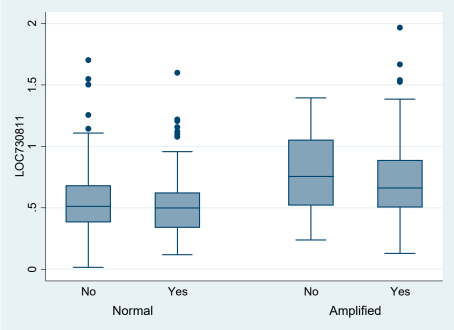

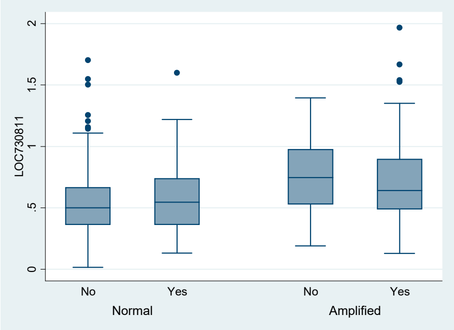

(**e**) p = 0.205; p = 0.539 (**f**) p = 0.611; p = 0.574

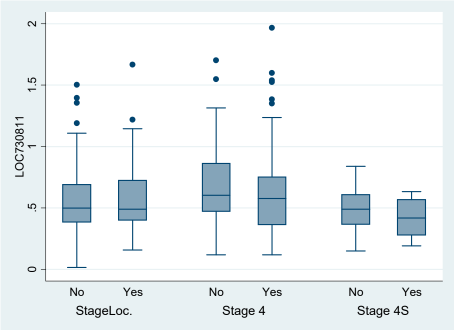

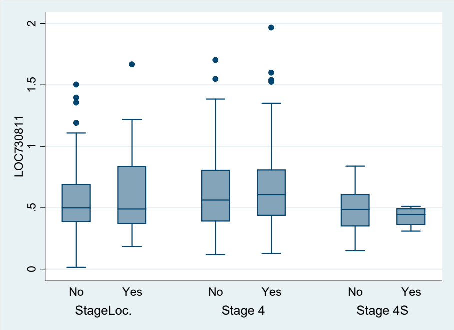

(**g**) p = 0.920; p = 0.283; p = 0.211 (**h**) p = 0.765; p = 0.283; p = 0.429

Association between *LOC730811* expression and the main patient characteristics at diagnosis and outcome evaluated on the entire cohort. **(a)** Number of events. **(b)** Number of deaths. **(c)** Number of events by age at diagnosis. **(d)** Number of deaths by age at diagnosis. **(e)** Number of events by *MYCN* status. **(f)** Number of deaths by *MYCN* status. **(g)** Number of events by stage at diagnosis. **(h)** Number of deaths by stage at diagnosis.

Supplementary Figure S8

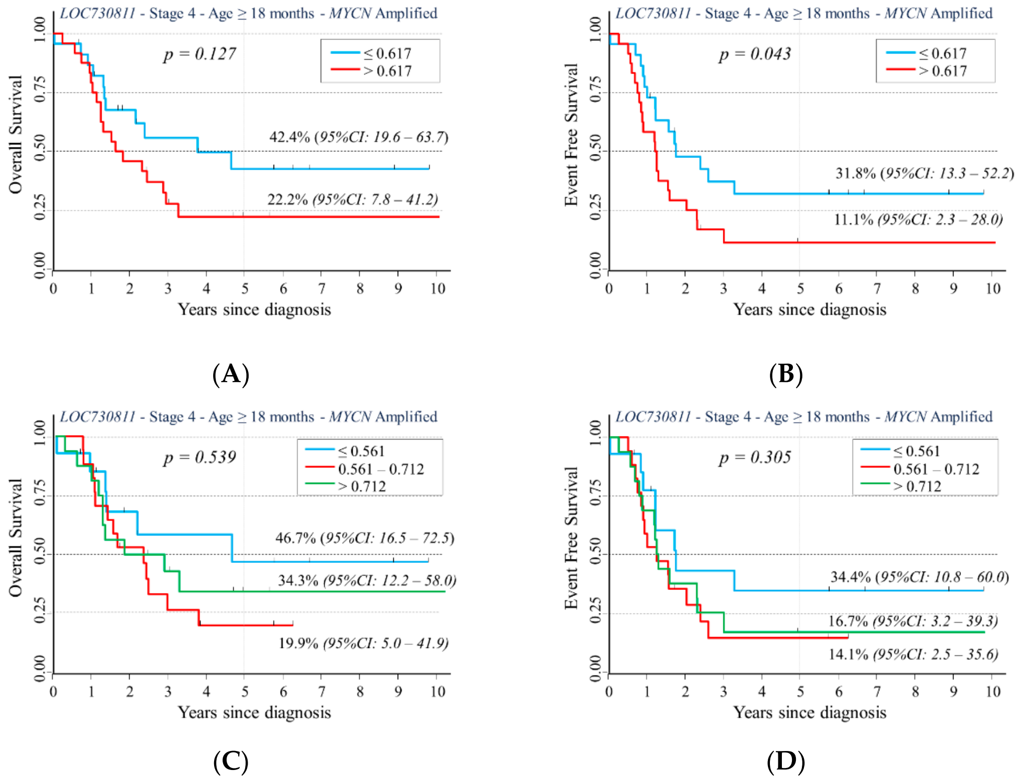

Survival of patients with age ≥ 18 months, stage 4 disease and *MYCN* amplification at diagnosis in relation to *LOC730811* expression level. (**A**) Overall Survival, cut-off based on the median expression value. (**B**) Event Free Survival, cut-off based on the median expression value. (**C**) Overall Survival, cut-offs based on tertile expression values. (**D**) Event Free Survival, cut-offs based on tertile expression values. Five-year survival estimates are displayed.

Supplementary Figure S9

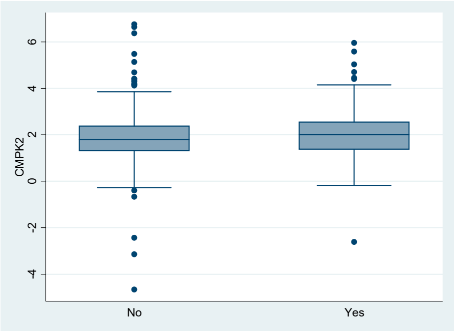

(**a**) p = 0.007

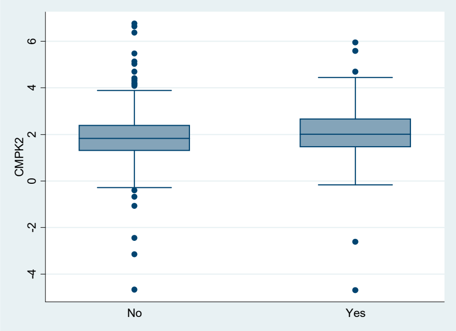

(**b**) p = 0.016

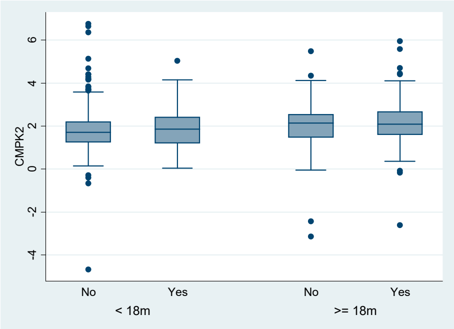

(**c**) p = 0.362; p = 0.963

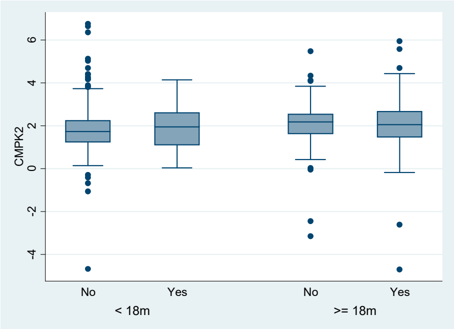

(**d**) p = 0.437; p = 0.487

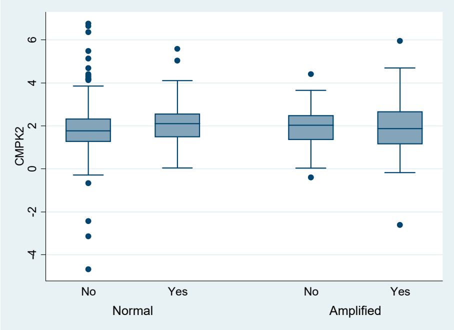

(**e**) p < 0.001; p = 0.661

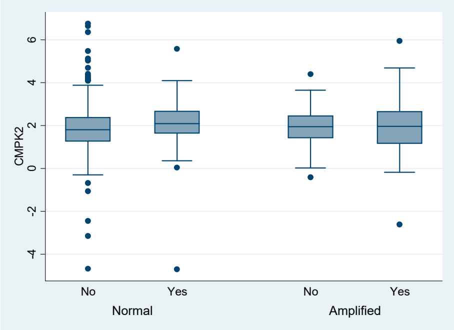

(**f**) p = 0.002; p = 0.933

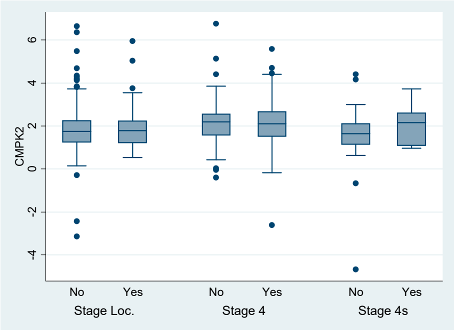

(**g**) p = 0.910; p = 0.800; p = 0.073

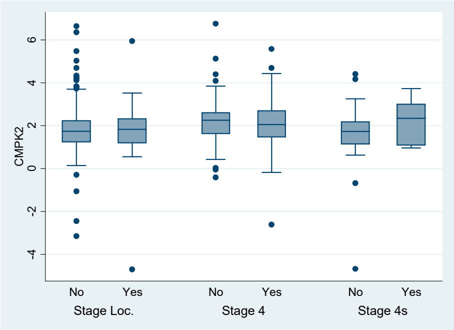

(**h**) p = 0.769; p = 0.290; p = 0.165

Association between *CMPK2* gene expression and the main patient characteristics at diagnosis and outcome evaluated on the entire cohort. **(a)** Number of events. **(b)** Number of deaths. **(c)** Number of events by age at diagnosis. **(d)** Number of deaths by age at diagnosis. **(e)** Number of events by *MYCN* status. **(f)** Number of deaths by *MYCN* status. **g)** Number of events by stage at diagnosis. **(f)** Number of deaths by stage at diagnosis.

Supplementary Figure S10

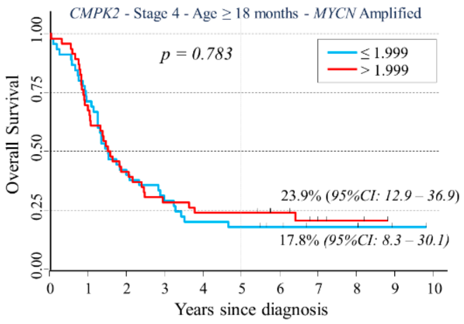

(**A**)

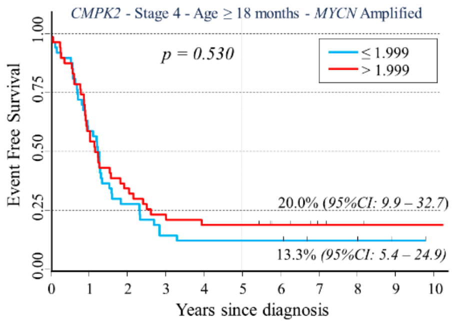

(**B**)

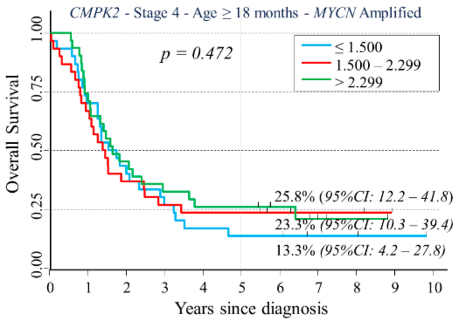

(**C**)

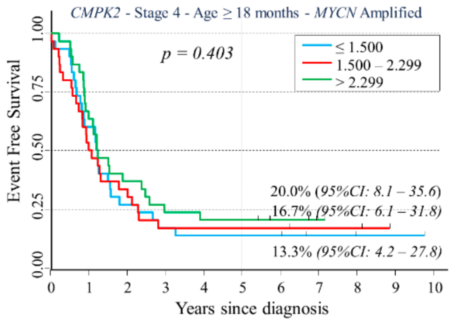

(**D**)

Survival of patients with age ≥ 18 months, stage 4 disease and *MYCN* amplification at diagnosis in relation to *CMPK2* expression level. (**A**) Overall Survival, cut-off based on the median expression value. (**B**) Event Free Survival, cut-off based on the median expression value. (**C**) Overall Survival, cut-offs based on tertile expression values. (**D**) Event Free Survival, cut-offs based on tertile expression values. Five-year survival estimates are displayed.

Supplementary Figure S11

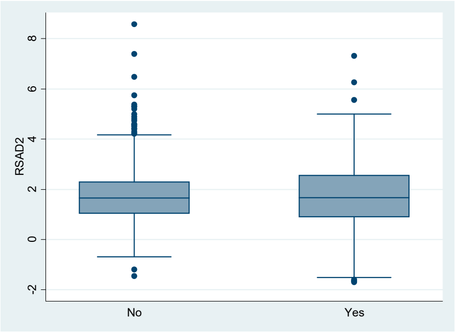

(**a**) p = 0.943

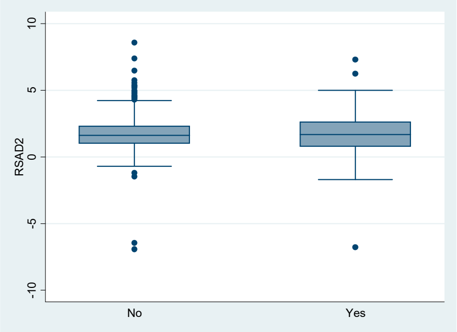

(**b**) p = 0.880

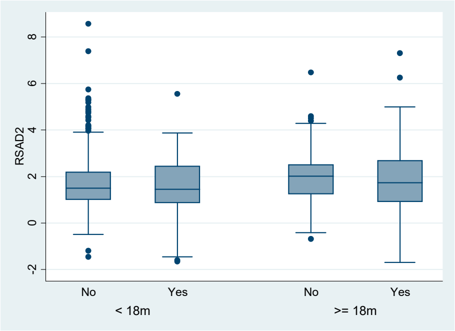

(**c**) p = 0.454; p = 0.254

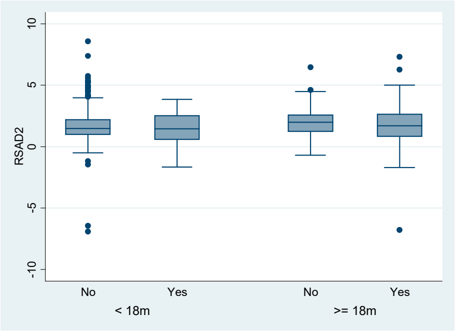

(**d**) p = 0.403; p = 0.134

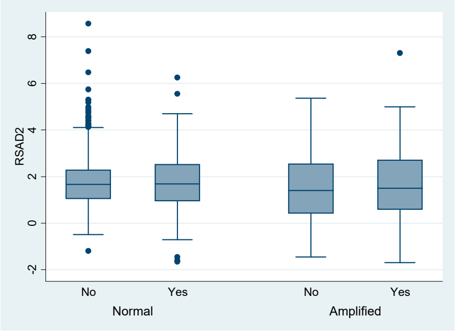

(**e**) p = 0.756; p = 0.869

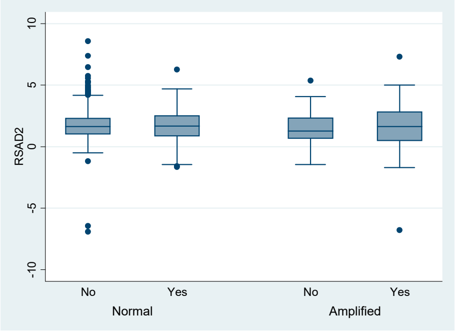

(**f**) p = 0.884; p = 0.701

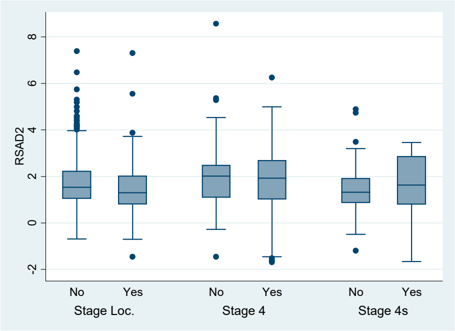

(**g**) p = 0.013; p = 0.521; p = 0.495

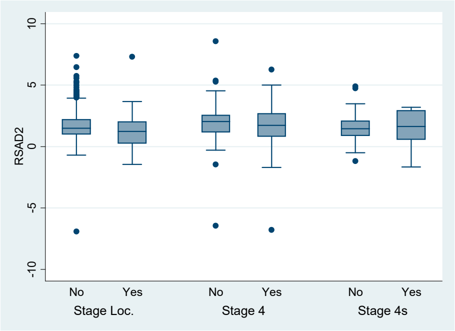

(**h**) p = 0.029; p = 0.247; p = 0.899

Association between *RSAD2* gene expression and the main patient characteristics at diagnosis and outcome evaluated on the entire cohort. **(a)** Number of events. **(b)** Number of deaths. **(c)** Number of events by age at diagnosis. **(d)** Number of deaths by age at diagnosis. **(e)** Number of events by *MYCN* status. **(f)** Number of deaths by *MYCN* status. **g)** Number of events by stage at diagnosis. **(f)** Number of deaths by stage at diagnosis.

Supplementary Figure S12

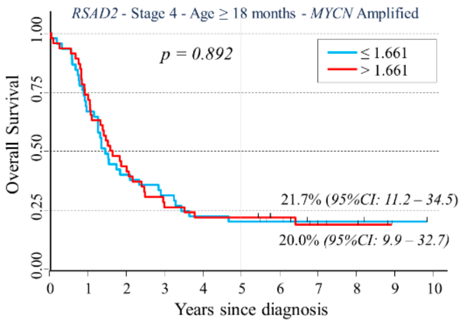

(**A**)

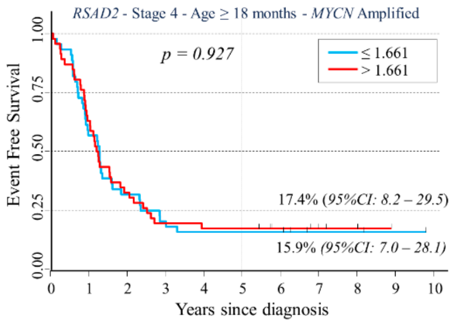

(**B**)

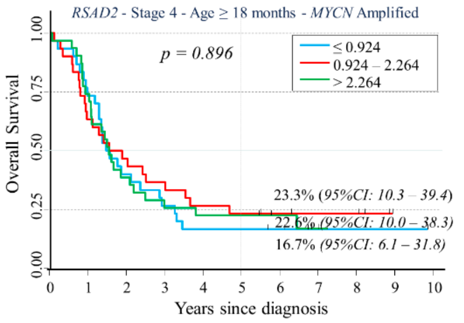

(**C**)

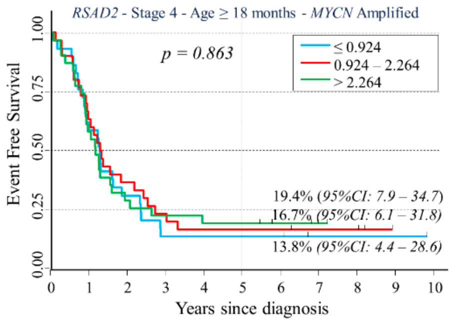

(**D**)

Survival of patients with age ≥ 18 months, stage 4 disease and *MYCN* amplification at diagnosis in relation to *RSAD2* expression level. (**A**) Overall Survival, cut-off based on the median expression value. (**B**) Event Free Survival, cut-off based on the median expression value. (**C**) Overall Survival, cut-offs based on tertile expression values. (**D**) Event Free Survival, cut-offs based on tertile expression values. Five-year survival estimates are displayed.

Supplementary Figure S13

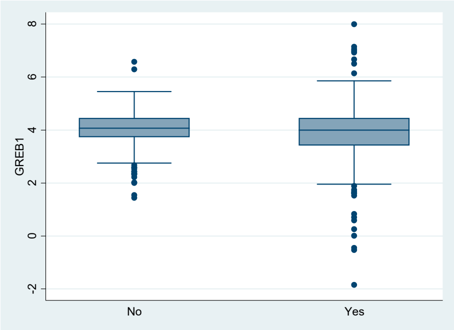

(**a**) p = 0.037

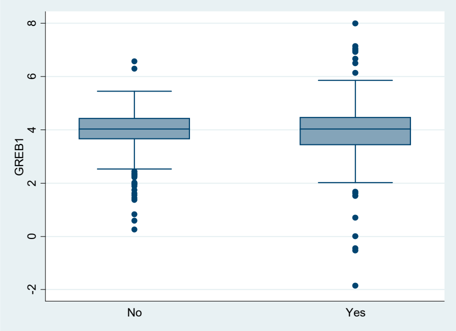

(**b**) p = 0.432

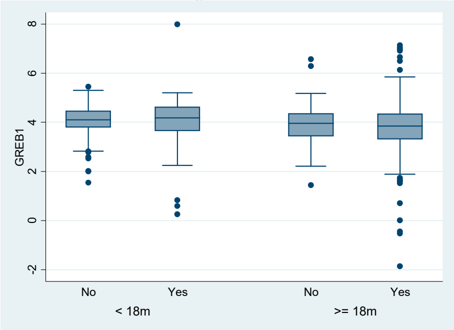

(**c**) p = 0.317; p = 0.209

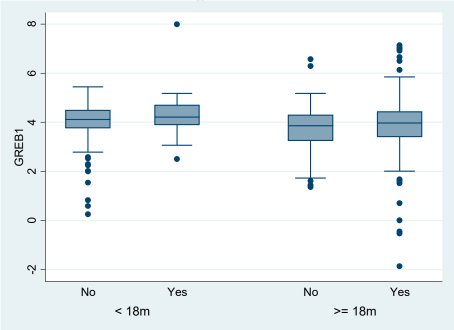

(**d**) p = 0.176; p = 0.269

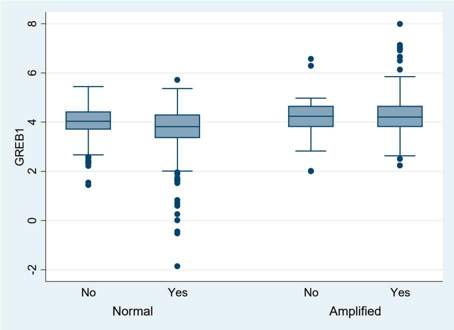

(**e**) p < 0.001; p = 0.946

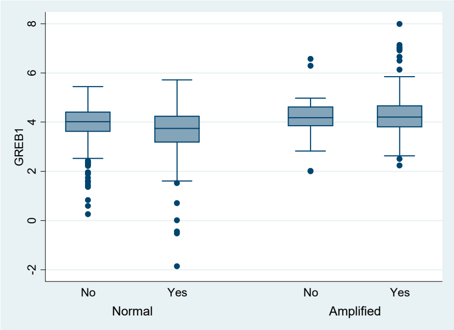

(**f**) p < 0.001; p = 0.770

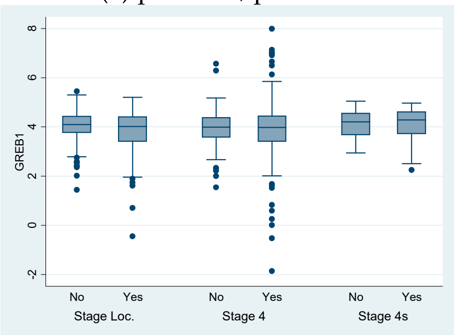

(**g**) p = 0.049; p = 0.757; p = 0.795

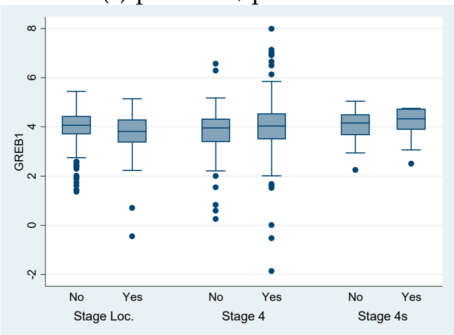

(**h**) p = 0.024; p = 0.176; p = 0.560;.

Association between *GREB1* gene expression and the main patient characteristics at diagnosis and outcome evaluated on the entire cohort. **(a)** Number of events. **(b)** Number of deaths. **(c)** Number of events by age at diagnosis. **(d)** Number of deaths by age at diagnosis. **(e)** Number of events by *MYCN* status. **(f)** Number of deaths by *MYCN* status. **g)** Number of events by stage at diagnosis. **(f)** Number of deaths by stage at diagnosis.

Supplementary Figure S14

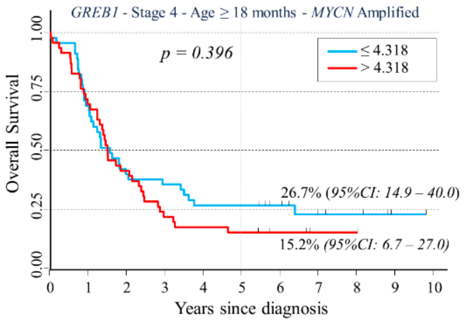

(**A**)

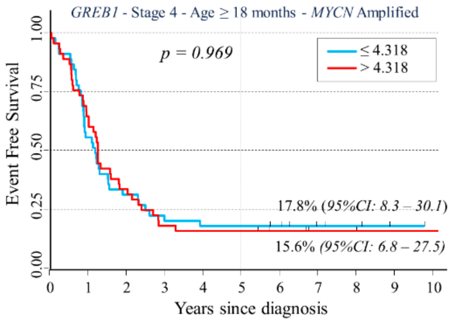

(**B**)

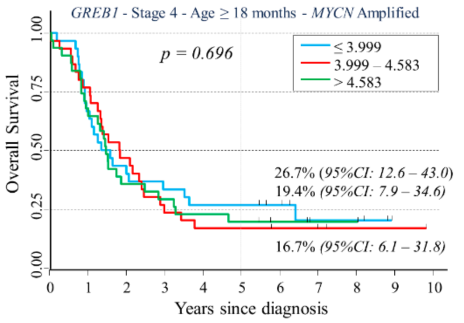

(**C**)

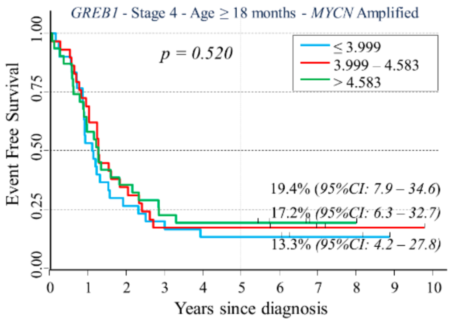

(**D**)

Survival of patients with age ≥ 18 months, stage 4 disease and *MYCN* amplification at diagnosis in relation to *GREB1* expression level. (**A**) Overall Survival, cut-off based on the median expression value. (**B**) Event Free Survival, cut-off based on the median expression value. (**C**) Overall Survival, cut-offs based on tertile expression values. (**D**) Event Free Survival, cut-offs based on tertile expression values. Five-year survival estimates are displayed.

Supplementary Figure S15

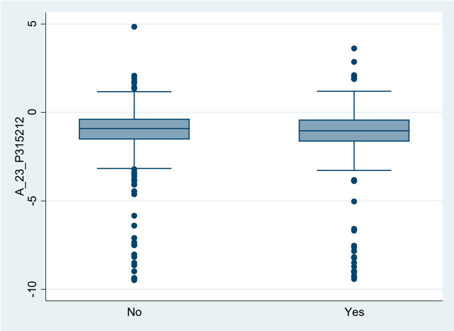

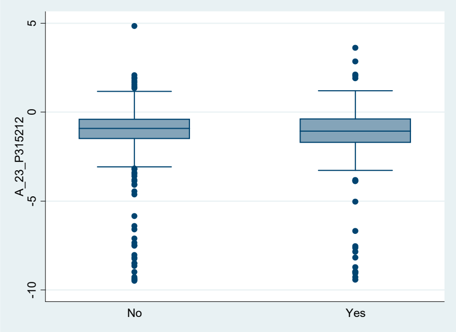

(**a**) p = 0.181

(**b**) p = 0.262

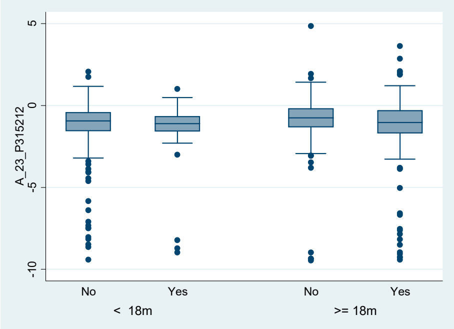

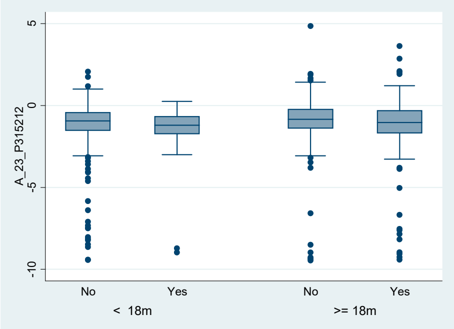

(**c**) p = 0.233; p = 0.149

(**d**) p = 0.095; p = 0.271

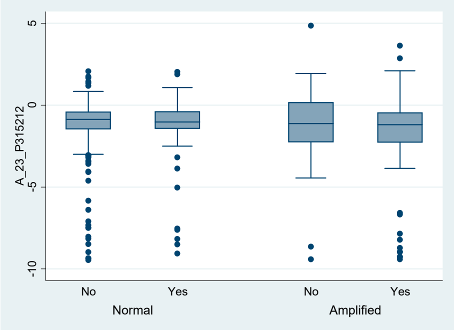

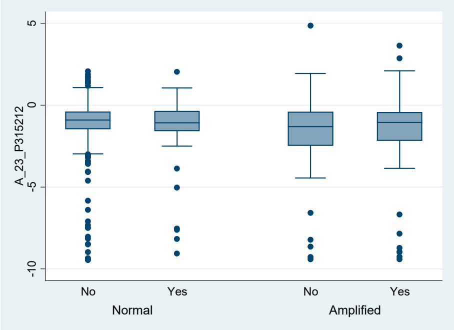

(**e**) p = 0.349; p = 0.848

(**f**) p = 0.514; p = 0.492

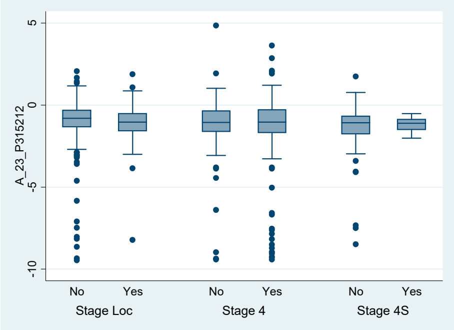

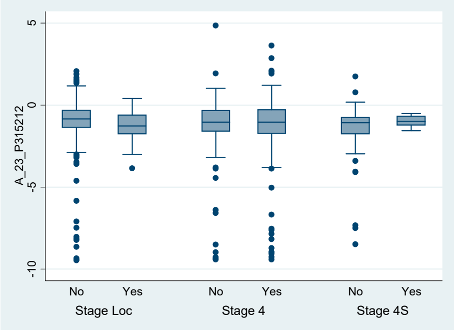

(**g**) p = 0.105; p = 0.882; p = 0.957

(**h**) p = 0.074; p = 0.839; p = 0.454

Association between *NTSR2* gene expression and the main patient characteristics at diagnosis and outcome evaluated on the entire cohort. **(a)** Number of events. **(b)** Number of deaths. **(c)** Number of events by age at diagnosis. **(d)** Number of deaths by age at diagnosis. **(e)** Number of events by *MYCN* status. **(f)** Number of deaths by *MYCN* status. **g)** Number of events by stage at diagnosis. **(f)** Number of deaths by stage at diagnosis.

Supplementary Figure S16

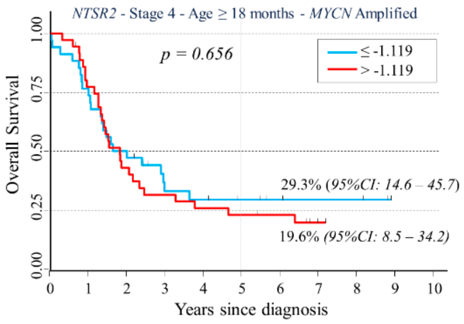

(**A**)

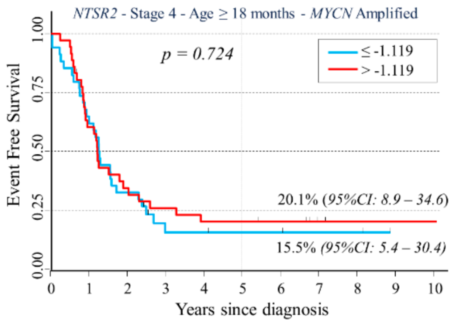

(**B**)

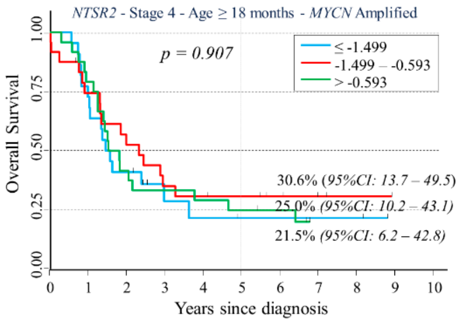

(**C**) p = 0.907

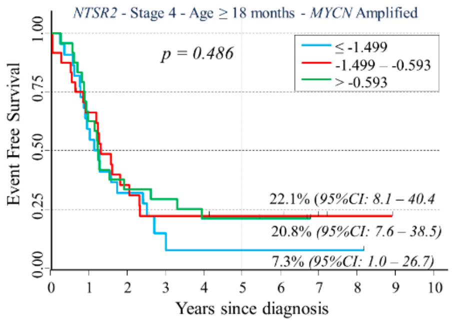

(**D**) p = 0.486

Survival of patients with age ≥ 18 months, stage 4 disease and *MYCN* amplification at diagnosis in relation to *NTSR2* expression level. (**A**) Overall Survival, cut-off based on the median expression value. (**B**) Event Free Survival, cut-off based on the median expression value. (**C**) Overall Survival, cut-offs based on tertile expression values. (**D**) Event Free Survival, cut-offs based on tertile expression values. Five-year survival estimates are displayed.

Supplementary Figure S17

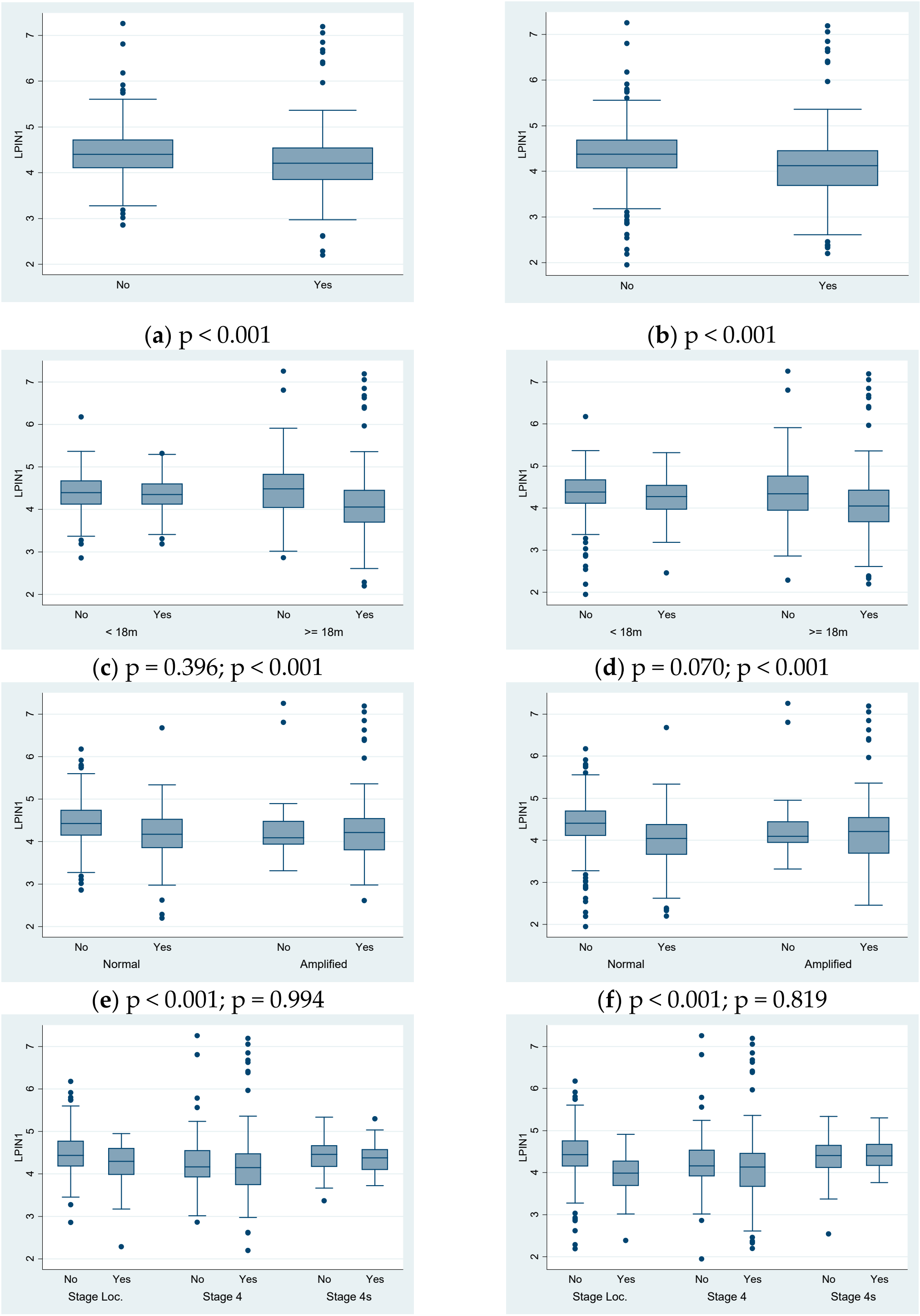

(**a**) p < 0.001

(**b**) p < 0.001

(**c**) p = 0.396; p < 0.001

(**d**) p = 0.070; p < 0.001

(**e**) p < 0.001; p = 0.994

(**f**) p < 0.001; p = 0.819

(**g**) p = 0.001; p = 0.155; p = 0.184

(**h**) p < 0.001; p = 0.160; p = 0.995

Association between *LPIN1* gene expression and the main patient characteristics at diagnosis and outcome evaluated on the entire cohort. **(a)** Number of events. **(b)** Number of deaths. **(c)** Number of events by age at diagnosis. **(d)** Number of deaths by age at diagnosis. **(e)** Number of events by *MYCN* status. **(f)** Number of deaths by *MYCN* status. **g)** Number of events by stage at diagnosis. **(f)** Number of deaths by stage at diagnosis.

Supplementary Figure S18

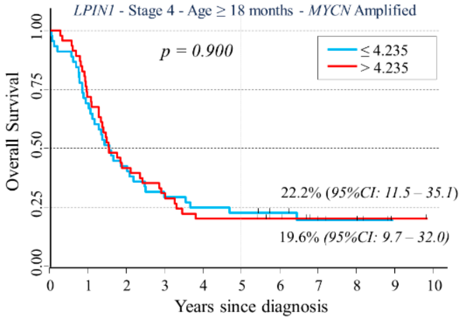

(**A**)

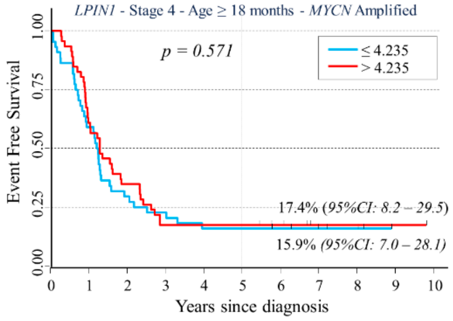

(**B**)

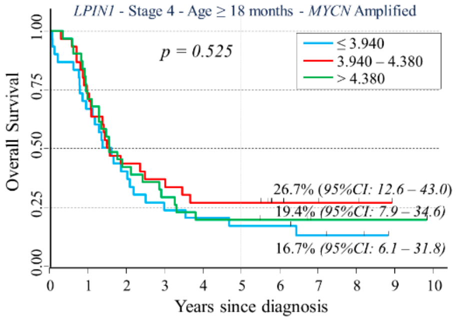

(**C**)

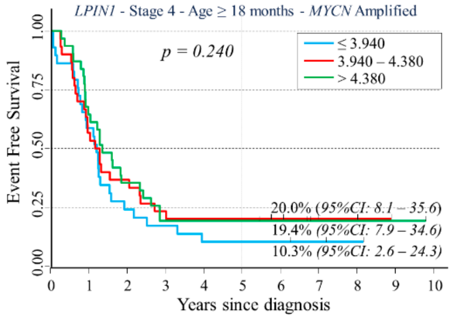

(**D**)

Survival of patients with age ≥ 18 months, stage 4 disease and *MYCN* amplification at diagnosis in relation to *LPIN1* expression level. (**A**) Overall Survival, cut-off based on the median expression value. (**B**) Event Free Survival, cut-off based on the median expression value. (**C**) Overall Survival, cut-offs based on tertile expression values. (**D**) Event Free Survival, cut-offs based on tertile expression values. Five-year survival estimates are displayed.

Supplementary Figure S19

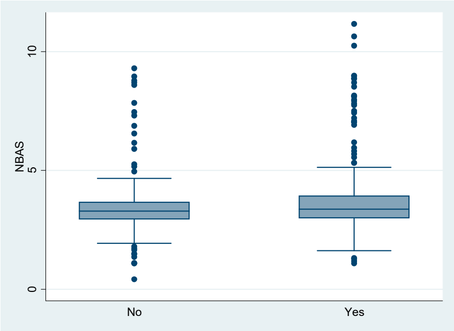

(**a**) p = 0.030

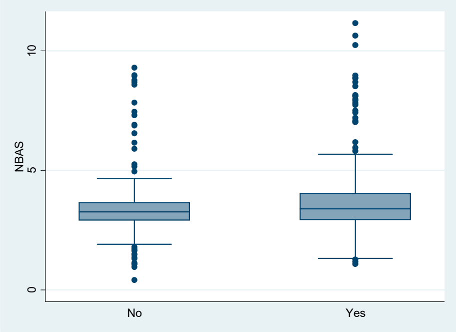

(**b**) p = 0.005

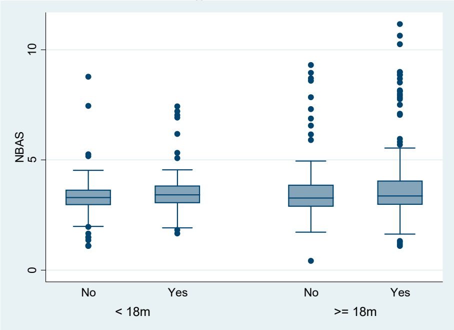

(**c**) p = 0.099; p = 0.284

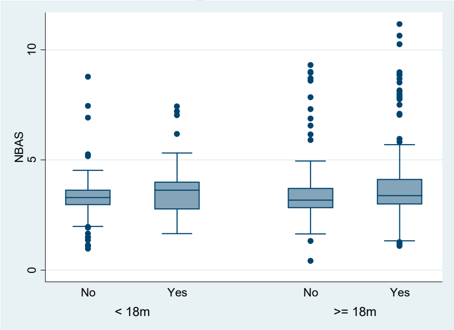

(**d**) p = 0.126; p = 0.031

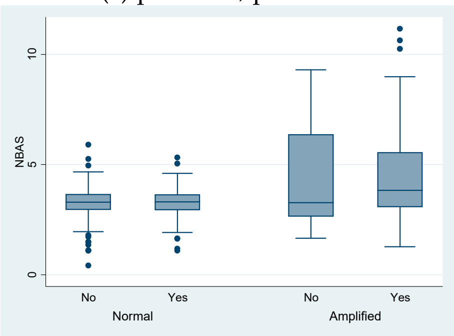

(**e**) p = 0.627; p = 0.277

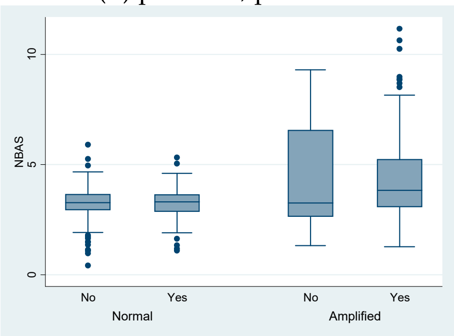

(**f**) p = 0.433; p = 0.239

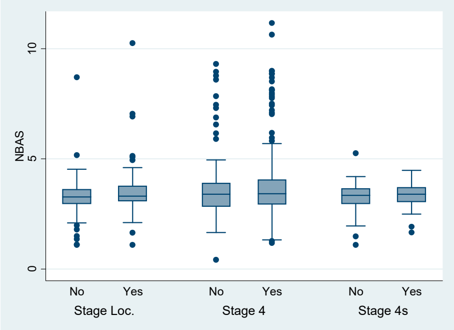

(**g**) p = 0.380; p = 0.340; p = 0.654

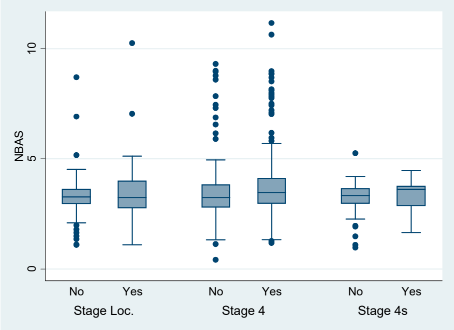

(**h**) p = 0.804; p = 0.037; p = 0.478

Association between *NBAS* gene expression and the main patient characteristics at diagnosis and outcome evaluated on the entire cohort. **(a)** Number of events. **(b)** Number of deaths. **(c)** Number of events by age at diagnosis. **(d)** Number of deaths by age at diagnosis. **(e)** Number of events by *MYCN* status. **(f)** Number of deaths by *MYCN* status. **g)** Number of events by stage at diagnosis. **(f)** Number of deaths by stage at diagnosis.

Supplementary Figure S20

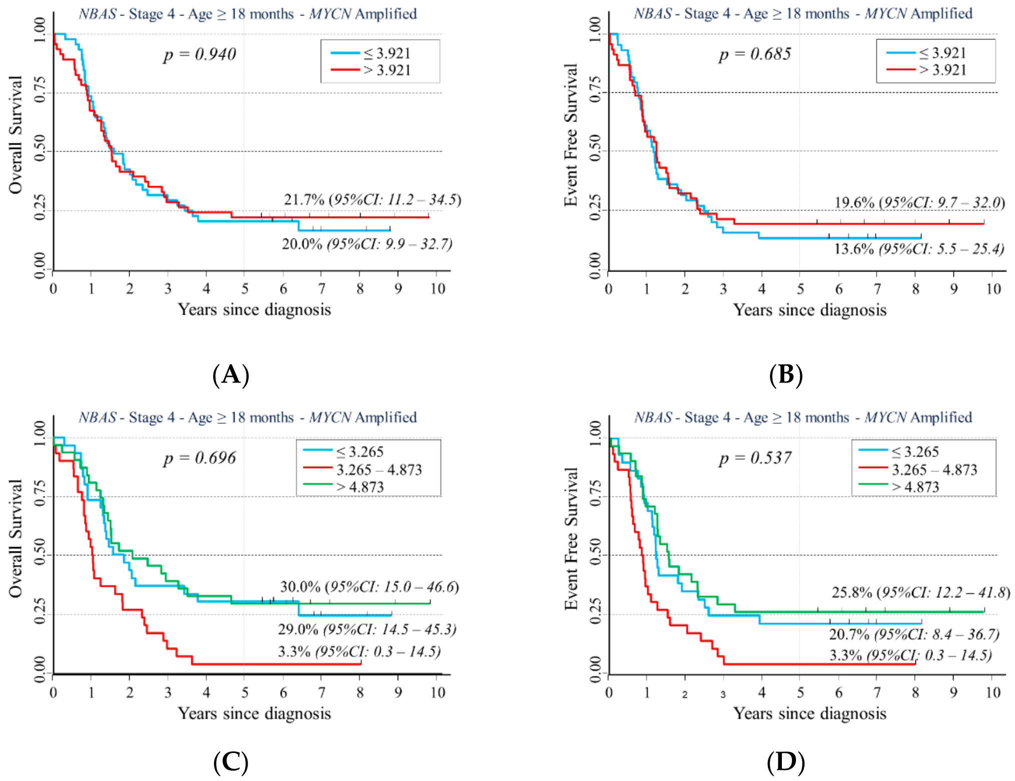

Survival of patients with age ≥ 18 months, stage 4 disease and *MYCN* amplification at diagnosis in relation to *NBAS* expression level. (**A**) Overall Survival, cut-off based on the median expression value. (**B**) Event Free Survival, cut-off based on the median expression value. (**C**) Overall Survival, cut-offs based on tertile expression values. (**D**) Event Free Survival, cut-offs based on tertile expression values. Five-year survival estimates are displayed.

Supplementary Figure S21

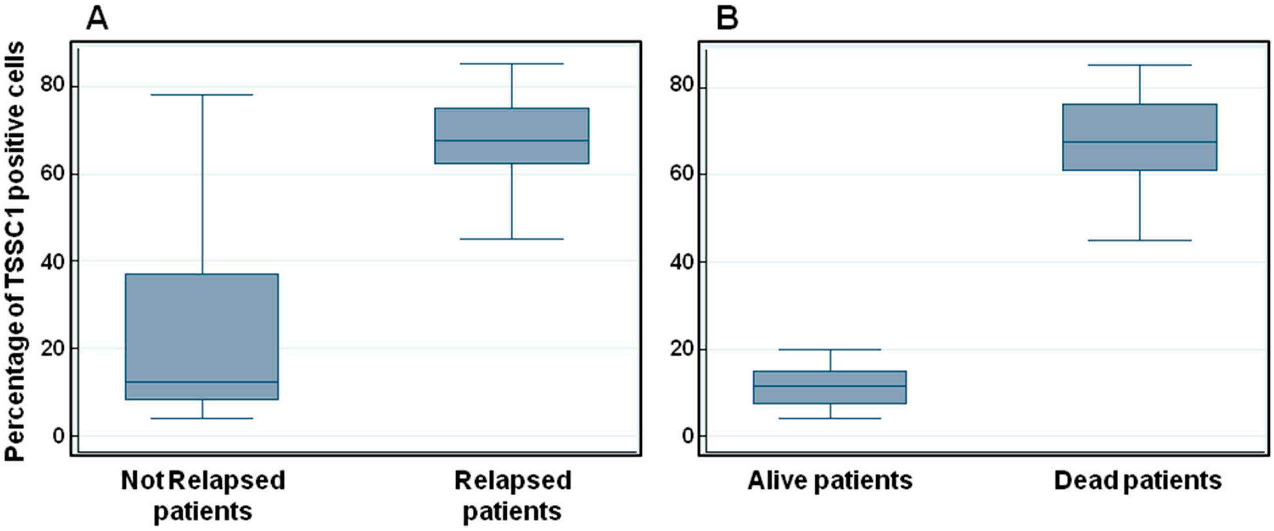

Relative TSSC1 expression levels were plotted in not relapsed or relapsed patients **(A)** and in alive or dead patients **(B)** ($p < 0.001$).

Supplementary Tables

Table S1. Expression level of the target genes in the normal adrenal gland tissue.

Gene	Gene description	Adrenal gland tissue RNA expression*
MYT1L	Myelin transcription factor 1 like	GTEx RNA-seq: 0.5 pTPM
TSSC1	Tumor Suppressing Subtransferable Candidate 1	GTEx RNA-seq: 15.8 pTPM
CMPK2	Cytidine/uridine monophosphate kinase 2	GTEx RNA-seq: 1.5 pTPM
RSAD2	Radical S-adenosyl methionine domain containing 2	GTEx RNA-seq: 2.2 pTPM
RNF144A	Ring finger protein 144A	GTEx RNA-seq: 23.2 pTPM
GREB1	Growth regulation by estrogen in breast cancer 1	GTEx RNA-seq: 3.1 pTPM
NTSR2	Neurotensin Receptor 2	GTEx RNA-seq: 1.0 pTPM
LPIN1	Lipin 1	GTEx RNA-seq: 17.3 pTPM
NBAS	Neuroblastoma amplified sequence	GTEx RNA-seq: 21.2 pTPM

GTEx= Genotype-Tissue Expression
*The mRNA expression data is derived from deep sequencing of RNA (RNA-seq) by GTEx Consortium (Sequencing platform: Illumina HiSeq 2000 or HiSeq X). The RNA-seq tissue data generated by the GTEx project are reported as mean pTPM (protein-coding transcripts per million), hence corresponding to the mean values of the different individual samples from each tissue. The data are included in the Genotype-Tissue Expression (GTEx) database (*GTEx Consortium. The Genotype-Tissue Expression (GTEx) project. Nat Genet. 2013, 45, 580-585).*

Table S2. Overall Survival of 786 NB patients in relation to *RNF144A* expression levels by *MYCN* status.

Gene expression	N/D	OS	95%CI	*p*
MYCN normal				
Cut-off based on the median value				*< 0.001*
≤ 4.572	314/81	73.3	67.8 – 78.0	
> 4.572	315/38	88.6	84.5 – 91.6	
Cut-offs based on tertile values				*< 0.001*
≤ 4.332	209/62	69.2	62.0 – 75.3	
4.332 – 4.820	210/35	84.1	78.4 – 88.4	
> 4.820	210/22	89.5	84.5 – 93.0	
MYCN amplified				
Cut-off based on the median value				*0.218*
≤ 4.383	76/56	26.3	17.0 – 36.5	
> 4.383	77/52	32.0	21.8 – 42.6	
Cut-offs based on tertile values				*0.255*
≤ 4.201	51/36	29.4	17.7 – 42.1	
4.201 – 4.550	51/40	20.6	10.5 – 33.0	
> 4.550	51/32	37.3	24.3 – 50.2	
All patients				
Cut-off based on the median value				*< 0.001*
≤ 4.544	393/151	60.8	55.6 – 65.6	
> 4.544	393/78	80.4	76.1 – 84.1	
Cut-offs based on tertile values				*< 0.001*
≤ 4.299	262/105	58.9	52.4 – 64.8	
4.299 – 4.759	262/81	69.4	63.4 – 74.7	
> 4.759	262/43	83.6	78.5 – 87.6	

N/D = Number of patients/ deaths. OS = Ten-year Overall Survival, 95%CI: 95% confidence interval of OS. N.e. = not evaluable.

Table S3. Overall Survival of 786 NB patients in relation to *RNF144A* expression levels by stage at diagnosis, evaluated by the Kaplan-Meier method.

Gene expression	N/D	OS	95%CI	*p*
Localised stage				
Cut-off based on the median value				*< 0.001*
≤ 4.618	186/27	85.4	79.4 – 89.8	
> 4.618	187/8	95.7	91.6 – 97.8	
Cut-offs based on tertile values				*0.001*
≤ 4.384	124/19	84.6	77.0 – 89.9	
4.384 – 4.853	124/12	90.3	83.5 – 94.4	
> 4.853	125/4	96.8	91.7 – 98.8	
Stage 4				
Cut-off based on the median value				*0.017*
≤ 4.384	160/102	34.4	26.5 – 42.4	
> 4.384	160/81	50.3	42.3 – 57.8	
Cut-offs based on tertile values				*0.009*
≤ 4.178	106/68	34.0	24.4 – 43.9	
4.178 – 4.627	107/67	37.3	28.0 – 46.6	
> 4.627	107/48	56.1	46.2 – 64.9	
Stage 4S				
Cut-off based on the median value				*0.783*
≤ 4.733	46/5	87.0	70.0 – 94.7	
> 4.733	46/6	87.0	73.3 – 93.9	
Cut-offs based on tertile values				*n.e.*
≤ 4.520	30/4	n.e.	n.e.	
4.520 – 4.883	31/3	n.e.	n.e.	
> 4.883	31/4	n.e.	n.e.	
All patients				
Cut-off based on the median value				*< 0.001*
≤ 4.544	393/151	60.8	55.6 – 65.6	
> 4.544	393/78	80.4	76.1 – 84.1	
Cut-offs based on tertile values				*< 0.001*
≤ 4.299	262/105	58.9	52.4 – 64.8	
4.299 – 4.759	2621/81	69.4	63.4 – 74.7	
> 4.759	262/43	83.6	78.5 – 87.6	

N/D = Number of patients/ deaths. OS = Overall Survival, 95%CI: 95% confidence interval of OS. N.e. = not evaluable

Table S4. Event Free Survival of 769 NB patients in relation to *RNF144A* expression levels by *MYCN* status.

Gene expression	N/E	EFS	95%CI	*p*
MYCN normal				
Cut-off based on the median value				*0.001*
≤ 4.572	301/122	59.4	53.6 – 64.7	
> 4.572	313/85	73.2	67.9 – 77.7	
Cut-offs based on tertile values				*0.001*
≤ 4.332	196/87	55.6	48.3 – 62.2	
4.332 – 4.820	208/63	70.2	63.5 – 75.9	
> 4.820	210/57	72.9	66.3 – 78.4	
MYCN amplified				
Cut-off based on the median value				*0.790*
≤ 4.383	74/54	27.0	17.5 – 37.4	
> 4.383	77/57	25.0	15.7 – 35.5	
Cut-offs based on tertile values				*0.962*
≤ 4.201	49/34	30.6	18.5 – 43.6	
4.201 – 4.550	51/40	21.6	11.6 – 33.6	
> 4.550	51/37	26.1	14.6 – 39.3	
All patients				
Cut-off based on the median value				*< 0.001*
≤ 4.544	378/189	50.0	44.8 – 54.9	
> 4.544	391/131	66.7	61.7 – 71.1	
Cut-offs based on tertile values				*< 0.001*
≤ 4.299	247/127	48.6	42.2 – 54.6	
4.299 – 4.759	260/112	57.1	50.8 – 62.9	
> 4.759	262/81	69.1	63.1 – 74.3	

N/E = Number of patients/events. EFS = Ten-year Event Free Survival, 95%CI: 95% confidence interval of EFS.

Table S5. Event Free Survival of 769 NB patients in relation to *RNF144A* expression levels by stage at diagnosis, evaluated by the Kaplan-Meier method.

Gene expression	N/E	EFS	95%CI	*p*
Localised stage				
Cut-off based on the median value				*0.413*
≤ 4.618	178/46	74.2	67.1 – 80.0	
> 4.618	186/40	78.5	71.9 – 83.7	
Cut-offs based on tertile values				*0.571*
≤ 4.384	116/28	75.9	67.0 – 82.7	
4.384 – 4.853	123/33	73.2	64.4 – 80.1	
> 4.853	125/25	80.0	71.9 – 86.0	
Stage 4				
Cut-off based on the median value				*0.003*
≤ 4.384	154/114	26.0	19.3 – 33.1	
> 4.384	160/92	42.9	35.1 – 50.5	
Cut-offs based on tertile values				*0.002*
≤ 4.178	100/75	25.0	17.0 – 33.8	
4.178 – 4.627	107/75	30.8	22.4 – 39.7	
> 4.627	107/56	47.3	37.5 – 56.5	
Stage 4S				
Cut-off based on the median value				*0.772*
≤ 4.733	45/13	71.1	55.5 – 82.1	
> 4.733	45/14	68.9	53.2 – 80.3	
Cut-offs based on tertile values				*0.881*
≤ 4.520	29/11	62.1	42.1 – 76.9	
4.520 – 4.883	30/5	83.3	64.5 – 92.7	
> 4.883	31/11	64.5	45.2 – 78.5	
All patients				
Cut-off based on the median value				*< 0.001*
≤ 4.544	378/189	50.0	44.8 – 54.9	
> 4.544	391/131	66.7	61.7 – 71.1	
Cut-offs based on tertile values				*< 0.001*
≤ 4.299	247/127	48.6	42.2 – 54.6	
4.299 – 4.759	260/112	57.1	50.8 – 62.9	
> 4.759	262/81	69.1	63.1 – 74.3	

N/E = Number of patients/events. EFS = Ten-year Event Free Survival, 95%CI: 95% confidence interval of EFS.

Table S6. Overall Survival of 786 NB patients in relation to *MYT1L* expression levels by *MYCN* status.

Gene expression	N/D	OS	95%CI	*p*
MYCN normal				
Cut-off based on the median value				*< 0.001*
≤ 4.412	314/88	71.7	66.2 – 76.5	
> 4.412	315/31	90.1	86.1 – 93.0	
Cut-offs based on tertile values				*< 0.001*
≤ 4.143	209/68	67.2	60.2 – 73.2	
4.143 – 4.672	210/33	83.8	77.6 – 88.4	
> 4.672	210/18	91.8	87.0 – 94.8	
MYCN amplified				
Cut-off based on the median value				*0.005*
≤ 3.955	76/62	19.7	11.7 – 29.3	
> 3.955	77/46	40.3	29.3 – 50.9	
Cut-offs based on tertile values				*0.018*
≤ 3.695	51/40	23.5	13.0 – 35.8	
3.695 – 4.127	51/39	23.5	13.0 – 35.8	
> 4.127	51/29	43.1	29.4 – 56.1	
All patients				
Cut-off based on the median value				*< 0.001*
≤ 4.316	393/171	56.0	50.7 – 60.9	
> 4.316	393/58	85.2	81.2 – 88.4	
Cut-offs based on tertile values				*< 0.001*
≤ 3.991	262/128	50.7	44.4 – 56.7	
3.991 – 4.589	262/69	72.9	66.7 – 78.1	
> 4.589	262/32	88.1	83.4 – 91.5	

N/D = Number of patients/ deaths. OS = Ten-year Overall Survival, 95%CI: 95% confidence interval of OS. N.e. = not evaluable

Table S7. Overall Survival of 786 NB patients in relation to *MYT1L* expression levels by stage at diagnosis, evaluated by the Kaplan-Meier method.

**Gene expression**	**N/D**	**OS**	**95%CI**	***p***
Localised stage				
Cut-off based on the median value				*< 0.001*
≤ 4.487	186/28	84.8	78.8 – 89.3	
> 4.487	187/7	96.3	92.3 – 98.2	
Cut-offs based on tertile values				*< 0.001*
≤ 4.240	124/26	78.9	70.6 – 85.1	
4.240 – 4.734	124/7	94.4	88.5 – 97.3	
> 4.734	125/2	98.4	93.8 – 99.6	
Stage 4				
Cut-off based on the median value				*0.017*
≤ 3.980	160/102	35.8	28.2 – 43.4	
> 3.980	160/81	49.1	40.7 – 57.0	
Cut-offs based on tertile values				*0.009*
≤ 3.727	106/65	38.6	29.1 – 48.0	
3.727 – 4.302	107/71	30.8	21.3 – 40.7	
> 4.302	107/47	57.4	47.3 – 66.2	
Stage 4S				
Cut-off based on the median value				*0.679*
≤ 4.580	46/6	87.0	73.3 – 93.9	
> 4.580	46/5	88.7	74.9 – 95.2	
Cut-offs based on tertile values				*n.e.*
≤ 4.320	30/5	n.e.	n.e.	
4.320 – 4.763	31/2	n.e.	n.e.	
> 4.763	31/4	n.e.	n.e.	
All patients				
Cut-off based on the median value				*< 0.001*
≤ 4.316	393/171	56.0	50.7 – 60.9	
> 4.316	393/58	85.2	81.2 – 88.4	
Cut-offs based on tertile values				*< 0.001*
≤ 3.991	262/128	50.7	44.4 – 56.7	
3.991 – 4.589	262/69	72.9	66.7 – 78.1	
> 4.589	262/32	88.1	83.4 – 91.5	

N/D = Number of patients/ deaths. OS = Ten-year Overall Survival, 95%CI: 95% confidence interval of OS. N.e. = not evaluable

Table S8. Event Free Survival of 769 NB patients in relation to *MYT1L* expression levels by *MYCN* status.

Gene expression	N/E	EFS	95%CI	*p*
MYCN normal				
Cut-off based on the median value				*< 0.001*
≤ 4.412	303/133	56.4	50.7 – 61.8	
> 4.412	311/74	76.2	71.0 – 80.5	
Cut-offs based on tertile values				*< 0.001*
≤ 4.143	201/97	52.2	45.1 – 58.9	
4.143 – 4.672	205/68	66.8	59.9 – 72.8	
> 4.672	208/42	79.8	73.7 – 84.7	
MYCN amplified				
Cut-off based on the median value				*0.014*
≤ 3.955	75/62	17.3	9.8 – 26.7	
> 3.955	76/49	34.5	23.8 – 45.5	
Cut-offs based on tertile values				*0.065*
≤ 3.695	50/40	20.0	10.3 – 32.0	
3.695 – 4.127	51/39	23.5	13.0 – 35.8	
> 4.127	50/32	35.1	22.0 – 48.5	
All patients				
Cut-off based on the median value				*< 0.001*
≤ 4.316	393/171	56.0	50.7 – 60.9	
> 4.316	393/58	85.2	81.2 – 88.4	
Cut-offs based on tertile values				*< 0.001*
≤ 3.991	262/128	50.7	44.4 – 56.7	
3.991 – 4.589	262/69	72.9	66.7 – 78.1	
> 4.589	262/32	88.1	83.4 – 91.5	

N/E = Number of patients/events. EFS = Ten-year Event Free Survival, 95%CI: 95% confidence interval of EFS.

Table S9. Event Free Survival of 769 NB patients in relation to *MYT1L* expression levels by stage at diagnosis, evaluated by the Kaplan-Meier method.

**Gene expression**	**N/E**	**EFS**	**95%CI**	***p***
Localised stage				
Cut-off based on the median value				*0.003*
≤ 4.487	180/55	69.4	62.2 – 75.6	
> 4.487	184/31	83.2	76.9 – 87.8	
Cut-offs based on tertile values				*0.002*
≤ 4.240	120/40	66.7	57.5 – 74.3	
4.240 – 4.734	120/29	75.8	67.1 – 82.5	
> 4.734	124/17	86.3	78.9 – 91.2	
Stage 4				
Cut-off based on the median value				*0.023*
≤ 3.980	157/111	29.3	22.4 – 36.5	
> 3.980	157/95	39.9	32.2 – 47.5	
Cut-offs based on tertile values				*0.007*
≤ 3.727	103/72	30.1	21.6 – 39.1	
3.727 – 4.302	106/77	28.0	19.7 – 36.8	
> 4.302	105/57	45.7	36.0 – 54.9	
Stage 4S				
Cut-off based on the median value				*0.404*
≤ 4.580	44/15	65.9	50.0 – 77.9	
> 4.580	46/12	73.9	58.7 – 84.3	
Cut-offs based on tertile values				*0.252*
≤ 4.320	28/12	57.1	37.1 – 73.0	
4.320 – 4.763	31/6	80.7	61.9 – 90.8	
> 4.763	31/9	71.0	51.6 – 83.7	
All patients				
Cut-off based on the median value				*< 0.001*
≤ 4.316	383/212	44.8	39.7 – 49.7	
> 4.316	386/108	72.0	67.2 – 76.2	
Cut-offs based on tertile values				*< 0.001*
≤ 3.991	253/151	40.3	34.3 – 46.3	
3.991 – 4.589	257/107	58.5	52.2 – 64.3	
> 4.589	259/62	76.1	70.4 – 80.8	

N/E = Number of patients/events. EFS = Event Free Survival, 95%CI: 95% confidence interval of EFS.

Table S10. Overall Survival of 786 NB patients in relation to *TSSC1* expression levels by *MYCN* status.

Gene expression	N/D	OS	95%CI	*p*
MYCN normal				
Cut-off based on the median value				*< 0.001*
≤ 3.312	314/39	87.3	82.9 – 90.6	
> 3.312	315/80	74.5	69.0 – 79.1	
Cut-offs based on tertile values				*< 0.001*
≤ 3.122	209/24	87.9	82.5 – 91.8	
3.122– 3.473	210/32	85.6	80.0 – 89.7	
> 3.473	210/63	69.2	62.0 – 75.3	
MYCN amplified				
Cut-off based on the median value				*0.039*
≤ 3.540	76/48	36.8	26.2 – 47.5	
> 3.540	77/60	21.6	13.1 – 31.5	
Cut-offs based on tertile values				*0.025*
≤ 3.335	51/32	37.3	24.3 – 50.2	
3.335 – 3.769	51/33	35.3	22.6 – 48.2	
> 3.769	51/43	14.7	6.4 – 26.3	
All patients				
Cut-off based on the median value				*< 0.001*
≤ 3.346	393/75	80.6	76.2 – 84.2	
> 3.346	393/154	60.6	55.4 – 65.3	
Cut-offs based on tertile values				*< 0.001*
≤ 3.162	262/43	82.9	77.6 – 87.1	
3.162 – 3.520	262/66	75.3	69.6 – 80.1	
> 3.520	262/120	53.5	47.0 – 59.6	

N/D = Number of patients/ deaths. OS = Ten-year Overall Survival, 95%CI: 95% confidence interval of OS. N.e. = not evaluable

Table S11. Overall Survival of 786 NB patients in relation to *TSSC1* expression levels by stage at diagnosis, evaluated by the Kaplan-Meier method.

Gene expression	N/D	OS	95%CI	*p*
Localised stage				
Cut-off based on the median value				*0.007*
≤ 3.286	186/10	94.6	90.1 – 97.0	
> 3.286	187/25	86.6	80.8 – 90.7	
Cut-offs based on tertile values				*< 0.001*
≤ 3.124	124/6	95.2	89.6 – 97.8	
3.124 – 3.421	124/8	93.4	87.3 – 96.7	
> 3.421	125/21	83.1	75.3 – 88.7	
Stage 4				
Cut-off based on the median value				*0.003*
≤ 3.504	160/80	50.0	41.7 – 57.7	
> 3.504	160/103	34.9	27.2 – 42.6	
Cut-offs based on tertile values				*< 0.001*
≤ 3.273	106/50	52.2	41.9 – 61.6	
3.273 – 3.713	107/60	43.9	34.1 – 53.3	
> 3.713	107/73	31.2	22.2 – 40.6	
Stage 4S				
Cut-off based on the median value				*0.694*
≤ 3.241	46/5	88.5	74.3 – 95.1	
> 3.241	46/6	87.0	73.3 – 93.9	
Cut-offs based on tertile values				*n.e.*
≤ 3.111	30/2	n.e.	n.e.	
3.111 – 3.367	31/4	n.e.	n.e.	
> 3.367	31/5	n.e.	n.e.	
All patients				
Cut-off based on the median value				*< 0.001*
≤ 3.346	393/75	80.6	76.2 – 84.2	
> 3.346	393/154	60.6	55.4 – 65.3	
Cut-offs based on tertile values				*< 0.001*
≤ 3.162	262/43	82.9	77.6 – 87.1	
3.162 – 3.520	262/66	75.3	69.6 – 80.1	
> 3.520	262/120	53.5	47.0 – 59.6	

N/D = Number of patients/ deaths. OS = Ten year Overall Survival, 95%CI: 95% confidence interval of OS. N.e. = not evaluable

Table S12. Event Free Survival of 786 NB patients in relation to *TSSC1* expression levels by *MYCN* status.

**Gene expression**	**N/E**	**EFS**	**95%CI**	***p***
MYCN normal				
Cut-off based on the median value				*0.264*
≤ 3.312	306/98	68.3	62.7 – 73.2	
> 3.312	308/109	64.6	59.0 – 69.7	
Cut-offs based on tertile values				*0.065*
≤ 3.122	204/62	70.0	63.2 – 75.8	
3.122– 3.473	205/68	66.8	59.9 – 72.8	
> 3.473	205/77	62.4	55.4 – 68.7	
MYCN amplified				
Cut-off based on the median value				*0.095*
≤ 3.540	75/50	33.3	23.0 – 44.0	
> 3.540	76/61	19.0	10.8 – 28.8	
Cut-offs based on tertile values				*0.039*
≤ 3.335	50/32	36.0	23.1 – 49.1	
3.335 – 3.769	51/36	27.9	15.9 – 41.2	
> 3.769	50/43	14.0	6.2 – 25.0	
All patients				
Cut-off based on the median value				*< 0.001*
≤ 3.346	383/138	64.2	59.2 – 68.8	
> 3.346	386/182	52.7	47.6 – 57.6	
Cut-offs based on tertile values				*< 0.001*
≤ 3.162	256/89	65.6	59.4 – 71.1	
3.162 – 3.520	257/97	62.3	56.0 – 67.9	
> 3.520	256/134	47.4	41.1 – 53.4	

N/E = Number of patients/events. EFS = Ten-year Event Free Survival, 95%CI: 95% confidence interval of EFS.

Table S13. Event Free Survival of 769 NB patients in relation to *TSSC1* expression levels by stage at diagnosis, evaluated by the Kaplan-Meier method.

Gene expression	N/E	EFS	95%CI	*p*
Localised stage				
Cut-off based on the median value				*0.790*
≤ 3.286	181/44	75.7	68.8 – 81.3	
> 3.286	183/42	77.1	70.3 – 82.5	
Cut-offs based on tertile values				*0.640*
≤ 3.124	121/26	78.5	70.1 – 84.8	
3.124 – 3.421	121/31	74.4	65.6 – 81.2	
> 3.421	122/29	76.2	67.6 – 82.8	
Stage 4				
Cut-off based on the median value				*0.020*
≤ 3.504	157/97	38.9	31.2 – 46.4	
> 3.504	157/109	30.3	23.2 – 37.6	
Cut-offs based on tertile values				*0.003*
≤ 3.273	103/63	39.8	30.4 – 49.1	
3.273 – 3.713	106/65	38.3	29.0 – 47.5	
> 3.713	105/78	25.7	17.8 – 34.3	
Stage 4S				
Cut-off based on the median value				*0.436*
≤ 3.241	45/12	73.3	57.9 – 83.9	
> 3.241	45/15	66.7	50.9 – 78.4	
Cut-offs based on tertile values				*0.375*
≤ 3.111	29/7	75.9	55.9 – 87.7	
3.111 – 3.367	30/10	66.7	46.9 – 80.5	
> 3.367	31/10	67.7	48.4 – 81.2	
All patients				
Cut-off based on the median value				*< 0.001*
≤ 3.346	383/138	64.2	59.2 – 68.8	
> 3.346	386/182	52.7	47.6 – 57.6	
Cut-offs based on tertile values				*< 0.001*
≤ 3.162	256/89	65.6	59.4 – 71.1	
3.162 – 3.520	257/97	62.3	56.0 – 67.9	
> 3.520	256/134	47.4	41.1 – 53.4	

N/E = Number of patients/events. EFS = Ten-year Event Free Survival, 95%CI: 95% confidence interval of EFS.

Table S14. Overall Survival of 498 NB patients in relation to *LOC730811* expression levels by *MYCN* status.

Gene expression	N/D	OS	95%CI	*p*
MYCN normal				
Cut-off based on the median value				*0.527*
≤ 0.511	200/24	85.5	78.8 – 90.2	
> 0.511	201/29	83.7	77.0 – 88.6	
Cut-offs based on tertile values				*0.637*
≤ 0.414	133/19	83.0	74.4 – 89.0	
0.414 – 0.604	134/17	83.2	73.5 – 89.6	
> 0.604	134/17	87.2	79.9 – 92.0	
MYCN amplified				
Cut-off based on the median value				*0.624*
≤ 0.685	46/27	32.9	18.3 – 48.3	
> 0.685	46/24	41.8	26.4 – 56.4	
Cut-offs based on tertile values				*0.535*
≤ 0.570	30/17	34.7	16.8 – 53.2	
0.570 – 0.811	31/18	30.0	12.3 – 50.0	
> 0.811	31/16	45.4	26.8 – 62.3	
All patients				
Cut-off based on the median value				*0.005*
≤ 0.535	248/40	80.6	74.1 – 85.6	
> 0.535	250/65	71.9	65.3 – 77.4	
Cut-offs based on tertile values				*0.096*
≤ 0.438	166/31	79.1	71.4 – 84.9	
0.438 – 0.638	166/32	76.3	67.5 – 83.1	
> 0.638	166/42	73.3	65.5 – 79.6	

N/D = Number of patients/ deaths. OS = Ten-year Overall Survival, 95%CI: 95% confidence interval of OS. N.e. = not evaluable

Table S15. Overall Survival of 498 NB patients in relation to *LOC730811* expression levels by stage at diagnosis, evaluated by the Kaplan-Meier method.

Gene expression	N/D	OS	95%CI	*p*
Localised stage				
Cut-off based on the median value				*0.784*
≤ 0.497	131/10	91.0	83.6 – 95.1	
> 0.497	131/9	92.5	86.1 – 96.1	
Cut-offs based on tertile values				*0.540*
≤ 0.424	87/8	88.5	77.8 – 94.2	
0.424 – 0.624	87/5	93.8	85.8 – 97.4	
> 0.624	88/6	92.5	84.0 – 96.6	
Stage 4				
Cut-off based on the median value				*0.071*
≤ 0.582	91/36	50.2	36.9 – 62.1	
> 0.582	92/46	45.9	34.7 – 56.3	
Cut-offs based on tertile values				*0.322*
≤ 0.511	59/24	50.4	34.2 – 64.5	
0.511 – 0.713	63/30	44.6	29.8 – 58.4	
> 0.713	61/28	48.9	34.5 – 61.8	
Stage 4S				
Cut-off based on the median value				*n.e.*
≤ 0.483	26/3	n.e.	n.e.	
> 0.483	27/1	n.e.	n.e.	
Cut-offs based on tertile values				*n.e.*
≤ 0.376	17/1	n.e.	n.e.	
0.376 – 0.566	18/3	n.e.	n.e.	
> 0.566	17/0	n.e.	n.e.	
All patients				
Cut-off based on the median value				*0.005*
≤ 0.535	248/40	80.6	74.1 – 85.6	
> 0.535	250/65	71.9	65.3 – 77.4	
Cut-offs based on tertile values				*0.096*
≤ 0.438	166/31	79.1	71.4 – 84.9	
0.438 – 0.638	166/32	76.3	67.5 – 83.1	
> 0.638	166/42	73.3	65.5 – 79.6	

N/D = Number of patients/ deaths. OS = Ten-year Overall Survival, 95%CI: 95% confidence interval of OS. N.e. = not evaluable.

Table S16. Event Free Survival of 498 NB patients in relation to *LOC730811* expression levels by *MYCN* status.

Gene expression	N/E	EFS	95%CI	*p*
MYCN normal				
Cut-off based on the median value				*0.956*
≤ 0.511	200/60	68.8	61.7 – 74.9	
> 0.511	201/60	68.8	61.7 – 74.9	
Cut-offs based on tertile values				*0.134*
≤ 0.414	133/46	64.3	55.2 – 72.0	
0.414 – 0.604	134/40	68.5	59.5 – 75.9	
> 0.604	134/34	73.6	65.1 – 80.4	
MYCN amplified				
Cut-off based on the median value				*0.670*
≤ 0.685	46/31	27.2	14.7 – 41.3	
> 0.685	46/29	32.2	18.8 – 46.5	
Cut-offs based on tertile values				*0.390*
≤ 0.570	30/20	26.2	11.4 – 43.8	
0.570 – 0.811	31/22	22.1	8.8 – 39.1	
> 0.811	31/18	40.1	22.7 – 57.0	
All patients				
Cut-off based on the median value				*0.073*
≤ 0.535	248/82	65.5	59.0 – 71.2	
> 0.535	250/101	57.8	51.2 – 63.8	
Cut-offs based on tertile values				*0.877*
≤ 0.438	166/63	60.9	52.9 – 68.1	
0.438 – 0.638	166/60	62.0	53.9 – 69.2	
> 0.638	166/60	62.0	53.9 – 69.1	

N/E = Number of patients/events. EFS = Ten-year Event Free Survival, 95%CI: 95% confidence interval of EFS.

Table S17. Event Free Survival of 498 NB patients in relation to *LOC730811* expression levels by stage at diagnosis, evaluated by the Kaplan-Meier method.

Gene expression	N/E	EFS	95%CI	*p*
Localised stage				
Cut-off based on the median value				*0.689*
≤ 0.497	131/29	77.3	68.9 – 83.6	
> 0.497	131/26	79.6	71.5 – 85.6	
Cut-offs based on tertile values				*0.848*
≤ 0.424	87/18	79.1	68.9 – 86.3	
0.424 – 0.624	87/20	76.3	65.6 – 84.0	
> 0.624	88/17	80.2	70.1 – 87.2	
Stage 4				
Cut-off based on the median value				*0.906*
≤ 0.582	91/60	30.1	20.6 – 40.2	
> 0.582	92/55	35.6	25.4 – 45.9	
Cut-offs based on tertile values				*0.794*
≤ 0.511	59/39	30.2	18.7 – 42.5	
0.511 – 0.713	63/42	30.9	19.5 – 43.0	
> 0.713	61/34	38.5	25.5 – 51.3	
Stage 4S				
Cut-off based on the median value				*n.e.*
≤ 0.483	26/8	n.e.	n.e.	
> 0.483	27/5	n.e.	n.e.	
Cut-offs based on tertile values				*n.e.*
≤ 0.376	17/6	n.e.	n.e.	
0.376 – 0.566	18/3	n.e.	n.e.	
> 0.566	18/4	n.e.	n.e.	
All patients				
Cut-off based on the median value				*0.073*
≤ 0.535	248/82	65.5	59.0 – 71.2	
> 0.535	250/101	57.8	51.2 – 63.8	
Cut-offs based on tertile values				*0.877*
≤ 0.438	166/63	60.9	52.9 – 68.1	
0.438 – 0.638	166/60	62.0	53.9 – 69.2	
> 0.638	166/60	62.0	53.9 – 69.1	

N/E = Number of patients/events. EFS = Ten-year Event Free Survival, 95%CI: 95% confidence interval of EFS.

Table S18. Overall Survival of 786 NB patients in relation to *CMPK2* expression levels by *MYCN* status.

Gene expression	N/D	OS	95%CI	*p*
MYCN normal				
Cut-off based on the median value				*0.007*
≤ 1.863	314/46	85.2	80.6 – 88.8	
> 1.863	315/73	76.6	71.4 – 81.1	
Cut-offs based on tertile values				*0.003*
≤ 1.552	209/26	87.2	81.8 – 91.1	
1.552 – 2.231	210/42	80.6	74.5 – 85.4	
> 2.231	210/51	74.9	68.0 – 80.5	
MYCN amplified				
Cut-off based on the median value				*0.995*
≤ 1.954	76/54	29.0	19.3 – 39.3	
> 1.954	77/54	29.1	19.2 – 39.7	
Cut-offs based on tertile values				*0.330*
≤ 1.461	51/38	25.5	14.6 – 37.9	
1.461 – 2.320	51/36	29.4	17.7 – 42.1	
> 2.320	51/34	32.4	19.8 – 45.6	
All patients				
Cut-off based on the median value				*0.020*
≤ 1.877	393/99	74.7	70.0 – 78.7	
> 1.877	393/130	66.5	61.4 – 71.1	
Cut-offs based on tertile values				*0.047*
≤ 1.541	262/66	74.5	68.7 – 79.4	
1.541 – 2.250	262/74	72.2	66.3 – 77.3	
> 2.250	262/89	64.8	58.3 – 70.6	

N/D = Number of patients/ deaths. OS = Ten-year Overall Survival, 95%CI: 95% confidence interval of OS. N.e. = not evaluable

Table S19. Overall Survival of 786 NB patients in relation to *CMPK2* expression levels by stage at diagnosis, evaluated by the Kaplan-Meier method.

Gene expression	N/D	OS	95%CI	*p*
Localised stage				
Cut-off based on the median value				*0.413*
≤ 1.754	186/15	91.9	87.0 – 95.1	
> 1.754	187/20	89.2	83.8 – 92.9	
Cut-offs based on tertile values				*0.881*
≤ 1.396	124/12	90.3	83.6 – 94.4	
1.396 – 2.046	124/10	91.9	85.4 – 95.5	
> 2.046	125/13	89.5	82.7 – 93.8	
Stage 4				
Cut-off based on the median value				*0.019*
≤ 2.121	160/101	37.7	30.2 – 45.2	
> 2.121	160/82	47.0	38.5 – 55.0	
Cut-offs based on tertile values				*0.470*
≤ 1.783	106/64	39.9	30.4 – 49.2	
1.783 – 2.439	107/57	47.7	38.0 – 56.7	
> 2.439	107/62	38.8	28.3 – 49.1	
Stage 4S				
Cut-off based on the median value				*0.328*
≤ 1.735	46/4	90.4	76.0 – 96.3	
> 1.735	46/7	87.8	70.7 – 92.4	
Cut-offs based on tertile values				*n.e.*
≤ 1.408	30/3	n.e.	n.e	.
1.408 – 2.097	31/2	n.e.	n.e.	
> 2.097	31/6	n.e.	n.e.	
All patients				
Cut-off based on the median value				*0.020*
≤ 1.877	393/99	74.7	70.0 – 78.7	
> 1.877	393/130	66.5	61.4 – 71.1	
Cut-offs based on tertile values				*0.047*
≤ 1.541	262/66	74.5	68.7 – 79.4	
1.541 – 2.250	262/74	72.2	66.3 – 77.3	
> 2.250	262/89	64.8	58.3 – 70.6	

N/D = Number of patients/ deaths. OS = Ten-year Overall Survival, 95%CI: 95% confidence interval of OS. n.e. = not evaluable

Table S20. Event Free Survival of 769 NB patients in relation to *CMPK2* expression levels by *MYCN* status.

Gene expression	N/E	EFS	95%CI	*p*
MYCN normal				
Cut-off based on the median value				*0.001*
≤ 1.863	308/83	73.3	68.0 – 77.9	
> 1.863	306/124	59.5	53.8 – 64.7	
Cut-offs based on tertile values				*0.003*
≤ 1.552	205/53	74.7	68.1 – 80.0	
1.552 – 2.231	206/69	66.5	59.6 – 72.5	
> 2.231	203/85	58.1	51.0 – 64.6	
MYCN amplified				
Cut-off based on the median value				*0.520*
≤ 1.954	76/58	22.9	14.0 – 33.2	
> 1.954	75/53	29.3	19.5 – 39.8	
Cut-offs based on tertile values				*0.185*
≤ 1.461	51/39	23.5	13.0 – 35.8	
1.461 – 2.320	50/38	22.8	11.9 – 35.7	
> 2.320	50/34	32.0	19.7 – 45.0	
All patients				
Cut-off based on the median value				*0.015*
≤ 1.877	387/141	63.7	58.6 – 68.3	
> 1.877	382/179	53.1	48.0 – 58.0	
Cut-offs based on tertile values				*0.042*
≤ 1.541	258/94	64.0	57.8 – 69.5	
1.541 – 2.250	258/105	59.1	52.8 – 64.8	
> 2.250	253/121	52.2	45.8 – 58.1	

N/E = Number of patients/events. EFS = Ten-year Event Free Survival, 95%CI: 95% confidence interval of EFS.

Table S21. Event Free Survival of 769 NB patients in relation to *CMPK2* expression levels by stage at diagnosis, evaluated by the Kaplan-Meier method.

Gene expression	N/E	EFS	95%CI	*p*
Localised stage				
Cut-off based on the median value				*0.685*
≤ 1.754	183/41	77.6	70.8 – 83.0	
> 1.754	181/45	75.1	68.2 – 80.8	
Cut-offs based on tertile values				*0.957*
≤ 1.396	121/31	74.4	65.6 – 81.2	
1.396 – 2.046	121/23	81.0	72.8 – 86.9	
> 2.046	122/32	73.8	65.0 – 80.7	
Stage 4				
Cut-off based on the median value				*0.110*
≤ 2.121	159/108	32.5	25.4 – 39.9	
> 2.121	155/98	36.8	29.2 – 44.3	
Cut-offs based on tertile values				*0.649*
≤ 1.783	105/68	36.2	27.1 – 45.3	
1.783 – 2.439	106/69	34.5	25.5 – 43.7	
> 2.439	103/69	33.0	24.2 – 42.1	
Stage 4S				
Cut-off based on the median value				*0.067*
≤ 1.735	46/10	78.3	63.3 – 87.7	
> 1.735	44/17	61.4	45.4 – 73.9	
Cut-offs based on tertile values				*0.080*
≤ 1.408	30/8	73.3	53.7 – 85.7	
1.408 – 2.097	30/5	83.3	64.5 – 92.7	
> 2.097	30/14	53.3	34.3 – 69.1	
All patients				
Cut-off based on the median value				*0.015*
≤ 1.877	387/141	63.7	58.6 – 68.3	
> 1.877	382/179	53.1	48.0 – 58.0	
Cut-offs based on tertile values				*0.042*
≤ 1.541	258/94	64.0	57.8 – 69.5	
1.541 – 2.250	258/105	59.1	52.8 – 64.8	
> 2.250	253/121	52.2	45.8 – 58.1	

N/E = Number of patients/events. EFS = Ten-year Event Free Survival, 95%CI: 95% confidence interval of EFS.

Table S22. Overall Survival of 786 NB patients in relation to *RSAD2* expression levels by *MYCN* status.

Gene expression	N/D	OS	95%CI	*p*
MYCN normal				
Cut-off based on the median value				*0.477*
≤ 1.662	314/56	82.0	77.1 – 86.0	
> 1.662	315/63	79.7	74.7 – 83.9	
Cut-offs based on tertile values				*0.342*
≤ 1.256	209/39	81.6	75.3 – 86.4	
1.256 – 2.094	210/33	83.8	77.9 – 88.2	
> 2.094	210/47	77.3	70.7 – 82.5	
MYCN amplified				
Cut-off based on the median value				*0.720*
≤ 1.492	76/52	31.6	21.5 – 42.1	
> 1.492	77/56	26.7	17.2 – 37.1	
Cut-offs based on tertile values				*0.762*
≤ 0.923	51/35	31.4	19.3 – 44.2	
0.923 – 2.258	51/36	29.4	17.7 – 42.1	
> 2.258	51/37	26.5	15.0 – 39.4	
All patients				
Cut-off based on the median value				*0.510*
≤ 1.655	393/110	71.9	67.0 – 76.2	
> 1.655	393/119	69.2	64.3 – 73.7	
Cut-offs based on tertile values				*0.517*
≤ 1.193	262/80	69.7	63.5 – 75.0	
1.193 – 2.144	262/61	76.3	70.5 – 81.0	
> 2.144	262/88	65.7	59.4 – 71.3	

N/D = Number of patients/ deaths. OS = Ten-year Overall Survival, 95%CI: 95% confidence interval of OS. N.e. = not evaluable

Table S23. Overall Survival of 786 NB patients in relation to *RSAD2* expression levels by stage at diagnosis, evaluated by the Kaplan-Meier method.

Gene expression	N/D	OS	95%CI	*p*
Localised stage				
Cut-off based on the median value				*0.195*
≤ 1.482	186/21	88.7	83.1 – 92.5	
> 1.482	187/14	92.5	87.6 – 95.5	
Cut-offs based on tertile values				*0.113*
≤ 1.164	124/17	86.3	78.9 – 91.2	
1.164 – 1.876	124/8	93.5	87.4 – 96.7	
> 1.876	125/10	91.9	85.5 – 95.6	
Stage 4				
Cut-off based on the median value				*0.141*
≤ 1.969	160/98	39.0	31.1 – 46.7	
> 1.969	160/85	45.7	37.3 – 53.6	
Cut-offs based on tertile values				*0.400*
≤ 1.295	106/66	38.4	28.8 – 47.9	
1.295 – 2.374	107/56	47.8	37.9 – 57.1	
> 2.374	107/61	40.8	30.8 – 50.5	
Stage 4S				
Cut-off based on the median value				*0.740*
≤ 1.474	46/5	88.3	73.8 – 95.0	
> 1.474	46/6	87.0	73.3 – 93.9	
Cut-offs based on tertile values				*n.e.*
≤ 1.014	30/4	n.e.	n.e.	
1.014 – 1.838	31/2	n.e.	n.e.	
> 1.838	31/5	n.e.	n.e.	
All patients				
Cut-off based on the median value				*0.510*
≤ 1.655	393/110	71.9	67.0 – 76.2	
> 1.655	393/119	69.2	64.3 – 73.7	
Cut-offs based on tertile values				*0.517*
≤ 1.193	262/80	69.7	63.5 – 75.0	
1.193 – 2.144	262/61	76.3	70.5 – 81.0	
> 2.144	262/88	65.7	59.4 – 71.3	

N/D = Number of patients/ deaths. OS = Ten-year Overall Survival, 95%CI: 95% confidence interval of OS. N.e. = not evaluable

Table S24. Event Free Survival of 769 NB patients in relation to *RSAD2* expression levels by *MYCN* status.

Gene expression	N/E	EFS	95%CI	*p*
MYCN normal				
Cut-off based on the median value				*0.915*
≤ 1.662	304/100	67.4	61.8 – 72.4	
> 1.662	310/107	65.5	59.9 – 70.5	
Cut-offs based on tertile values				*0.521*
≤ 1.256	201/68	66.7	59.7 – 72.7	
1.256 – 2.094	205/59	71.2	64.4 – 76.8	
> 2.094	208/80	61.5	54.6 – 67.8	
MYCN amplified				
Cut-off based on the median value				*0.975*
≤ 1.492	75/55	25.7	16.1 – 36.3	
> 1.492	76/56	26.3	17.0 – 36.5	
Cut-offs based on tertile values				*0.959*
≤ 0.923	50/36	28.0	16.5 – 40.8	
0.923 – 2.258	51/39	21.9	11.1 – 34.9	
> 2.258	50/36	28.0	16.5 – 40.8	
All patients				
Cut-off based on the median value				*0.925*
≤ 1.655	382/157	59.0	53.9 – 63.8	
> 1.655	387/163	57.9	52.8 – 62.6	
Cut-offs based on tertile values				*0.891*
≤ 1.193	253/110	56.9	50.6 – 62.8	
1.193 – 2.144	257/91	64.3	58.1 – 69.9	
> 2.144	259/119	54.1	47.8 – 59.9	

N/E = Number of patients/events. EFS = Ten-year Event Free Survival, 95%CI: 95% confidence interval of EFS.

Table S25. Event Free Survival of 769 NB patients in relation to *RSAD2* expression levels by stage at diagnosis, evaluated by the Kaplan-Meier method.

Gene expression	N/E	EFS	95%CI	*p*
Localised stage				
Cut-off based on the median value				*0.020*
≤ 1.482	181/52	71.3	64.1 – 77.3	
> 1.482	183/34	81.4	75.0 – 86.4	
Cut-offs based on tertile values				*0.012*
≤ 1.164	119/38	68.1	58.9 – 75.6	
1.164 – 1.876	123/25	79.7	71.4 – 85.8	
> 1.876	122/23	81.2	73.0 – 87.1	
Stage 4				
Cut-off based on the median value				*0.235*
≤ 1.969	155/105	32.8	25.5 – 40.2	
> 1.969	159/101	36.5	29.1 – 43.9	
Cut-offs based on tertile values				*0.741*
≤ 1.295	102/69	33.3	24.4 – 42.5	
1.295 – 2.374	106/64	39.4	30.1 – 48.6	
> 2.374	106/73	31.1	22.6 – 40.0	
Stage 4S				
Cut-off based on the median value				*0.416*
≤ 1.474	46/12	73.9	58.7 – 84.3	
> 1.474	44/15	65.9	50.0 – 77.9	
Cut-offs based on tertile values				*0.521*
≤ 1.014	30/9	70.0	50.3 – 83.1	
1.014 – 1.838	29/6	79.3	59.6 – 90.1	
> 1.838	31/12	61.3	42.0 – 75.9	
All patients				
Cut-off based on the median value				*0.925*
≤ 1.655	382/157	59.0	53.9 – 63.8	
> 1.655	387/163	57.9	52.8 – 62.6	
Cut-offs based on tertile values				*0.891*
≤ 1.193	253/110	56.9	50.6 – 62.8	
1.193 – 2.144	257/91	64.3	58.1 – 69.9	
> 2.144	259/119	54.1	47.8 – 59.9	

N/E = Number of patients/events. EFS = Ten-year Event Free Survival, 95%CI: 95% confidence interval of EFS.

Table S26. Association between *LOC339788* expression levels by outcome and main patient characteristics at diagnosis.

	LOC339788 Expression Levels				
	≤ 0.01436		> 0.01436		
**Patient characteristics**	**N**	**%**	**N**	**%**	***p***
Event					*0.291*
No	239	64.6	76	59.4	
Yes	131	35.4	52	40.6	
Dead					*0.613*
No	294	79.5	99	77.3	
Yes	76	20.5	29	22.7	
Event - MYCN Not amplified					*0.630*
No	217	70.7	64	68.1	
Yes	90	29.3	30	31.9	
Event - MYCN Amplified					*0.952*
No	21	35.0	11	34.4	
Yes	39	65.0	21	65.6	
Dead - MYCN Not amplified					*0.620*
No	265	86.3	83	88.3	
Yes	42	13.7	11	11.7	
Dead - MYCN Amplified					*0.909*
No	27	45.0	14	43.8	
Yes	33	55.0	18	56.2	
Event - Age < 18 months					*0.045*
No	182	80.5	55	69.6	
Yes	44	19.5	24	30.4	
Event - Age ≥ 18 months					*0.687*
No	57	39.6	21	42.9	
Yes	87	60.4	28	57.1	
Dead - Age < 18 months					*0.137*
No	214	94.7	71	89.9	
Yes	12	5.3	8	10.1	
Dead - Age ≥ 18 months					*0.847*
No	80	55.6	28	57.1	
Yes	64	44.4	21	42.9	
Event – Localized stage					*0.039*
No	160	82.1	47	70.2	
Yes	35	17.9	20	29.8	
Event – Stage 4					*0.629*
No	53	38.1	15	34.1	
Yes	86	61.9	29	65.9	
Event – Stage 4S					*0.511*
No	26	72.2	14	82.4	
Yes	10	27.8	3	17.6	
Dead – Localized stage					*0.086*
No	184	94.4	59	88.1	
Yes	11	5.6	8	11.9	
Dead – Stage 4					*0.921*
No	77	55.4	24	54.6	
Yes	62	44.6	20	45.4	
Dead – Stage 4S					*0.999*
No	33	91.7	16	94.1	
Yes	3	8.3	1	5.9	

Table S27. Overall Survival of 498 NB patients in relation to *LOC339788* expression levels by *MYCN* status.

Gene expression	N/D	OS	95%CI	*p*
MYCN normal				
Cut-off				*0.593*
≤ 0.01436	307/42	83.8	78.3 – 88.0	
> 0.01436	94/11	87.2	78.0 – 92.7	
MYCN amplified				
Cut-off				*0.870*
≤ 0.01436	60/33	35.7	22.0 – 49.6	
> 0.01436	32/18	39.0	21.6 – 56.0	
All patients				
Cut-off				*0.604*
≤ 0.01436	370/76	76.5	71.1 – 81.0	
> 0.01436	128/29	75.7	66.8 – 82.4	

N/D = Number of patients/ deaths. OS = Ten-year Overall Survival, 95%CI: 95% confidence interval of OS. n.e. = not evaluable. Cut-off = cut-off corresponding to the baseline expression value.

Table S28. Overall Survival of 498 NB patients in relation to *LOC339788* expression levels by stage at diagnosis, evaluated by the Kaplan-Meier method.

Gene expression	N/D	OS	95%CI	*p*
Localised stage				
Cut-off				*0.095*
≤ 0.01436	195/11	93.5	88.4 – 96.4	
> 0.01436	67/8	87.2	76.0 – 93.4	
Stage 4				
Cut-off				*0.789*
≤ 0.01436	139/62	47.4	37.1 – 57.0	
> 0.01436	44/20	48.4	31.9 – 63.1	
Stage 4S				
Cut-off				*n.e.*
≤ 0.01436	36/3	n.e.	n.e.	
> 0.01436	17/1	n.e.	n.e.	
All patients				
Cut-off				*0.604*
≤ 0.01436	370/76	76.5	71.1 – 81.0	
> 0.01436	128/29	75.7	66.8 – 82.4	

N/D = Number of patients/ deaths. OS = Ten-year Overall Survival, 95%CI: 95% confidence interval of OS. n.e. = not evaluable. Cut-off = cut-off corresponding to the baseline expression value.

Table S29. Event Free Survival of 498 NB patients in relation to *LOC339788* expression levels by *MYCN* status.

Gene expression	N/E	EFS	95%CI	*p*
MYCN normal				
Cut-off				*0.572*
≤ 0.01436	307/90	69.5	63.9 – 74.5	
> 0.01436	94/30	66.4	55.5 – 75.2	
MYCN amplified				
Cut-off				*0.860*
≤ 0.01436	60/39	27.5	15.9 – 40.4	
> 0.01436	32/21	32.3	16.9 – 48.6	
All patients				
Cut-off				*0.253*
≤ 0.01436	370/131	63.0	57.7 – 67.9	
> 0.01436	128/52	57.5	48.2 – 65.8	

N/E = Number of patients/ events. EFS = Ten-year Event Free Survival, 95%CI: 95% confidence interval of EFS. n.e. = not evaluable. Cut-off = cut-off corresponding to the baseline expression value.

Table S30. Event Free Survival of 498 NB patients in relation to *LOC339788* expression levels by stage at diagnosis, evaluated by the Kaplan-Meier method.

Gene expression	N/E	EFS	95%CI	*p*
Localised stage				
Cut-off				*0.036*
≤ 0.01436	195/35	81.6	75.3 – 86.4	
> 0.01436	67/20	69.6	56.9 – 79.2	
Stage 4				
Cut-off				*0.423*
≤ 0.01436	139/86	34.0	25.7 – 42.4	
> 0.01436	44/29	26.8	14.1 – 41.4	
Stage 4S				
Cut-off				*0.420*
≤ 0.01436	36/10	71.4	53.3 – 83.5	
> 0.01436	17/3	82.4	54.7 – 93.9	
All patients				
Cut-off				*0.253*
≤ 0.01436	370/131	63.0	57.7 – 67.9	
> 0.01436	128/52	57.5	48.2 – 65.8	

N/E = Number of patients/ events. OS = Ten-year Overall Survival, 95%CI: 95% confidence interval of OS. n.e. = not evaluable. Cut-off = cut-off corresponding to the based on the baseline expression value.

Table S31. Overall Survival of 786 NB patients in relation to *GREB1* expression levels by *MYCN* status.

**Gene expression**	**N/D**	**OS**	**95%CI**	***p***
MYCN normal				
Cut-off based on the median value				*0.004*
≤ 3.994	314/74	75.5	70.1 – 80.0	
> 3.994	315/45	86.2	81.7 – 89.7	
Cut-offs based on tertile values				*< 0.001*
≤ 3.747	209/59	70.7	63.7 – 76.6	
3.747 – 4.259	210/32	84.9	79.2 – 89.2	
≤ 4.259	210/28	86.9	81.2 – 91.0	
MYCN amplified				
Cut-off based on the median value				*0.835*
≤ 4.204	76/53	29.2	19.0 – 40.0	
> 4.204	77/55	28.6	19.0 – 38.9	
Cut-offs based on tertile values				*0.991*
≤ 3.981	51/36	31.4	19.3 – 44.2	
3.981 – 4.463	51/36	29.4	17.7 – 42.1	
≤ 4.463	51/36	29.4	17.7 – 42.1	
All patients				
Cut-off based on the median value				*0.811*
≤ 4.029	393/114	70.1	65.2 – 74.5	
> 4.029	393/115	71.0	66.1 – 75.3	
Cut-offs based on tertile values				*0.400*
≤ 3.791	262/89	65.0	58.6 – 70.6	
3.791 – 4.311	262/62	76.5	70.8 – 81.2	
> 4.311	262/78	70.2	64.1 – 75.5	

N/D = Number of patients/ deaths. OS = Ten-year Overall Survival, 95%CI: 95% confidence interval of OS. N.e. = not evaluable.

Table S32. Overall Survival of 786 NB patients in relation to *GREB1* expression levels by stage at diagnosis, evaluated by the Kaplan-Meier method.

Gene expression	N/D	OS	95%CI	*p*
Localised stage				
Cut-off based on the median value				*0.226*
≤ 4.059	186/21	88.6	83.1 – 92.4	
> 4.059	187/14	92.5	87.7 – 95.5	
Cut-offs based on tertile values				*0.032*
≤ 3.831	124/18	85.4	77.8 – 90.5	
3.831 – 3.831	124/9	92.7	86.5 – 96.2	
> 3.831	125/8	93.6	87.6 – 96.8	
Stage 4				
Cut-off based on the median value				*0.191*
≤ 3.990	160/87	43.6	35.3 – 51.5	
> 3.990	160/96	41.1	33.3 – 48.8	
Cut-offs based on tertile values				*0.207*
≤ 3.625	106/60	41.2	31.2 – 51.0	
3.625 – 4.277	107/55	48.4	38.4 – 57.8	
> 4.277	107/68	37.4	28.1 – 46.7	
Stage 4S				
Cut-off based on the median value				*0.798*
≤ 4.203	46/5	89.1	75.8 – 95.3	
> 4.203	46/6	86.3	71.7 – 93.6	
Cut-offs based on tertile values				*n.e.*
≤ 3.877	30/2	n.e.	n.e.	
3.877 – 4.391	31/4	n.e.	n.e.	
> 4.391	31/5	n.e.	n.e.	
All patients				
Cut-off based on the median value				*0.811*
≤ 4.029	393/114	70.1	65.2 – 74.5	
> 4.029	393/115	71.0	66.1 – 75.3	
Cut-offs based on tertile values				*0.400*
≤ 3.791	262/89	65.0	58.6 – 70.6	
3.791 – 4.311	262/62	76.5	70.8 – 81.2	
> 4.311	262/78	70.2	64.1 – 75.5	

N/D = Number of patients/ deaths. OS = Ten-year Overall Survival, 95%CI: 95% confidence interval of OS. N.e. = not evaluable

Table S33. Event Free Survival of 769 NB patients in relation to *GREB1* expression levels by *MYCN* status.

**Gene expression**	**N/E**	**OS**	**95%CI**	***p***
MYCN normal				
Cut-off based on the median value				*0.017*
≤ 3.994	306/119	61.1	55.4 – 66.3	
> 3.994	308/88	71.8	66.4 – 76.4	
Cut-offs based on tertile values				*< 0.001*
≤ 3.747	202/97	51.9	44.8 – 58.6	
3.747 – 4.259	207/54	73.9	67.4 – 79.4	
> 4.259	205/56	73.2	66.5 – 78.7	
MYCN amplified				
Cut-off based on the median value				*0.551*
≤ 4.204	75/56	24.0	14.6 – 34.8	
> 4.204	76/55	27.6	18.2 – 37.9	
Cut-offs based on tertile values				*0.907*
≤ 3.981	50/37	26.0	14.9 – 38.6	
3.981 – 4.463	50/37	24.5	13.1 – 37.7	
> 4.463	51/37	27.5	16.1 – 40.0	
All patients				
Cut-off based on the median value				*0.398*
≤ 4.029	384/167	56.3	51.2 – 61.2	
> 4.029	385/153	60.5	55.5 – 65.2	
Cut-offs based on tertile values				*0.049*
≤ 3.791	253/127	49.8	43.5 – 55.7	
3.791 – 4.311	260/91	65.2	59.0 – 70.7	
> 4.311	256/102	60.2	53.9 -65.9	

N/E = Number of patients/ events. EFS = Ten-year Event Free Survival, 95%CI: 95% confidence interval of EFS. n.e. = not evaluable.

Table S34. Event Free Survival of 769 NB patients in relation to *GREB1* expression levels by stage at diagnosis, evaluated by the Kaplan-Meier method.

**Gene expression**	**N/E**	**EFS**	**95%CI**	***p***
Localised stage				
Cut-off based on the median value				*0.270*
≤ 4.086	182/48	73.6	66.6 – 79.4	
> 4.086	182/38	79.1	72.4 – 84.3	
Cut-offs based on tertile values				*0.050*
≤ 3.831	120/38	68.3	59.2 – 75.8	
3.831 – 4.311	122/24	80.3	72.1 – 86.4	
> 4.311	122/24	80.3	72.1 – 86.4	
Stage 4				
Cut-off based on the median value				*0.758*
≤ 3.990	157/103	34.4	27.1 – 41.8	
> 3.990	157/103	34.8	27.4 – 42.3	
Cut-offs based on tertile values				*0.719*
≤ 3.625	103/74	28.2	19.9 – 37.0	
3.625 – 4.277	107/62	41.7	32.2 – 50.9	
> 4.277	104/70	33.7	24.8 – 42.7	
Stage 4S				
Cut-off based on the median value				*0.962*
≤ 4.203	44/13	70.5	54.6 – 81.7	
> 4.203	46/14	69.6	54.1 – 80.7	
Cut-offs based on tertile values				*0.724*
≤ 3.877	28/7	75.0	54.6 – 87.2	
3.877 – 4.391	31/11	64.5	45.2 – 78.5	
> 4.391	31/9	71.0	51.6 – 83.7	
All patients				
Cut-off based on the median value				*0.398*
≤ 4.029	384/167	56.3	51.2 – 61.2	
> 4.029	385/153	60.5	55.5 – 65.2	
Cut-offs based on tertile values				*0.049*
≤ 3.791	253/127	49.8	43.5 – 55.7	
3.791 – 4.311	260/91	65.2	59.0 – 70.7	
> 4.311	256/102	60.2	53.9 -65.9	

N/E = Number of patients/events. EFS = Ten-year Event Free Survival, 95%CI: 95% confidence interval of EFS.

Table S35. Overall Survival of 709 NB patients in relation to *NTSR2* expression levels by *MYCN* status.

Gene expression	N/D	OS	95%CI	*p*
MYCN normal				
Cut-off based on the median value				*0.098*
≤ -0.917	290/47	80.8	74.9 – 85.5	
> -0.917	291/36	86.2	80.9 – 90.1	
Cut-offs based on tertile values				*0.464*
≤ -1.255	193/31	79.8	71.9 – 85.7	
-1.255 – -0.599	194/23	87.1	80.8 – 91.4	
≤ -0.599	194/29	83.5	76.4 – 88.6	
MYCN amplified				
Cut-off based on the median value				*0.973*
≤ -1.176	61/37	33.9	21.4 – 46.8	
> -1.176	61/39	34.0	22.2 – 46.1	
Cut-offs based on tertile values				*0.757*
≤ -1.724	40/23	34.5	18.7 – 50.9	
-1.724 – -0.643	41/26	35.2	20.9 – 49.7	
≤ -0.643	41/27	32.6	18.6 – 47.3	
All patients				
Cut-off based on the median value				*0.033*
≤ -0.944	354/90	71.0	65.2 – 76.0	
> -0.944	355/71	78.4	73.2 – 82.7	
Cut-offs based on tertile values				*0.196*
≤ -1.303	236/62	69.0	61.5 – 75.3	
-1.303 – -0.594	236/44	79.7	73.4 – 84.7	
> -0.594	237/55	75.2	68.5 – 80.7	

N/D = Number of patients/ deaths. OS = Ten-year Overall Survival, 95%CI: 95% confidence interval of OS. N.e. = not evaluable.

Table S36. Overall Survival of 709 NB patients in relation to *NTSR2* expression levels by stage at diagnosis, evaluated by the Kaplan-Meier method.

Gene expression	N/D	OS	95%CI	*p*
Localised stage				
Cut-off based on the median value				*0.098*
≤ -0.870	185/17	89.3	83.2 – 93.3	
> -0.870	185/9	94.7	90.0 – 97.2	
Cut-offs based on tertile values				*0.036*
≤ -1.208	123/14	86.2	77.5 – 91.7	
-1.208 – -0.545	123/6	94.4	87.9 – 97.5	
> -0.545	124/6	95.0	89.2 – 97.7	
Stage 4				
Cut-off based on the median value				*0.361*
≤ -1.035	129/64	44.2	33.9 – 54.1	
> -1.035	130/65	48.5	39.0 – 57.4	
Cut-offs based on tertile values				*0.379*
≤ -1.363	86/44	41.5	28.8 – 53.7	
-1.363 – -0.558	86/40	50.6	38.4 – 61.6	
> -0.558	87/45	47.3	35.7 – 58.1	
Stage 4S				
Cut-off based on the median value				*n.e.*
≤ -1.075	40/3	n.e.	n.e.	
> -1.075	40/3	n.e.	n.e.	
Cut-offs based on tertile values				*n.e.*
≤ -1.526	26/1	n.e.	n.e.	
-1.526 – 0.870	27/3	n.e.	n.e.	
> -0.870	27/2	n.e.	n.e.	
All patients				
Cut-off based on the median value				*0.033*
≤ -0.944	354/90	71.0	65.2 – 76.0	
> -0.944	355/71	78.4	73.2 – 82.7	
Cut-offs based on tertile values				*0.196*
≤ -1.303	236/62	69.0	61.5 – 75.3	
-1.303 – -0.594	236/44	79.7	73.4 – 84.7	
> -0.594	237/55	75.2	68.5 – 80.7	

N/D = Number of patients/ deaths. OS = Ten-year Overall Survival, 95%CI: 95% confidence interval of OS. N.e. = not evaluable

Table S37. Event Free Survival of 695 NB patients in relation to *NTSR2* expression levels by *MYCN* status.

Gene expression	N/E	EFS	95%CI	*p*
MYCN normal				
Cut-off based on the median value				*0.095*
≤ -0.917	286/92	66.4	60.4 – 71.7	
> -0.917	282/76	72.2	66.2 – 77.4	
Cut-offs based on tertile values				*0.157*
≤ -1.255	189/61	66.3	58.8 – 72.7	
-1.255 – -0.599	193/55	70.7	63.5 – 76.7	
> -0.599	186/52	71.1	63.3 – 77.5	
MYCN amplified				
Cut-off based on the median value				*0.623*
≤ -1.176	60/43	25.7	15.2 – 37.6	
> -1.176	61/41	29.9	18.3 – 42.3	
Cut-offs based on tertile values				*0.993*
≤ -1.724	39/26	29.2	15.3 – 44.5	
-1.724 – -0.643	41/30	25.2	13.1 – 39.3	
> -0.643	41/28	29.7	16.1 – 44.6	
All patients				
Cut-off based on the median value				*0.066*
≤ -0.944	349/138	58.8	53.3 – 64.0	
> -0.944	346/118	64.8	59.2 – 69.8	
Cut-offs based on tertile values				*0.056*
≤ -1.303	231/96	56.6	49.7 – 62.9	
-1.303 – 0.594	235/79	65.6	59.0 – 71.3	
> -0.594	229/81	63.3	56.1 – 69.6	

N/E = Number of patients/ events. OS = Ten-year Overall Survival, 95%CI: 95% confidence interval of OS. N.e. = not evaluable.

Table S38. Event Free Survival of 695 NB patients in relation to *NTSR2* expression levels by stage at diagnosis, evaluated by the Kaplan-Meier method.

Gene expression	N/E	EFS	95%CI	*p*
Localised stage				
Cut-off based on the median value				*0.003*
≤ -0.870	180/49	72.3	65.1 – 78.3	
> -0.870	179/27	84.4	78.1 – 89.1	
Cut-offs based on tertile values				*0.038*
≤ -1.208	119/32	72.2	63.0 – 79.5	
-1.208 – -0.545	122/25	79.3	70.9 – 85.5	
> -0.545	118/19	83.5	75.3 – 89.1	
Stage 4				
Cut-off based on the median value				*0.400*
≤ -1.035	129/81	34.5	26.1 – 43.0	
> -1.035	129/82	35.8	27.1 – 44.6	
Cut-offs based on tertile values				*0.598*
≤ -1.363	86/54	34.2	23.9 – 44.6	
-1.363 – -0.558	86/52	38.8	28.5 – 49.0	
> -0.558	86/57	32.4	21.7 – 43.6	
Stage 4S				
Cut-off based on the median value				*0.750*
≤ -1.075	39/9	76.3	59.4 – 86.9	
> -1.075	39/8	78.9	62.1 – 88.9	
Cut-offs based on tertile values				*0.931*
≤ -1.526	25/4	83.2	61.1 – 93.4	
-1.526 – 0.870	27/9	64.5	42.1 – 80.1	
> -0.870	26/4	84.6	64.0 – 93.9	
All patients				
Cut-off based on the median value				*0.066*
≤ -0.944	349/138	58.8	53.3 – 64.0	
> -0.944	346/118	64.8	59.2 – 69.8	
Cut-offs based on tertile values				*0.056*
≤ -1.303	231/96	56.6	49.7 – 62.9	
-1.303 – 0.594	235/79	65.6	59.0 – 71.3	
> -0.594	229/81	63.3	56.1 – 69.6	

N/E = Number of patients/events. EFS = Ten-year Event Free Survival, 95%CI: 95% confidence interval of EFS.

Table S39. Overall Survival of 786 NB patients in relation to *LPIN1* expression levels by *MYCN* status.

Gene expression	N/D	OS	95%CI	*p*
MYCN normal				
Cut-off based on the median value				*< 0.001*
≤ 4.340	314/87	71.8	66.3 – 76.6	
> 4.340	315/32	90.1	86.1 – 92.9	
Cut-offs based on tertile values				*< 0.001*
≤ 4.136	209/70	66.1	59.0 – 72.3	
4.136 – 4.537	210/31	85.0	79.4 – 89.2	
> 4.537	210/18	91.9	87.3 – 94.9	
MYCN amplified				
Cut-off based on the median value				*0.541*
≤ 4.180	76/51	32.1	21.7 – 42.9	
> 4.180	77/57	26.0	16.8 – 36.1	
Cut-offs based on tertile values				*0.799*
≤ 3.974	51/39	25.5	14.6 – 37.9	
3.974 – 4.368	51/30	41.2	27.7 – 54.2	
≤ 4.368	51/39	23.5	13.0 – 35.8	
All patients				
Cut-off based on the median value				*< 0.001*
≤ 4.313	393/149	61.5	56.4 – 66.3	
> 4.313	393/80	79.7	75.3 – 83.4	
Cut-offs based on tertile values				*< 0.001*
≤ 4.102	262/113	56.2	49.7 – 62.2	
4.102 – 4.512	262/67	74.2	68.5 – 79.1	
> 4.512	262/49	81.5	76.2 – 85.7	

N/D = Number of patients/ deaths. OS = Ten-year Overall Survival, 95%CI: 95% confidence interval of OS. N.e. = not evaluable.

Table S40. Overall Survival of 786 NB patients in relation to *LPIN1* expression levels by stage at diagnosis, evaluated by the Kaplan-Meier method.

Gene expression	N/D	OS	95%CI	*p*
Localised stage				
Cut-off based on the median value				*< 0.001*
≤ 4.410	186/29	84.3	78.2 – 88.8	
> 4.410	187/6	96.8	93.0 – 98.6	
Cut-offs based on tertile values				*< 0.001*
≤ 4.216	124/26	78.9	70.5 – 85.1	
4.216 – 4.599	124/4	96.8	91.6 – 98.8	
> 4.599	125/5	96.0	90.7 – 98.3	
Stage 4				
Cut-off based on the median value				*0.934*
≤ 4.150	160/93	40.3	32.0 – 48.5	
> 4.150	160/90	44.2	36.3 – 51.7	
Cut-offs based on tertile values				*0.214*
≤ 3.928	106/69	32.2	22.7 – 42.2	
3.928 – 4.350	107/55	49.5	39.8 – 58.6	
> 4.350	107/59	45.6	35.9 – 54.7	
Stage 4S				
Cut-off based on the median value				*0.687*
≤ 4.402	46/6	87.0	73.3 – 93.9	
> 4.402	46/5	88.4	74.0 – 95.0	
Cut-offs based on tertile values				*0.588*
≤ 4.257	30/4	86.7	68.3 – 94.8	
4.257 – 4.574	31/4	84.3	61.7 – 94.1	
> 4.574	31/3	90.3	72.9 – 96.8	
All patients				
Cut-off based on the median value				*< 0.001*
≤ 4.313	393/149	61.5	56.4 – 66.3	
> 4.313	393/80	79.7	75.3 – 83.4	
Cut-offs based on tertile values				*< 0.001*
≤ 4.102	262/113	56.2	49.7 – 62.2	
4.102 – 4.512	262/67	74.2	68.5 – 79.1	
> 4.512	262/49	81.5	76.2 – 85.7	

N/D = Number of patients/deaths. OS = Ten-year Overall Survival, 95%CI: 95% confidence interval of OS. N.e. = not evaluable.

Table S41. Event Free Survival of 769 NB patients in relation to *LPIN1* expression levels by *MYCN* status.

Gene expression	N/E	OS	95%CI	*p*
MYCN normal				
Cut-off based on the median value				*< 0.001*
≤ 4.340	299/127	57.8	52.0 – 63.2	
> 4.340	315/80	74.6	69.4 – 79.1	
Cut-offs based on tertile values				*< 0.001*
≤ 4.136	194/96	51.0	43.7 – 57.8	
4.136 – 4.537	210/60	71.4	64.8 – 77.0	
≤ 4.537	210/51	75.7	69.3 – 81.0	
MYCN amplified				
Cut-off based on the median value				*0.374*
≤ 4.180	74/51	31.1	21.0 – 41.7	
> 4.180	77/60	20.8	12.1 – 31.1	
Cut-offs based on tertile values				*0.823*
≤ 3.974	49/38	22.5	12.1 – 34.8	
3.974 – 4.368	51/33	33.5	20.3 – 47.3	
≤ 4.368	51/40	21.6	11.6 – 33.6	
All patients				
Cut-off based on the median value				*< 0.001*
≤ 4.313	376/189	49.8	44.7 – 54.8	
> 4.313	393/131	66.7	61.8 – 71.1	
Cut-offs based on tertile values				*< 0.001*
≤ 4.102	245/135	45.3	39.0 – 51.4	
4.102 – 4.512	262/98	62.3	56.1 – 67.9	
> 4.512	262/87	66.8	60.7 – 72.1	

N/E = Number of patients/ events. EFS = Ten-year Event Free Survival, 95%CI: 95% confidence interval of EFS. n.e. = not evaluable.

Table S42. Event Free Survival of 769 NB patients in relation to *LPIN1* expression levels by stage at diagnosis, evaluated by the Kaplan-Meier method.

Gene expression	N/E	EFS	95%CI	*p*
Localised stage				
Cut-off based on the median value				*0.066*
≤ 4.410	177/50	71.8	64.5 – 77.8	
> 4.410	187/36	80.8	74.3 – 85.7	
Cut-offs based on tertile values				*0.009*
≤ 4.216	115/38	67.0	57.6 – 74.7	
4.216 – 4.599	124/26	79.0	70.8 – 85.2	
> 4.599	125/22	82.4	74.5 – 88.0	
Stage 4				
Cut-off based on the median value				*0.829*
≤ 4.150	154/103	33.8	26.4 – 41.2	
> 4.150	160/103	35.3	28.0 – 42.8	
Cut-offs based on tertile values				*0.175*
≤ 3.928	100/73	27.7	18.7 – 35.9	
3.928 – 4.350	107/65	39.6	30.2 – 48.9	
> 4.350	107/68	36.5	27.4 – 45.5	
Stage 4S				
Cut-off based on the median value				*0.337*
≤ 4.402	44/15	65.9	50.0 – 77.9	
> 4.402	46/12	73.9	58.7 – 84.3	
Cut-offs based on tertile values				*0.070*
≤ 4.257	28/11	60.7	40.4 – 76.0	
4.257 – 4.574	31/10	67.7	48.4 – 81.2	
> 4.574	31/6	80.7	61.9 – 90.8	
All patients				
Cut-off based on the median value				*< 0.001*
≤ 4.313	376/189	49.8	44.7 – 54.8	
> 4.313	393/131	66.7	61.8 – 71.1	
Cut-offs based on tertile values				*< 0.001*
≤ 4.102	245/135	45.3	39.0 – 51.4	
4.102 – 4.512	262/98	62.3	56.1 – 67.9	
> 4.512	262/87	66.8	60.7 – 72.1	

N/E = Number of patients/events. EFS = Ten-year Event Free Survival, 95%CI: 95% confidence interval of EFS.

Table S43. Overall Survival of 786 NB patients in relation to *NBAS* expression levels by *MYCN* status.

Gene expression	N/D	OS	95%CI	*p*
MYCN normal				
Cut-off based on the median value				*0.709*
≤ 3.275	314/58	81.3	76.2 – 85.3	
> 3.275	315/61	80.6	75.7 – 84.6	
Cut-offs based on tertile values				*0.438*
≤ 3.065	209/43	78.8	72.3 – 84.0	
3.065 – 3.518	210/40	80.7	74.5 – 85.6	
≤ 3.518	210/36	83.3	77.6 – 87.7	
MYCN amplified				
Cut-off based on the median value				*0.154*
≤ 3.798	76/50	33.2	22.5 – 44.2	
> 3.798	77/58	24.7	15.7 – 34.7	
Cut-offs based on tertile values				*0.361*
≤ 3.129	51/30	41.2	27.7 – 54.2	
3.129 – 4.487	51/42	17.2	8.2 – 28.8	
≤ 4.487	51/36	29.4	17.7 – 42.1	
All patients				
Cut-off based on the median value				*0.010*
≤ 3.311	393/99	74.4	69.5 – 78.5	
> 3.311	393/130	66.8	61.9 – 71.3	
Cut-offs based on tertile values				*0.007*
≤ 3.074	261/71	72.1	66.0 – 77.4	
3.074 – 3.585	263/62	76.3	70.5 – 81.2	
> 3.585	262/96	63.4	57.2 – 68.9	

N/D = Number of patients/ deaths. OS = Ten-year Overall Survival, 95%CI: 95% confidence interval of OS. n.e. = not evaluable.

Table S44. Overall Survival of 786 NB patients in relation to *NBAS* expression levels by stage at diagnosis, evaluated by the Kaplan-Meier method.

Gene expression	N/D	OS	95%CI	*p*
Localised stage				
Cut-off based on the median value				*0.854*
≤ 3.264	186/18	90.3	85.0 – 93.8	
> 3.264	187/17	90.9	85.7 – 94.2	
Cut-offs based on tertile values				*0.282*
≤ 3.092	124/15	87.9	80.7 – 92.5	
3.092 – 3.504	124/10	91.8	85.3 – 95.5	
> 3.504	125/10	92.0	85.6 – 95.6	
Stage 4				
Cut-off based on the median value				*0.081*
≤ 3.387	160/85	46.1	37.8 – 54.1	
> 3.387	160/98	38.7	31.1 – 46.3	
Cut-offs based on tertile values				*0.018*
≤ 3.042	106/52	48.9	38.0 – 58.9	
3.042 – 3.770	107/64	40.8	31.1 – 50.2	
> 3.770	107/67	37.4	28.3 – 46.5	
Stage 4S				
Cut-off based on the median value				*0.369*
≤ 3.337	45/4	89.7	74.2 – 96.2	
> 3.337	47/7	85.1	71.3 – 92.6	
Cut-offs based on tertile values				*0.245*
≤ 3.127	30/3	90.0	72.1 – 96.7	
3.127 – 3.597	31/2	92.2	71.5 – 98.0	
> 3.597	31/6	80.7	61.9 – 90.8	
All patients				
Cut-off based on the median value				*0.010*
≤ 3.311	393/99	74.4	69.5 – 78.5	
> 3.311	393/130	66.8	61.9 – 71.3	
Cut-offs based on tertile values				*0.007*
≤ 3.074	261/71	72.1	66.0 – 77.4	
3.074 – 3.585	263/62	76.3	70.5 – 81.2	
> 3.585	262/96	63.4	57.2 – 68.9	

N/D = Number of patients/deaths. OS = Ten-year Overall Survival, 95%CI: 95% confidence interval of OS. n.e. = not evaluable.

Table S45. Event Free Survival of 769 NB patients in relation to *NBAS* expression levels by *MYCN* status.

Gene expression	N/E	EFS	95%CI	*p*
MYCN normal				
Cut-off based on the median value				*0.791*
≤ 3.311	299/100	66.9	61.2 – 71.9	
> 3.311	315/107	66.0	60.5 – 71.0	
Cut-offs based on tertile values				*0.730*
≤ 3.065	194/66	66.5	59.3 – 72.6	
3.065 – 3.518	210/76	63.8	56.9 – 69.9	
≤ 3.518	210/65	69.1	62.3 – 74.8	
MYCN amplified				
Cut-off based on the median value				*0.113*
≤ 3.798	74/51	29.2	18.5 – 40.7	
> 3.798	77/60	22.1	13.6 – 31.9	
Cut-offs based on tertile values				*0.259*
≤ 3.129	49/31	32.3	17.2 – 48.5	
3.129 – 4.487	51/42	17.7	8.7 – 29.2	
≤ 4.487	51/38	25.5	14.6 – 37.9	
All patients				
Cut-off based on the median value				*0.063*
≤ 3.311	376/146	61.3	56.1 – 66.0	
> 3.311	393/174	55.7	50.7 – 60.5	
Cut-offs based on tertile values				*0.008*
≤ 3.074	244/95	61.2	54.8 – 67.1	
3.074 – 3.585	263/99	62.4	56.2 – 67.9	
> 3.585	262/126	51.9	45.7 – 57.8	

N/E = Number of patients/ events. EFS = Ten-year Event Free Survival, 95%CI: 95% confidence interval of EFS. n.e. = not evaluable.

Table S46. Event Free Survival of 769 NB patients in relation to *NBAS* expression levels by stage at diagnosis, evaluated by the Kaplan-Meier method.

Gene expression	N/E	EFS	95%CI	*p*
Localised stage				
Cut-off based on the median value				*0.610*
≤ 3.264	177/40	77.4	70.5 – 82.9	
> 3.264	187/46	75.4	68.6 – 81.0	
Cut-offs based on tertile values				*0.547*
≤ 3.092	115/24	79.1	70.5 – 85.5	
3.092 –3.504	124/33	73.4	64.7 – 80.3	
> 3.504	125/29	76.8	68.4 – 83.3	
Stage 4				
Cut-off based on the median value				*0.490*
≤ 3.387	154/100	35.4	27.8 – 43.0	
> 3.387	160/106	33.8	26.5 – 41.1	
Cut-offs based on tertile values				*0.201*
≤ 3.042	100/62	38.4	28.7 – 47.9	
3.042 – 3.770	107/73	31.8	23.2 – 40.7	
> 3.770	107/71	33.6	24.9 – 42.6	
Stage 4S				
Cut-off based on the median value				*0.599*
≤ 3.337	43/12	72.1	56.1 – 83.1	
> 3.337	47/15	68.1	52.8 – 79.4	
Cut-offs based on tertile values				*0.487*
≤ 3.127	28/8	71.4	50.9 – 84.6	
3.127 – 3.597	31/8	74.2	55.0 – 86.2	
> 3.597	31/11	64.5	45.2 – 78.5	
All patients				
Cut-off based on the median value				*0.063*
≤ 3.311	376/146	61.3	56.1 – 66.0	
> 3.311	393/174	55.7	50.7 – 60.5	
Cut-offs based on tertile values				*0.008*
≤ 3.074	244/95	61.2	54.8 – 67.1	
3.074 – 3.585	263/99	62.4	56.2 – 67.9	
> 3.585	262/126	51.9	45.7 – 57.8	

N/E = Number of patients/events. EFS = Ten-year Event Free Survival, 95%CI: 95% confidence interval of EFS.
